# Validation of the compassionate engagement and action scales, compassion scale, and Sussex-Oxford compassion scales in a French-Canadian sample

**DOI:** 10.1371/journal.pone.0305776

**Published:** 2024-06-24

**Authors:** Kyla Brophy, Matthew Emery, Ceilagh MacDonald, Catherine Isadora Côté, Annett Körner

**Affiliations:** 1 Department of Counselling Psychology, McGill University, Montréal, Quebec, Canada; 2 Department of Statistics, University of British Columbia, Vancouver, British Columbia, Canada; 3 Department of Psychology, University of Montreal, Montréal, Quebec, Canada; 4 Lady Davis Institute, Jewish General Hospital, Montréal, Quebec, Canada; 5 Department of Oncology, McGill University, Montréal, Quebec, Canada; 6 Louise Granofsky Psychosocial Oncology Program, Segal Cancer Center, Montréal, Quebec, Canada; 7 Psychosocial Oncology Program, McGill University Health Centre, Montréal, Quebec, Canada; Queen Mary University of London School of Business and Management, UNITED KINGDOM

## Abstract

Compassion towards oneself and towards others has been associated with positive psychological outcomes, however, research is limited by the availability of valid psychometric measures, particularly in languages other than English. The current study translated (English to French) and validated the following measures: the Compassionate Engagement and Action Scales (CEAS), assessing self-compassion (CEAS-SC), compassion to others (CEAS-TO), and compassion from others (CEAS-FROM); the Compassion Scale (CS); and the Sussex-Oxford Compassion Scales for Self (SOCS-S) and Others (SOCS-O). French-speaking participants were recruited online (*N* = 384) and completed the translated measures as well as questionnaires assessing self-compassion, depression, anxiety, stress, insecure attachment, mindfulness, and well-being. Confirmatory Factor Analysis supports the original factor structures proposed for the CEAS-FROM (two-factor hierarchical), CS (four-factor hierarchical), SOCS-S and SOCS-O (five-factor hierarchical), with alternate factor structures proposed for CEAS-SC (three-factor) and CEAS-TO (two-factor). Results showed good internal consistency and convergent validity for all scales, supporting the use of total scores for the translated measures.

## Introduction

Compassion, and particularly self-compassion, has received significant attention in Western psychological research over the past 15 years [1–4]. Prior to this recent proliferation in scientific interest, the concept of compassion has appeared across multiple philosophical, contemplative, and religious traditions for thousands of years [5–8]. Many conceptualizations of compassion within Western psychology reference Buddhist origins [2,4,9–11], particularly concepts such as being open to and connecting with suffering.

One of the reasons for the scientific interest in compassion is that it has been associated with a number of positive outcomes in psychological research, including: optimism, creativity, goal pursuit, job satisfaction, and well-being [12–17]. Self-compassion has been found to buffer symptoms of depression, stress and insecure attachment, and enhance resiliency [18–20]. There is also growing evidence that compassion for self and others can be cultivated through compassion-based interventions, and can be a beneficial target in psychotherapy and mental health programs [21–23].

Despite this promising evidence, there is a lack of consensus on how to define and measure compassion [2,24–27], as well as emerging evidence on cultural and linguistic differences [28,29]. Most definitions contain an affective component in terms of *feeling* for a person who is suffering—for example, Lazarus [30] defined compassion as “being moved by another’s suffering and wanting to help” (p. 289), and Goetz et al. [31] argued that compassion is a distinct emotion, defined as “the feeling that arises in witnessing another’s suffering and that motivates a subsequent desire to help” (p. 351). However, affective, cognitive, and behavioural (or motivational) components vary across different approaches, and are evident in the varied measurement tools that arise from different models. Psychometric studies evaluating measurement tools with diverse populations are essential for advancing the science of compassion. This article reports on the translation (from English to French), validation, and psychometric properties of three measures of compassion: the Compassionate Engagement and Action Scales (CEAS; [32]), the Compassion Scale (CS; [33]), and the Sussex-Oxford Compassion Scales (SOCS; [34]). Each of these scales is based on a distinct theoretical approach, informing the definition, model, and subsequent use of the questionnaires.

### Compassionate engagement and action scales

The Compassionate Engagement and Action Scales(CEAS; [32]) are based on Gilbert’s evolutionary model of compassion [1,35]. Gilbert [36] conceptualizes compassion as a motivation, defining it as “a sensitivity to suffering in self and others with a commitment to try to alleviate and prevent it” [32, p. 1]. This definition involves two underlying processes presented in a stimulus-response algorithm: first, an awareness or engagement with suffering, and then the motivation or intention to act. Each of these processes (engagement and action) involve distinct skills and competencies. For example, to engage with suffering requires attention sensitivity, empathy, and distress tolerance, while compassionate action requires the skills and wisdom to respond effectively to alleviate suffering [1].

Another defining feature of Gilbert’s model of compassion is understanding the “dynamic reciprocal processing nature of compassion” [37, pp. 26–27], referred to as the flow of compassion [1]. In this model, compassion can be directed from self to other (compassion for others), other to self (compassion from others), and self to self (self-compassion), reflecting inter- and intrapersonal dimensions. Examining the relationship between these different orientations of compassion is particularly relevant in clinical settings, as it has been observed that it is often more difficult for individuals to be compassionate towards themselves than it is to be compassionate towards others [1,38,39].

Given these important defining features of Gilbert’s model, the CEAS consists of three scales, assessing each orientation of the flow of compassion (i.e., compassion for others, compassion from others, and compassion for self). It is proposed that each scale can be used to provide a single-factor score, as well as subscale scores assessing engagement and action [32,40]. The engagement competencies include motivation to care for well-being, attention and/or sensitivity to suffering, sympathy, distress tolerance, empathy, and being accepting and non-judgmental [32]. The action competencies include directing attention to what is helpful, thinking and reasoning about what is likely to be helpful, taking helpful actions, and creating inner feelings of support, kindness, helpfulness and encouragement to deal with distress [32]. Each of the three scales consists of 13 items: six items assessing compassionate engagement, four items assessing compassionate action, and three reverse-formulated items intended to reduce response bias which are excluded from scoring and factor analysis (see S1 Appendix: S1 Table for further details regarding scale development).

### Compassion scale

The Compassion Scale (CS; [33,41]) assesses compassion for others, based on Neff’s theoretical model of self-compassion [3,42]. Neff’s conceptualization of self-compassion emphasizes affective and cognitive processes, and involves six components presented as three bipolar continuums: self-kindness and self-judgment; mindfulness and overidentification; common humanity and isolation. Taken together, these six components interact to capture both compassionate (self-kindness, mindfulness, and common humanity) and uncompassionate (self-judgment, overidentification, and isolation) self-responding. This model provides the basis of the most commonly used measure in self-compassion research, the Self-Compassion Scale (SCS; [42]).

While the SCS is widely used, there is debate regarding the inclusion of the negative subscales (capturing uncompassionate self-responding) in a total score. Researchers argue that the positive and negative elements of the scale reflect two distinct factors, which have been shown to have differing effects on quality of life and well-being, and propose using the scores for the positive and negative items separately [12,25,43]. Neuroimaging studies further support the theoretical and statistical arguments that the SCS may be measuring two distinct processes [44], and some state that it is beneficial to study compassionate and uncompassionate self-responding separately to better understand compassion as a protective factor in psychopathology [45,46]. However, Neff supports the use of a total score or six subscale scores rather than separating the positive and negative subscales, arguing that it is common for opposite ends of bipolar continuums to differentially predict outcomes [26,47–49]. There is empirical support for the use of a total score, as well as two scores separating positive and negative items, based on the six-factor theoretical model.

In developing the CS, the authors adapted Neff’s model of self-compassion to be relevant to other-focused attitudes, focusing on emotional responding, cognitive understanding, and attention. The positive elements (kindness, mindfulness, and common humanity) remained similar, while the negative elements were adapted and collapsed into a single factor labelled “indifference.” The final scale has four factors with a total of 16 items (see S1 Appendix: S1 Table for further details regarding scale development).

### Sussex-Oxford compassion scales

The Sussex-Oxford Compassion Scales (SOCS; [34]) assess compassion for self (SOCS-S) and compassion for others (SOCS-O), using a five-factor structure based on a theoretical model proposed by Strauss et al. [24]. In response to the lack of consensus on a definition of compassion, Strauss and colleagues conducted a qualitative review in an effort to consolidate a range of different conceptualizations and generate an operational definition that includes cognitive, affective, and behavioural dimensions. Based on the qualitative review, the authors propose a definition of compassion consisting of five elements: recognizing suffering; understanding the universality of human suffering; feeling for the person suffering; tolerating uncomfortable feelings; and motivation to act/acting to alleviate suffering. They then conducted a systematic review of self- and observer-rated measures of compassion and rated the extent to which measures assessed each of the identified five elements. This five-element model was later empirically tested with a series of factor analytic studies with existing compassion measures, which supported the five-factor structure, however also identified limitations with existing measures and identified a need for the development of new measures [50]—thus, the SOCS were developed.

Items were generated through interviewing experts in contemplative approaches (*N* = 22, 72.7% women)—researchers defined experts as researchers or teachers in the fields of mindfulness and compassion, and included at least one researcher and one teacher from each of the six continents. Experts generated 155 items assessing compassion for others, and 101 items assessing self-compassion, which were then reduced to 120 total items (60 other, 60 self) through iterative review. Items were subsequently reviewed by 15 experts and 15 nonexperts (undergraduate students, 60% women), who were asked whether the items represented the element for which they were intended. Item reduction resulted in 57 other-compassion and 58 self-compassion items. The next stage involved further item reduction using CFA with 1,017 health care workers (79.6% women), in which the four highest loading items for each of the five proposed factors were retained, resulting in 20 items assessing compassion for others, and 20 items assessing self-compassion (see S1 Appendix: S1 Table for further details regarding scale development).

### Compassion and other psychological constructs

The relationships between compassion and other psychological constructs inform a key aspect of the development of each compassion measure. In assessing convergent validity, authors for each scale selected measures based on literature demonstrating relationships between compassion (for self, for others, and from others) and constructs related to self-/other-relating as well as mood and well-being. One key observation is that the relationship between compassion for self (or self-compassion) and compassion for others is unclear, and often studies examining these constructs have found only weak or insignificant correlations [13,51]. This finding was consistent for the studies developing the CEAS and CS, whereas results from the SOCS found that the SOCS-S and SOCS-O showed medium associations, which emphasizes the importance of advancing our understanding of the relationship between the different orientations of compassion. The Self-Compassion Scale [42] was included in all three studies. Gilbert et al. [32] used two scores, separating positive and negative items, while Pommier et al. [33] and Gu et al. [34] used a total score.

The CEAS and SOCS included the DASS-21 [52,53], assessing depression, anxiety, and stress, and the Warwick-Edinburgh Mental Well-Being Scale [54], assessing well-being. The relationship between self-compassion and symptoms of psychopathology is well documented, suggesting that self-compassion is associated with lower levels of psychopathology [20,55] and enhanced well-being [17,56,57]. The relationship between compassion for others and these constructs is less clear, and thus examining correlations with new measures of compassion for others presents an opportunity to advance understanding in this area [13,58]. The authors of the SOCS included the Five Facet Mindfulness Questionnaire [59], excluding the “observing” items. Mindfulness and compassion are commonly discussed in tandem, and while authors emphasize the importance of distinguishing between these different processes [2,60,61], mindfulness is a proposed factor in the SCS and CS. Thus, we would anticipate that measures of compassion based on models that explicitly include mindfulness would be positively associated with a measure assessing this separate but overlapping construct. The authors of the CS included a measure of attachment to assess discriminant validity, as attachment represents a way of measuring a self-other schema that is related but distinct from compassion for others [33]. Similarly, self-compassion has been demonstrated to be related to attachment style [18,62–65], which can be theoretically understood from an evolutionary perspective—individuals develop the capacity for caring motives through early experiences with caregivers, influencing neurobiology and subsequent ways of relating to self and others.

### Objectives

The objectives of the current study are to translate each of these measures from the original English to French, analyze the factor structure proposed by each respective theoretical model, and examine the relationship between compassion (to self, to others, and from others) and other related constructs. The ultimate goal is to expand the use of these measures to French-speaking populations, thereby contributing to a more complete understanding of compassion that accounts for multicultural and linguistic diversity. The importance of having valid and reliable measures in more than one language is key to advancing psychological research beyond English-speaking contexts, and is especially important when conducting studies and ensuring accessibility in multilingual contexts.

## Materials and methods

### Translation

The English versions of the CEAS, CS and SOCS were translated to French using a five-stage process [66–68], with permission from the authors of the original English scales. In stage one (initial translation*)*, items of all scales were forward-translated (English to French) independently by two bilingual native-French speakers (one translator familiar with the scale content, and one who was naïve to the scale objective). During stage two (synthesis), discrepancies between translations were discussed between the two translators, and resolved with the assistance of a third native-French-speaking research assistant. In stage three (back translation), the synthesized translations were translated from French back to English by two bilingual native-English speakers. In stage four (expert committee), a committee consisting of the principal investigator, co-investigators, and translators met to consolidate all versions of the scale and produce a pre-final version. Decisions were documented in translation reports, which were shared along with the pre-final version of the translated scale with authors of the English scales for feedback and to ensure scale equivalency (see S2 Appendix: Translation Reports). Time constraints prevented such feedback regarding the pre-final French version of the CS.

During the final stage (testing), the pre-final scales were tested with a small sample of native-French speakers (*n* = 7) to provide feedback regarding the test-takers’ experience and understanding of the scale items. Test-takers were asked to complete all translated scales online via the survey platform, *Qualtrics*. They had not previously reviewed the English scales. Upon completion, they were offered the option to provide feedback either verbally via a phone conversation with the PI, or in written form via email. All individuals chose to speak with the PI. Questions/prompts included, “please describe your experience completing the questionnaires,” “was there anything that was unclear,” and “do you have any comments on the phrasing of the items?” Overall, feedback was positive (i.e., the questionnaires were clear and easy to understand) without any proposed changes to the phrasing of the items. However, participants shared that they experienced reading the introductory sections for the CEAS scales as repetitive, as background information with details defining compassionate engagement and action is repeated for each of the three scales. Based on this feedback, and with permission from the authors of the English CEAS, the full directions for the CEAS appeared only in the first scale (CEAS-SC), and were shortened for the subsequent scales (CEAS-TO and CEAS-FROM; see S2 Appendix: Translation Reports for details regarding these edits).

### Participants and recruitment

Ethics approval was obtained from McGill University’s Research Ethics Board (REB File Number: 19-11-034). Participants were recruited via online advertising distributed via university listservs and social media platforms. Inclusion criteria included identifying French as one’s primary or native language and being 18 years or older. Participants provided informed written consent prior to completing an online survey using the platform *Qualtrics*. Recruitment and data collection took place from January 2 to July 29, 2021. Determining adequate sample size was informed by recommendations that a minimum sample should consist of 10 observations per scale item [69–72], as well as guidance that most models with fewer than 40 items can be tested with a minimum sample of 200 participants [73–75]. Comrey and Lee [76] provide classifications based on absolute sample size, rating a sample of 100 as poor, 200 as fair, 300 as good, 500 as very good, and 1,000 as excellent. Given that the longest scale in the present study consists of 20 items (SOCS-S/SOCS-O), a minimum sample of 200 participants is required.

## Measures

The following three measures were translated for the purposes of validation in this study.

### Compassionate Engagement and Action Scales (CEAS)

The CEAS [32] consist of three scales, each with 13 items assessing (1) self-compassion (CEAS-SC), (2) compassion to others (CEAS-TO), and (3) compassion from others (CEAS-FROM), i.e., the ability to receive compassion from important people in an individual’s life. On each scale, the individual is asked to rate how a statement applies to themselves when they or the people in their lives are distressed. Responses are reported using a scale of 1 (*never*) to 10 (*always*).

#### Compassion Scale (CS)

The CS [33] is a 16-item self-report measure assessing compassion towards others, using four subscales: kindness, mindfulness, common humanity, and indifference (reverse-scored). Participants are asked to indicate how often they feel or behave in a certain manner on a scale of 1 (*almost never*) to 5 (*almost always*).

#### Sussex-Oxford Compassion Scales for Self (SOCS-S) and Others (SOCS-O)

The SOCS [34] consists of two scales, each with 20 items assessing (1) compassion for self, and (2) compassion for others. Each scale is comprised of five dimensions: recognizing suffering, understanding the universality of suffering, feeling for the person suffering, tolerating uncomfortable feelings, and motivation to act/alleviate suffering. For each scale, participants are asked to indicate how true each statement is for them, on a scale of 1 (*not at all true*) to 5 (*always true*).

The following measures were used to assess convergent validity. Measures were selected based on theoretical relationships between the identified constructs with compassion for self and others. Where possible, the same measures used in the English development studies were used to assess the convergent validity of the French translations (depending on the availability of validated French versions of these measures).

#### Depression, Anxiety, and Stress Scale– 21 (DASS-21)

The DASS-21 [53] is a short version (21 items) of a 42-item self-report questionnaire assessing three related negative emotional states: depression, anxiety, and tension/stress. Individuals are asked to rate how often each item has applied to them in the past week on a scale of 0 (*did not apply to me at all*) to 3 (*applied to me very much or most of the time*). The DASS and DASS-21 were translated to French by Martin [77], available via open access on the DASS website [78].

#### Experiences in Close Relationships–Revised (ECR-R)

The ECR-R [79] is a 36-item self-report measure used to assess two dimensions of attachment: anxiety and avoidance. Individuals are asked to rate how they generally experience relationships on a scale of 1 (*strongly disagree*) to 7 (*strongly agree*). The present study used a 12-item short-form, translated and validated by Favez et al. [80].

#### Five Facet Mindfulness Questionnaire–Short Form (FFMQ-SF)

The FFMQ-SF [59] is the short version (24-item) of the 39-item self-report measure used to assess five facets of mindfulness: observation, description, aware actions, non-judgmental inner experience, and non-reactivity. Individuals are asked to rate a series of statements about thoughts, experiences, and actions in daily life on a scale of 1 (*never or very rarely true*) to 5 (*very often or always true*). The full-length FFMQ was translated to French by Heeren et al. [81], and a short-form was validated by Herrera et al. [82].

#### Self-Compassion Scale (SCS)

The SCS [42] is a 26-item self-report measure used to assess self-compassion, based on six subscales assessing three positive dimensions of compassionate self-responding (self-kindness, mindfulness, common humanity) and three negative dimensions of uncompassionate self-responding (self-judgment, overidentification, isolation). Participants are asked to rate how often they have behaved in a certain manner on a scale of 1 (*almost never*) to 5 (*almost always*). The scale can be scored using a six-factor model using each of the six identified subscales or a total score [26,42], or using a two-factor model assessing compassionate self-responding (SCS-Positive) and uncompassionate self-responding (SCS-Negative; [43,83–85])—the present study uses the latter scoring method. Psychometric properties of the French translation of the SCS are supported by Kotsou and Leys [86].

#### Warwick-Edinburgh Mental Well-Being Scale (WEMWBS)

The WEMWBS [54] is a 14-item self-report measure used to assess mental well-being. Individuals are asked to rate how often they have experienced specified statements or thoughts on a scale of 1 (*none of the time*) to 5 (*all of the time*). The French translation was validated by Trousselard et al. [87].

### Analyses

All statistical analyses were performed on R 4.2.1. The analysis script and dataset are available via the Zenodo Repository [88,89].

#### Confirmatory factor analysis

Confirmatory Factor Analysis (CFA) was conducted to examine the factor structure of each of the translated measures (CEAS, CS, SOCS). The rationale guiding each CFA was to attempt to replicate the models proposed by the development/validation studies of the English scales. In each case, CFA was used to test a one-factor model to assess the appropriateness of a total score, and the models proposed by the respective scale development studies (multi-dimensional, hierarchical models). A non-hierarchical model was fit to assess the separate components theoretically proposed for each scale. All CFAs were fit with lavaan version 0.6–12 [90,91]. semTools 0.5–6 [92] was used to visualize path diagrams. Before fitting any model, each scale’s polychoric correlation matrix was examined using psych R package version 2.1.9, suitable for use with ordinal data [93]. Spearman correlation was used for the CEAS based on the number of response options in the Likert-type scale for this measure—as the number of levels for each item increases, the distribution of scores more closely approximates a numeric datatype [93]. All scales examined had positive semi-definite polychoric matrices.

Models for the CEAS and SOCS were fit with the robust maximum likelihood (MLR) estimator, and models for the CS were fit with the weighted least squares mean- and variance-adjusted estimator (WLSMV), which replicates methods used in the English validation studies for each measure (for an expanded discussion regarding the use of MLR versus WLSMV, please see S1 Appendix: Model Estimators). All models had standardized latent variables. Cases with complete responses for a given scale were included.

The following fit indices were used to indicate model-data fit: comparative fit index (CFI; [94]), non-normed fit index (NNFI; [95]), root mean square error of approximation (RMSEA; [96]), standardized root mean square residual (SRMR; [97]), and Akaike information criterion (AIC; [98]). The chi-square (χ^2^) test of model fit was reported, however due to the hypersensitivity of this statistic (e.g., to sample size), significance-level was not used to indicate model fit [34,70]. Goodness of fit was assessed following recommendations laid out by Williams et al. [99] for both liberal and conservative cutoff points: CFI and NNFI should be close to or greater than 0.90 (liberal) or 0.95 (conservative); RMSEA should be 0.10 or less (liberal) or 0.06 or less (conservative); and SRMR should be 0.10 (liberal) or 0.05 (conservative). Factor intercorrelations and loadings were also considered when assessing model fit. The AIC was then used to compare fit of the proposed models, with lower AIC values indicating improved fit. Differences in AIC scores were assessed based on criteria set out by Burnham & Anderson [100]. Note that AIC cannot be calculated while using the WLSMV estimator, and thus was not included for the CS. These guidelines are consistent with the reporting used by Gu et al. [34] in the development of the SOCS.

#### Internal consistency and floor/ceiling effects

Internal consistency of total scale and subscales was assessed using both Cronbach’s alpha and omega coefficients, given that assumptions of Cronbach’s alpha are often violated (e.g., Cronbach’s alpha assumes that scale items are continuous and normally distributed; [101]). Values greater than or equal to 0.70 indicate good internal consistency [102,103], with values greater than 0.64 considered adequate [102]. Omega and alpha coefficients were computed using the compRelSEM function in semTools on the model that reflects the model with the most parameters in the previously published development articles.

Floor and ceiling effects were examined for each item by scaling scores into a range between 0 and 1, and averaging all item responses. The value for each item should not be less than 0.05 or greater than 0.95.

#### Measurement invariance

Multi-group CFA (semTools; [92]) was used to assess measurement invariance based on age classification, using the median age (30 years) to split the sample into two groups. Three levels of measurement invariance were tested sequentially, representing progressively more stringent levels of equivalence [104]: (1) configural invariance: assessing whether the underlying factor structure remains constant across groups by fitting the basic model without constraints; (2) metric invariance: assessing whether measurement units are equivalent, factor loadings are fixed to be equal across groups while intercepts may vary; and, (3) scalar invariance: assessing whether item intercepts have equal meaning across groups, constraining factor loadings and intercepts to be equal. Goodness of fit for each invariance test was assessed using CFI and RMSEA, based on the same cut-off criteria used for CFA. Comparisons between invariance tests were made using criteria laid out in the Dutch translation of the SOCS, whereby changes in CFI values should be < 0.01, and changes in RMSEA values should be < 0.015 [105]. In each case, the hierarchical model proposed by the English scales was tested, and if the model did not converge, a non-hierarchical model was assessed.

#### Convergent validity

Convergent validity of the total and subscale scores for the translated measures was assessed through Pearson correlations, using corr.test and corr.p functions from the psych R package [93]. All values were adjusted using the Benjamini-Hochberg method [106], a statistical procedure which reduces the false discovery rate to prevent Type I errors. Correlations of the translated measures were conducted with related constructs using previously described measures assessing self-compassion (SCS), psychological distress (specifically, depression, anxiety, and stress; DASS-21), attachment (ECR-R), mindfulness (FFMQ), and mental well-being (WEMWBS). The strength of the bivariate correlations was interpreted following Cohen’s effect sizes [107], where *r* ≤ 0.10 indicates small, *r* = 0.30 indicates moderate, and *r* = 0.50 indicates large magnitude. Based on the literature examining relationships between compassion for self and others with other psychological constructs, we predict that the translated measures will be positively associated with the positive subscales of the Self-Compassion Scale, as well as measures assessing mindfulness and mental well-being, and negatively associated with the negative subscales of the Self-Compassion Scale, and measures assessing psychological distress and attachment insecurity.

## Results

### Demographics

See Table 1 for complete demographic information. Participants ranged in age from 18 to 72 years (median age = 30 years), and the majority identified as women. In terms of ethnicity, participants were asked to enter a text response, and the majority identified as White. Most participants reported learning French in Quebec, followed by France.

**Table 1:** Demographic information.

Variable (N)	Mean (SD), Range
Age (317)	34.97 (13.64), 18–72
Number of Languages Spoken (316)	2.22 (0.82), 1–5
	***N* (%)**
Gender (318)	
Woman	276 (86.79)
Man	36 (11.32)
Non-Binary	6 (1.89)
Ethnicity (313)	
White	285 (91.05)
Other	28 (8.95)
Education Level (298)	
Certificate or Diploma (e.g., college diploma including CEGEP, technical school)	130 (43.62)
Bachelor’s Degree	89 (29.87)
Master’s Degree	60 (20.13)
Doctorate and Post Doctorate	14 (4.70)
Diploma in Medicine, Dental Medicine, Veterinary medicine	3 (1.01)
No diploma or certificate	2 (0.67)
Location where French was learned	
Quebec	235 (78.33)
France	45 (15.00)
Africa	7 (2.33)
Canada (outside Quebec)	4 (1.33)
Other	9 (3.00)

*Note*. Number of participants varied for each CFA (ranging from *N* = 311 to *N* = 384), as cases with complete responses for a given scale were included—more participants completed questionnaires that were placed earlier in the online survey (SOCS) than later in the survey (CEAS, demographics).

### Confirmatory factor analysis

#### Compassionate engagement and action scales

Fit indices for all models are shown in Table 2. Path diagrams are available in supporting information (S1-S9 Figs in S2 Appendix). Fit indices for one-factor and two-factor models for the CEAS-SC, assessing self-compassion, showed poor fit. A three-factor, non-hierarchical model showed acceptable fit across fit indices, assessing sensitivity to suffering, engagement with suffering, and compassionate action. The three-factor, three-level hierarchical model proposed by the scale authors, in which sensitivity to suffering and engagement with suffering load onto “compassionate engagement,” did not converge on a solution. As such, the three-factor, non-hierarchical model can be interpreted as best fitting the data.

**Table 2:** Model fit indices for the compassionate engagement and action scales.

Scale	Model	*N*	CFI	RMSEA [90% C.I.]	NNFI	SRMR	χ² (df)	AIC
CEAS-SC	One-Factor	332	0.89	0.13 [0.11, 0.15]	0.86	0.08	228 (35)	12899
	Two-Factor	332	0.92	0.11 [0.1, 0.13]	0.89	0.07	179 (34)	12852
	Two-Factor Hierarchical	332	0.92	0.12 [0.1, 0.13]	0.88	0.07	179 (33)	12854
	Three-Factor	332	0.94	0.10 [0.08, 0.11]	0.92	0.05	132 (32)	12809
CEAS-TO	One-Factor	323	0.89	0.13 [0.11, 0.14]	0.86	0.06	217 (35)	10927
	Two-Factor	323	0.93	0.11 [0.09, 0.12]	0.90	0.05	158 (34)	10870
CEAS-FROM	One-Factor	311	0.95	0.12 [0.1, 0.14]	0.93	0.03	188 (35)	11215
	Two-Factor	311	0.96	0.10 [0.08, 0.12]	0.95	0.03	138 (34)	11167
	Two-Factor Hierarchical	311	0.96	0.10 [0.08, 0.12]	0.95	0.03	138 (33)	11169

*Note*. Compassionate Engagement and Action Scale–Self-Compassion (CEAS-SC); Compassionate Engagement and Action Scale–Compassion to Others (CEAS-TO); Compassionate Engagement and Action Scales–Compassion from Others (CEAS-FROM). A three-factor, three-level hierarchical model for CEAS-SC did not converge on a solution. A two-factor hierarchical model for CEAS-TO was not identified.

For compassion to others (CEAS-TO), fit indices for the one-factor model suggest poor fit. The two-factor, non-hierarchical model shows acceptable fit based on the NNFI, CFI, and SRMR, while RMSEA suggests poor fit. The scale authors proposed a two-factor hierarchical model, however this model’s variance-covariance matrix was not positive definite, suggesting that the model is not identified. The two-factor, non-hierarchical model can be interpreted as best fitting the data, assessing compassionate engagement and compassionate action.

For compassion from others (CEAS-FROM), fit indices for the one-factor model show acceptable fit, with the exception of RMSEA. Fit indices for the two-factor model show acceptable fit, as do fit indices for the two-factor, hierarchical model.

#### Compassion scale

Table 3 shows fit indices for all models. Path diagrams are available in supporting information (S10-S12 Figs in S2 Appendix). All models (one-factor, four-factor, and four-factor hierarchical) show acceptable fit across fit indices. The four-factor and four-factor hierarchical models both show acceptable fit using conservative cutoffs. RMSEA and SRMR values for the four-factor model appear slightly lower, suggesting a four-factor model best fits the data; however, differences are small. The four-factor hierarchical model proposed by the scale authors is supported by fit indices in the present sample.

**Table 3:** Fit indices for the compassion scale.

Scale	Model	*N*	CFI	RMSEA [90% C.I.]	NNFI	SRMR	χ² (df)
CS	One-Factor	369	0.94	0.09 [0.08, 0.101	0.93	0.09	419 (104)
	Four-Factor	369	0.99	0.03 [0.02, 0.05]	0.99	0.05	138 (98)
	Four-Factor Hierarchical	369	0.99	0.04 [0.02, 0.05]	0.99	0.06	148 (100)

#### Sussex-Oxford compassion scales

Table 4 shows fit indices for all models. Path diagrams are provided in supporting information (S13-S18 Figs in S2 Appendix). For the Sussex Oxford Compassion Scale for Self (SOCS-S), fit indices for a one-factor model suggest poor fit. A five-factor, non-hierarchical model is supported by CFI, RMSEA, and SRMR, which all suggest acceptable fit, while NNFI suggests poor fit. Similarly, a five-factor hierarchical model appears acceptable based on CFI, RMSEA, and SRMR, with NNFI failing to meet the cutoff criteria. Both the five-factor and five-factor hierarchical models are supported in this sample.

**Table 4:** Fit Indices for the Sussex-Oxford compassion scales.

Scale	Model	*N*	CFI	RMSEA [90% C.I.]	NNFI	SRMR	χ² (df)	AIC
SOCS-S	One-Factor	384	0.67	0.15 [0.14, 0.15]	0.63	0.12	1597 (170)	1.59e+04
	Five-Factor	384	0.91	0.08 [0.07, 0.09]	0.89	0.07	548 (160)	1.49e+04
	Five-Factor Hierarchical	384	0.90	0.08 [0.07, 0.09]	0.89	0.08	574 (165)	1.49e+04
SOCS-O	One-Factor	383	0.66	0.15 [0.14, 0.16]	0.62	0.11	1612 (170)	12392
	Four-Factor	383	0.94	0.06 [0.06, 0.07]	0.93	0.05	425 (164)	11217
	Five-Factor Hierarchical	383	0.94	0.06 [0.06, 0.07]	0.93	0.05	430 (165)	11220

*Note*. Sussex-Oxford Compassion Scale–Compassion for Self (SOCS-S); Sussex-Oxford Compassion Scale–Compassion for Others (SOCS-O). A five-factor model for SOCS-O did not converge on a solution.

For the Sussex-Oxford Compassion Scale for Others (SOCS-O), fit indices for the one-factor model all suggest poor fit. The five-factor non-hierarchical model failed to converge on a solution. The five-factor hierarchical model appeared acceptable across fit indices, successfully replicating the model proposed by the scale authors.

### Internal consistency

See Table 5 for descriptive statistics, Cronbach’s alpha, and omega total estimate. Omega total estimates were calculated using standardized item loadings from two-factor hierarchical models for CEAS-TO and CEAS-FROM, and a three-factor hierarchical model for CEAS-SC. Omega total estimates for all subscales and total scores exceeded the 0.70 cutoff other than “sensitivity to suffering,” a sub-factor of compassionate engagement in the CEAS-SC.

**Table 5:** Descriptive statistics and internal consistency.

Scale	M (SD)	Min., Max.	Cronbach’s alpha	Omega Total Estimate
CEAS-SC	66.10 (14.62)	18, 97	0.87	0.90
Engagement 1 (Sensitivity to suffering)	14.60 (3.37)	4, 20	0.54	0.60
Engagement 2 (Engagement with suffering)	24.86 (6.48)	5, 39	0.72	0.73
Action	26.57 (7.62)	4, 40	0.91	0.92
CEAS-TO	80.22 (12.10)	20, 100	0.90	0.90
Engagement	47.27 (7.85)	12, 60	0.82	0.81
Action	32.96 (5.04)	8, 40	0.88	0.89
CEAS-FROM	61.43 (19.40)	10, 100	0.96	0.95
Engagement	35.52 (11.66)	6, 60	0.91	0.92
Action	25.91 (8.27)	4, 40	0.94	0.94
CS	4.29 (0.44)	1.69, 5	0.85	0.88
Kindness	4.30 (0.60)	1.75, 5	0.79	0.79
Mindfulness	4.30 (0.53)	1.5, 5	0.68	0.69
Common Humanity	4.60 (0.49)	2, 5	0.75	0.74
Indifference (Reverse)	3.98 (0.68)	1.5, 5	0.64	0.63
SOCS-S	72.60 (10.41)	43, 100	0.92	.89
Acting or motivation to act to alleviate suffering	13.91 (2.92)	4, 20	0.88	0.88
Feeling suffering	12.87 (2.85)	5, 20	0.81	0.87
Recognizing Suffering	15.41 (2.58)	8, 20	0.82	0.83
Tolerating uncomfortable feelings	11.93 (3.08)	4, 20	0.82	0.83
Understanding the universality of suffering	18.45 (2.08)	6, 20	0.82	0.82
SOCS-O	84.47 (8.25)	37, 100	0.92	0.92
Acting or motivation to act to alleviate suffering	16.75 (2.31)	6, 20	0.87	0.87
Feeling suffering	17.30 (1.98)	8, 20	0.77	0.75
Recognizing Suffering	15.58 (2.34)	8, 20	0.89	0.89
Tolerating uncomfortable feelings	16.13 (2.12)	7, 20	0.67	0.67
Understanding the universality of suffering	18.76 (1.86)	8, 20	0.87	0.87
DASS-21	20.78 (14.12)	0, 61	0.95	0.95
ECR-Anx	25.92 (9.52)	6, 42	0.90	0.90
ECR-Avoid	18.95 (7.51)	6, 42	0.84	0.83
FFMQ	78.25 (13.41)	41, 111	0.90	0.89
SCS-POS	3.14 (0.73)	1.15, 5	0.91	0.92
SCS-NEG	3.43 (0.89)	1, 5	0.93	0.94
WEMWBS	49.52 (7.83)	22, 70	0.90	0.90

*Note*. Compassionate Engagement and Action Scale–Self-Compassion (CEAS-SC); Compassionate Engagement and Action Scale–Compassion to Others (CEAS-TO); Compassionate Engagement and Action Scales–Compassion from Others (CEAS-FROM); Compassion Scale (CS); Sussex-Oxford Compassion Scale–Compassion for Self (SOCS-S); Sussex-Oxford Compassion Scale–Compassion for Others (SOCS-O); Self-Compassion Scale–Positive Subscales (SCS-POS); Self-Compassion Scale–Negative Subscales (SCS-NEG); Depression, Anxiety, and Stress Scale– 21 (DASS-21); Experiences in Close Relationships-Revised Anxiety subscale (ECR-Anx); Experiences in Close Relationships-Revised Avoidance subscale (ECR-Avoid); Five Facet Mindfulness Questionnaire (FFMQ); Warwick Edinburgh Mental Wellbeing Scale (WEMWBS).

For the CS, omega total estimates were calculated using standardized item loadings from the four-factor hierarchical model, and showed overall good internal consistency. Cronbach’s alpha ranged from 0.64 to 0.85. The only subscale that did not exceed the 0.70 cutoff for both Cronbach’s alpha and omega total estimate was the “*Indifference*” (reverse-scored) subscale.

For both SOCS-S and SOCS-O, omega total estimates were calculated using standardized item loadings from five-factor hierarchical models. Omega total estimates showed good internal consistency. The only score below the 0.70 cutoff was “*Tolerating uncomfortable feelings*,” a subscale of the SOCS-O.

There were no floor or ceiling effects (see S1 Appendix: S2 Table).

### Measurement invariance

Table 6 shows results from multi-group analyses to assess measurement invariance. Results for the CEAS-SC support measurement invariance for the three-factor model: CFI and RMSEA met the cut-off criteria. Results for CEAS-TO and CEAS-FROM supported configural, metric, and scalar invariance for the two-factor hierarchical models.

**Table 6:** Measurement invariance analyses based on age group.

Measure	Model	Invariance	CFI	Δ CFI	RMSEA	Δ RMSEA	χ^2^(df)
**CEAS-SC**	Three-factor	Configural	0.91	-	0.10	-	186.89 (64)
		Metric	0.90	0.01	0.10	0.00	205.71** (71)
		Scalar	0.90	0.00	0.10	0.00	212.19 (78)
	Two-factor hierarchical	Configural	0.89	-	0.12	-	234.69 (66)
		Metric	0.88	0.00	0.11	0.00	250.46* (75)
		Scalar	0.88	0.02	0.11	0.00	260.54 (82)
**CEAS-TO**	Two-factor hierarchical	Configural	0.92	-	0.10	-	200.13 (66)
		Metric	0.93	0.00	0.09	0.01	204.84 (75)
		Scalar	0.92	0.00	0.09	0.00	213.47 (82)
**CEAS-FROM**	Two-factor hierarchical	Configural	0.95	-	0.10	-	210.35 (66)
		Metric	0.95	0.01	0.10	0.00	230.26** (75)
		Scalar	0.95	0.00	0.10	0.00	241.51 (82)
**CS**	Four-factor	Configural	0.94	-	0.06	-	306.56 (196)
		Metric	0.94	0.00	0.06	0.00	321.30 (208)
		Scalar	0.92	0.02	0.06	0.01	357.65***(220)

*Note*. * p < 0.*05*

** *p < 0.01*

*** *p < 0.001*.

Analyses for measurement invariance for the CS for the four-factor hierarchical model indicated potential model misspecification, and results could not be reported. Invariance analyses for the four-factor non-hierarchical model showed good fit indices and comparison indices for CFI, while RMSEA failed to meet the cut-off. As such, the CS is not considered invariant across age groups.

Similarly, invariance analyses for the SOCS-S and SOCS-O indicated possible issues of misspecification, and these scales are not considered invariant across age groups.

Chi-square (χ^2^) tests are reported for descriptive purposes, but were not interpreted due to sensitivity to sample size. Invariance analyses using alternate demographic variables (e.g., gender, education level) were not possible due to insufficient sample size.

### Convergent validity

#### Compassionate engagement and action scales

Correlation coefficients between total scale scores are shown in Table 7 (see S1 Appendix: S3 Table for an expanded correlation matrix including subscale scores). The CEAS-SC was found to have a moderate positive correlation with compassion from others (CEAS-FROM), and large positive correlations with other measures assessing self-compassion (SOCS-S, SCS-POS), mindfulness (FFMQ), and mental well-being (WEMWBS). Positive correlations with a small effect size were found between the CEAS-SC and measures assessing compassion for others (CEAS-TO, CS, SOCS-O). Moderate, negative correlations were found with attachment insecurity (ECR-Anx, ECR-Avoid), and large negative correlation with psychological distress (DASS-21) and uncompassionate self-responding (SCS-NEG).

**Table 7:** Pearson correlation coefficients adjusted by Benjamini-Hochberg procedure.

Scale	1	2	3	4	5	6
1. CEAS_SC	–					
2. CEAS_TO	0.26***	–				
3. CEAS_FROM	0.36***	0.22***	–			
4. CS	0.24***	0.78***	0.13*	–		
5. SOCS-S	0.78***	0.22***	0.34***	0.26***	–	
6. SOCS-O	0.24***	0.76***	0.19***	0.77***	0.28***	–
7. SCS-POS	0.77***	0.09	0.28***	0.15**	0.72***	0.14*
8. SCS-NEG	-0.60***	-0.05	-0.30***	-0.06	-0.59***	-0.08
9. DASS-21	-0.55***	-0.01	-0.39***	-0.02	-0.53***	-0.06
10. ECR-Anx	-0.30***	0.02	-0.13*	0.05	-0.33***	-0.02
11. ECR-Avoid	-0.28***	-0.16**	-0.41***	-0.13*	-0.35***	-0.16**
12. FFMQ	0.70***	0.13*	0.21***	0.17**	0.70***	0.17**
13. WEMWBS	0.57***	0.21***	0.39***	0.21***	0.61***	0.24***

*Note*. Compassionate Engagement and Action Scale–Self-Compassion (CEAS-SC); Compassionate Engagement and Action Scale–Compassion to Others (CEAS-TO); Compassionate Engagement and Action Scales–Compassion from Others (CEAS-FROM); Compassion Scale (CS); Sussex-Oxford Compassion Scale–Compassion for Self (SOCS-S); Sussex-Oxford Compassion Scale–Compassion for Others (SOCS-O); Self-Compassion Scale–Positive Subscales (SCS-POS); Self-Compassion Scale–Negative Subscales (SCS-NEG); Depression, Anxiety, and Stress Scale– 21 (DASS-21); Experiences in Close Relationships Anxiety Subscale (ECR-Anx); Experiences in Close Relationships Avoidance Subscale (ECR-Avoid); Five Facet Mindfulness Questionnaire (FFMQ); Warwick Edinburgh Mental Wellbeing Scale (WEMWBS).

The Benjamini-Hochberg procedure was implemented to reduce false discovery rate.

* *p < 0.05*

** *p < 0.01*

*** *p < 0.001*.

The CEAS-TO was found to have large, positive correlations with other measures assessing compassion to others (CS, SOCS-O), and a small, positive correlation with compassion from others (CEAS-FROM), self-compassion (SOCS-S), mindfulness (FFMQ), and mental well-being (WEMWBS). There was a small negative association with attachment avoidance (ECR-Avoid), but no significant correlation with attachment anxiety (ECR-Anx). The CEAS-TO did not significantly correlate with the positive or negative composite scores of the Self-Compassion Scale (SCS-POS, SCS-NEG), and did not significantly correlate with measures assessing psychological distress (DASS-21).

The CEAS-FROM was found to significantly, positively correlate with a moderate effect size with measures assessing self-compassion (CEAS-SC, SOCS-S) and mental well-being (WEMWBS). Small, significant positive correlations were found with a third measure of self-compassion (SCS-POS), as well as with compassion to others (CS, SOCS-O) and mindfulness (FFMQ). Moderate, negative correlations were found between CEAS-FROM and measures assessing uncompassionate self-responding (SCS-NEG), psychological distress (DASS-21), and attachment avoidance (ECR-Avoid), and a small negative correlation with attachment anxiety (ECR-Anx).

#### Compassion scale

Significant positive correlations with large effect size were found between the total score for the CS with measures of compassion for others (CEAS-TO; SOCS-O). Significant positive correlations with small effect size were found with self-compassion (CEAS-SC, SOCS-S, SCS-POS), mindfulness (FFMQ), and well-being (WEMWBS). A small negative correlation was found with attachment avoidance (ECR-Avoid). The CS did not significantly correlate with measures of uncompassionate self-responding (SCS-NEG), psychological distress (DASS-21), nor attachment-anxiety (ECR-Anx).

#### Sussex-Oxford compassion scales

The SOCS-S was found to have significant positive correlations with large effect size with other measures of self-compassion (CEAS-SC, SCS-POS), as well as with measures assessing mindfulness (FFMQ), and well-being (WEMWBS). Significant negative correlations with large effect size were found with measures assessing psychological distress (DASS-21) and uncompassionate self-responding (SCS-NEG). A significant negative correlation with moderate effect size was found with attachment insecurity (ECR-Anx and ECR-Avoid).

The SOCS-O showed significant positive correlations with large effect size with other measures assessing compassion for others (CEAS-TO, CS). Significant positive associations with small effect size were found between SOCS-O and self-compassion (SOCS-S, SCS-POS), mindfulness (FFMQ), and well-being (WEMWBS). The SOCS-O did not significantly correlate with measures assessing uncompassionate self-responding (SCS-NEG), psychological distress (DASS-21), nor attachment anxiety (ECR-Anx), and showed a small negative correlation with attachment avoidance (ECR-Avoid).

## Discussion

The aim of this study was to translate measures assessing compassion (for self, others, and from others) from English to French, and validate the translated measures in a French-speaking population. Using confirmatory factor analysis with the French translations, we were able to reproduce the models proposed by the authors of the original English scales for all measures except the CEAS-SC and CEAS-TO. For these two scales, the models that best fit the data were non-hierarchical, identifying the latent variables proposed by the scale authors but suggesting that these latent variables do not converge on a higher-order factor. Psychometric properties for the translated measures appeared robust.

### Factor structure

#### Compassionate engagement and action scales

In the original development article, the proposed factor structure for the CEAS-SC, assessing self-compassion, differs from the factor structure proposed for CEAS-TO and CEAS-FROM. For the CEAS-SC, “engagement” was shown to consist of two latent constructs, sensitivity to suffering and engagement with suffering [32], resulting in a three-factor, three-level hierarchical model. This model (in which sensitivity to suffering and engagement with suffering load onto “compassionate engagement”) did not converge on a solution in our sample. However, CFA results for the French translation support a three-factor non-hierarchical model, identifying the three latent constructs from the original English version of the scale.

Given that the three-factor, three-level hierarchical model is relatively more complex than a three-factor non-hierarchical model, failure to converge may be due to inadequate sample size [72]. Internal consistency for sensitivity to suffering was acceptable, and internal consistency for engagement with suffering and compassionate action were good. The three dimensions are significantly positively correlated, with moderate effect size between sensitivity to suffering and engagement with suffering, and large magnitude with compassionate action. Internal consistency for the total score is excellent, which speaks to the use of a total score. As such, we recommend using the French CEAS-SC to assess the three components described by the scale authors (sensitivity to suffering, engagement with suffering, and compassionate action) as well as a total score for self-compassion. Future research with larger samples would contribute to assessing whether the hierarchical model can be reproduced using the French translation.

Similarly, the model that best fit the French version of CEAS-TO was also non-hierarchical. However, internal consistency for the total score is excellent. The two factors show a strong positive correlation, and good internal consistency. Taken together, evidence supports the use of a total score.

The French translation of the CEAS-FROM, assessing compassion from others, appears to replicate the two-factor hierarchical structure proposed by the scale authors. Fit indices for both the two-factor and two-factor hierarchical models were acceptable. The model that best fit the data appears to be the two-factor model, with an AIC of 11167 compared to the second-order model’s AIC of 11169 (ΔAIC = 2). However, an AIC difference of this small magnitude (between 0 and 2) indicates that the models are similarly supported by the data [100]. Given the theoretical rationale, we recommend using the two-factor hierarchical model. The scale can be used to assess separate scores for compassionate engagement and compassionate action, as well as a total score for compassion from others. The two factors were significantly correlated and showed good internal consistency.

Engagement and action factors across the CEAS-SC, TO, and FROM correlate highly, which could suggest that they are conceptually overlapping or lack discriminant validity. However, given the clear theoretical rationale for examining engagement and action as separate processes, we do not propose collapsing these factors. Clinically it is also useful to examine engagement and action separately, as individuals who are more sensitive to suffering may be more sensitive to psychopathology, if heightened sensitivity and engagement are not also coupled with action [32].

Our study is not the first to find difficulties replicating the proposed factor structure for the CEAS. Researchers validating a Slovak translation had similar findings to ours, and found a bifactor ESEM model supported the use of the CEAS to assess compassionate engagement and compassionate action in CEAS-TO and CEAS-FROM, but not CEAS-SC [108]. A validation study in a UK general population sample found evidence to support two-factor models for all three orientations of compassion (to self, to others, from others; [51]), which is consistent with the models with best fit in our study for CEAS-TO and CEAS-FROM. The UK study did not test hierarchical models. An international sample of family carers found evidence supporting a two-factor hierarchical solution [109]. This study also found support for the proposed three-factor, three-level hierarchical model for CEAS-SC, but notes poor internal consistency for this model. While the sample was international, the measures were completed in English, and largely based in Australia, the United States, and the United Kingdom. Validation of a Japanese translation supports single factor structures for all three scales [110]. The Japanese translation includes 10 items per scale, and through data analysis they removed three items from the CEAS-SC and one item from the CEAS-TO due to low factor loadings. Comparatively, the French translation used in the present study is closely aligned with the theoretical model proposed by the scale authors, and provides robust evidence for use of each of the CEAS scales to assess compassionate engagement and compassionate action.

#### Compassion scale

The French translation of the Compassion Scale appears to replicate the factor structure of the original English version: four factors (kindness, mindfulness, common humanity, and lessened indifference) loading onto a second-order factor (compassion for others). The scale and subscales showed good internal consistency, except for the indifference subscale showing poorer internal consistency. The latter could indicate a measurement issue related to including both positively and negatively phrased items in a scale [111].

An Italian translation of the CS has been validated [112], which also supports the proposed factor structure for the English version.

#### Sussex oxford compassion scales

The French translation of the SOCS-S appears to replicate the five-factor hierarchical model proposed by scale authors, with adequate fit across indices other than the NNFI. These findings demonstrate that the French translation accurately captures the five factors proposed by the authors (recognizing suffering understanding the universality of suffering, feeling for the person suffering, tolerating uncomfortable feelings, and motivation to act/acting to alleviate suffering), and that the scale can be used to provide a total score representing “compassion for self.” The SOCS-S subscales showed good internal consistency.

Similarly, results from the French translation of the SOCS-O show that the translated scale appears to replicate the proposed five-factor hierarchical model. Thus, the translated scale can be used to provide a total score assessing “compassion for others” comprising of five factors which aligns with the authors’ theoretical understanding of compassion [24,34]. Interestingly, when a five-factor non-hierarchical model was fit, results showed that two of the factors (“*Tolerating uncomfortable feelings*” and “*Feeling for the person suffering*”) were almost perfectly correlated (*r* > 0.99). This high correlation suggests poor discriminant validity, and so a four-factor model was fit in which the two highly correlated factors were combined. This four-factor model appears to best fit the data in our sample based on the AIC value (ΔAIC = 3), although similarly to the CEAS-FROM, an AIC difference of this small magnitude indicates support for both models [100]. In considering this finding, we noted that participants tended to respond more favorably to the SOCS-O than the SOCS-S (the mean is higher), and it is possible that social desirability may play a factor—it is perhaps less socially acceptable to report being uncompassionate towards others than towards oneself. The smaller range in values may impact the results. Given the theoretical support for a five-factor hierarchical model, the utility of replicating the factor structure of the original English scale, and the acceptable fit indices in our sample, we propose using a five-factor hierarchical structure for the French translation. The SOCS-O subscales and total score showed good internal consistency.

Similar to our findings, a Dutch translation of the SOCS-S also found support for a five-factor model [105]. Validation of a Slovak translation found support for the proposed five-factor hierarchical structure for the SOCS-O, but advises against using the total score for the SOCS-S as they found support for two dominant factors (which they refer to as “Rational Compassion” and “Emotional/Behavioural Compassion”) above the five proposed factors [113]. Findings support the five-factor hierarchical structure for Korean [114], Italian [112], and Swedish [115] translations.

### Measurement invariance

We assessed measurement invariance for all scales across age (median split). Results showed support for measurement invariance for the CEAS-SC, CEAS-TO, and CEAS-FROM, and partial support for the CS. We were not able to assess measurement invariance for the SOCS-S or SOCS-O due to possible misspecification. Measurement invariance assesses whether a questionnaire has the same structure and meaning across groups, indicating whether scores can be compared across groups (in this case, age). Sample size posed a limitation to analyzing measurement invariance in the present study, contributing to the possible misspecification issues for the SOCS-S/O and four-factor hierarchical model of the CS. A larger sample and/or additional datasets would enable further analyses to assess whether the issues in the present study are due to sample size, or whether there are significant differences in how different demographic groups respond to the questionnaires. Future research should examine measurement invariance across additional demographic groups, including gender and education level.

### Convergent validity

Convergent validity analyses provide insight into the different orientations of compassion and their association with other constructs. Correlations between measures assessing self-compassion and other measures were consistent with predictions, and in line with the broader literature. The SOCS-S total score and the CEAS-SC were significantly correlated in the predicted direction with all other measures. These findings are consistent with existing literature supporting the association of self-compassion with higher levels of mindfulness and well-being, and lower levels of psychological distress and insecure attachment [17,18,24,116–119].

The total score for the CEAS-FROM was significantly correlated with all measures in the predicted direction. These findings suggest that individuals who are more able to receive compassion from others experience higher levels of self-compassion, compassion for others, mindfulness, and well-being, and lower levels of psychological distress, attachment insecurity, and uncompassionate self-responding. Correlations with measures of compassion for others were small, which suggests that the ability to receive compassion from others may not be strongly related to the ability to show compassion to other people. Theoretically, this observation fits within the context of research demonstrating that individuals with insecure attachment styles may feel more comfortable showing care for others than receiving care [120,121], and that these competencies may be distinct. Results can also be understood in the context of research on fears of compassion, in which fear of receiving compassion has a stronger association with mental health outcomes than fears of being compassionate to others [37]. These findings highlight the importance of further research on the flow of compassion and understanding the relationship between different orientations of compassion.

The relationship between compassion for others and other orientations of compassion is in line with our predictions, and consistent with existing literature. Generally, all measures of compassion for others (CEAS-TO, CS, SOCS-O) were associated with one another as well as with at least two of the measures assessing self-compassion. The measures assessing compassion for others were not significantly correlated with measures assessing uncompassionate self-responding (SCS-NEG), psychological distress, nor attachment anxiety, but showed small negative correlations with attachment avoidance. Findings related to the relationship between compassion for others and mental health are markedly different from findings related to self-compassion and psychopathology, and in line with previous research [13]. The lack of relationship between compassion for others and uncompassionate self-responding (SCS-NEG) is in line with the common observation that individuals are more likely to be compassionate to others than to themselves, and could be harsh towards themselves irrespective of how they respond to others. However, the CS was not significantly correlated with SCS-NEG, which is somewhat surprising given that the originally proposed three negative subscales (later collapsed into one subscale labelled “indifference”) were adapted from the three negative subscales of the SCS. These findings could be understood in the context of social desirability bias, as when asked how one would respond to another’s suffering on a questionnaire, individuals may be more likely to report that they *would* be compassionate toward others, whereas they may be more honest about being uncompassionate to themselves on the self-compassion measures.

The relationship between compassion to others and attachment insecurity is somewhat surprising, as the way that an individual is cared for and the types of caring relationships that are modelled to them by caregivers seem like important factors associated with how individuals relate to others. While our results support a theoretical relationship with attachment avoidance, but not attachment anxiety, it is noteworthy that the subscales of the ECR-R are not considered distinct orthogonal constructs—they are conceptually overlapping [122], and thus these results should not be over-interpreted. The differing relationship between attachment anxiety and avoidance may be due to the phrasing of the scale items, as all the items in the ECR-Anxiety subscale are phrased negatively, while the majority of the items in the Avoidance subscale are phrased positively and reverse-scored. It is particularly interesting that *self-*compassion appears to have a more robust relationship with attachment insecurity, highlighting the importance of caregiving relationships in developing a compassionate style of relating to oneself.

### Implications and limitations

Findings from the present study support the use of the translated measures using the factor structures proposed by the original English studies, with the exception of the CEAS-SC and CEAS-TO, for which CFA findings for two-factor models were supported. The two-factor non-hierarchical model is still theoretically aligned with the conceptualization of “engagement” and “action” reflecting distinct processes. Internal consistency and convergent validity results support the use of a total score for all measures.

Each of the measures reflects a distinct theoretical conceptualization of compassion, and furthermore, the measures use different approaches to developing these theoretical conceptualizations. Gilbert [123] distinguishes between a stimulus-response algorithm that identifies distinct skills and competencies in two different stages (engagement and action), versus a cluster-diagnostic approach which identifies core processes that are commonly understood as related but may vary in how they group together. He identifies this as a potential limiting factor of relying on factor analysis to define a given phenomenon. The goal of the present study was to provide valid measures for use with French populations to further study compassion using these different models, and not to identify a “correct” model. The model chosen necessarily depends on the theory underpinning a given study [27]. Recent studies on compassion using alternate forms of measurement, for example physiological measures such as heart-rate variability, provide important additional data to this debate [124–129].

There are several limitations to the present study. While a thorough forward- and back-translation process was undertaken including consultation with authors of the original source scales, the pilot stage was conducted with a small group of participants and did not include structured focus groups and/or cognitive interviews. Incorporating a more thorough qualitative stage into the translation process could have strengthened the semantic equivalence of the translated scales [130]. Additionally, while our sample size surpasses the minimum criteria for factor analysis [76], issues with model fit for the CEAS-SC and CEAS-TO may be impacted by sample size due to the relative complexity of the proposed models. Sample size also negatively impacted measurement invariance analyses, limiting the demographic variables that could be used and potentially contributing to issues of misspecification for the CS and SOCS. Future studies examining measurement invariance for these scales should consider a larger sample size as well as including a comparative sample using the original scale language and culture [131]. These added steps would provide further insight into the equivalence, meaning, and interpretation of the scales in diverse cultural and linguistic contexts.

Finally, representativeness and generalizability should be considered. While participants in the present study were a convenience sample and not a representative sample of the Quebec population, representation based on ethnicity, age, and level of education are comparable to the most recent census data [132]. The average age of the population in Quebec is 42.80 years—our sample is slightly younger, at 34.97 years. Comparing ethnicity in our sample to the Quebec population, 91.05% of our sample self-identified as White compared to 83.86% of the Quebec population identifying as “not a visible minority.” Level of education attainment in the current sample also appears comparable to the Quebec population: 36.87% of the Quebec population reported holding a postsecondary diploma/certificate/degree below bachelor’s as their highest level of education, and 43.62% of our sample reported the same, while 23.54% of census participants reported holding a bachelor’s degree compared to 29.87% in our sample. The present sample predominantly identified as women (86.79%), which is not representative of the Quebec population, but is consistent with samples used for the development of the English scales. The samples involved in the development and validation of the original scales included 62% to 88% females, and 50% to 85% of participants identified as Caucasian [33] or were described as “predominantly White” in study reports where numerical information was not reported [34]. Of the 13 samples used to assess factor structure, seven samples comprised students, two comprised health care workers, three were community samples (of which two were recruited from Mechanical Turk), and one comprised meditators (see S1 Appendix: S1 Table).

Regarding generalizability, while French speakers in Canada reflect a heterogenous and culturally diverse population, there may be cultural and linguistic differences with French-speaking populations in different geographic areas. Future studies validating the use of these translations in other regions would be valuable. Lastly, participants completed an online questionnaire with a significant number of items assessing similar constructs, which may have generated response biases—for example, the tendency to be consistent.

## Conclusion

The present study has provided French translations of the Compassionate Engagement and Action Scales, Compassion Scale, and Sussex-Oxford Compassion Scales for Self and Others (see S3 Appendix), thus contributing to broadening research on compassion to include linguistically diverse populations. These additional measures contribute to broader debates regarding the operationalization and measurement of compassion, and the relationship between different orientations of compassion: for self, to others, and from others. To produce French versions of these scales, we undertook a rigorous translation process engaging both informed and naïve translators, consensus meetings with an expert committee, and consultation with original scale authors regarding final translation reports. The translation process helped to capture linguistic nuance while staying true to the meaning and theoretical underpinnings of the original scales.

Our findings support the use of these French translations as psychometrically valid questionnaires, with evidence supporting the majority of the factor structures proposed by the scale authors and recommendations to use total scores for self-compassion and compassion for others assessed with the CEAS. Future studies should further examine the factor structure of the French translations to ensure our findings are replicable.

## Supporting information

S1 Appendix Supplementary information. Model Estimators; Scale Development Summary (S1 Table in S2 Appendix); Floor and Ceiling Effects (S2 Table in S2 Appendix); Pearson Correlation Coefficients (S3 Table in S2 Appendix); Path Diagrams (S1-S18 Figs in S2 Appendix).
(DOCX)

S2 Appendix Translation reports. (DOCX)

S3 Appendix Scale items. French translations of the Compassionate Engagement and Action Scales, Compassion Scale, and Sussex-Oxford Compassion Scales for Self and Others.
(ZIP)

## References

[1] Gilbert P. The origins and nature of compassion focused therapy. Br J Clin Psychol. 2014;53: 6–41. doi: 10.1111/bjc.12043 24588760

[2] Khoury B. Compassion: Embodied and Embedded. Mindfulness. 2019;10: 2363–2374. doi: 10.1007/s12671-019-01211-w

[3] Neff K. Self-Compassion: An Alternative Conceptualization of a Healthy Attitude Toward Oneself. Self and Identity. 2003;2: 85–101. doi: 10.1080/15298860309032

[4] Shonin E, Van Gordon W, Griffiths MD. The emerging role of Buddhism in clinical psychology: Toward effective integration. Psychology of Religion and Spirituality. 2014;6: 123–137. doi: 10.1037/a0035859

[5] Gilbert P. Compassion as a social mentality: An evolutionary approach. In: Gilbert P, editor. Compassion: Concepts, Research and Applications. New York, NY: Taylor and Francis; 2017. pp. 31–68.

[6] Nussbaum M. Compassion: The Basic Social Emotion. Social Philosophy and Policy. 1996;13: 27–58. doi: 10.1017/s0265052500001515

[7] Spikins P. Prehistoric Origins: The Compassion of Far Distant Strangers. In: Gilbert P, editor. Compassion: Concepts, Research and Applications. New York, NY: Routledge; 2017.

[8] Wispe L. The Psychology of Sympathy. New York, NY: Plenum; 1991.

[9] Gilbert P, Choden. Mindful Compassion. London, UK: Constable Robinson; 2013.

[10] Lavelle BD. Compassion in Context: Tracing the Buddhist Roots of Secular, Compassion-Based Contemplative Programs. In: Seppala EM, Simon-Thomas E, Brown SL, Worline MC, Cameron CD, Doty JR, editors. The Oxford Handbook of Compassion Science. Oxford University Press; 2017. doi: 10.1093/oxfordhb/9780190464684.013.2

[11] Wang S. A conceptual framework for integrating research related to the physiology of compassion and the wisdom of Buddhist teaching. In: Gilbert P, editor. Compassion: Conceptualisations, research, and use in psychotherapy. New York, NY: Routledge; 2005. pp. 75–120.

[12] Coroiu A, Kwakkenbos L, Moran C, Thombs B, Albani C, Bourkas S, et al. Structural validation of the Self-Compassion Scale with a German general population sample. PLoS One; San Francisco. 2018;13: e0190771. doi: 10.1371/journal.pone.0190771 29408888 PMC5800544

[13] López A, Sanderman R, Ranchor AV, Schroevers MJ. Compassion for Others and Self-Compassion: Levels, Correlates, and Relationship with Psychological Well-being. Mindfulness. 2018;9: 325–331. doi: 10.1007/s12671-017-0777-z 29387268 PMC5770484

[14] López A, Sanderman R, Schroevers MJ. A Close Examination of the Relationship Between Self-Compassion and Depressive Symptoms. Mindfulness. 2018; 1–9. doi: 10.1007/s12671-018-0891-6 30294388 PMC6153895

[15] Shapira LB, Mongrain M. The benefits of self-compassion and optimism exercises for individuals vulnerable to depression. The Journal of Positive Psychology. 2010;5: 377–389. doi: 10.1080/17439760.2010.516763

[16] Yarnell LM, Neff K. Self-compassion, Interpersonal Conflict Resolutions, and Well-being. Self and Identity. 2013;12: 146–159. doi: 10.1080/15298868.2011.649545

[17] Zessin U, Dickhäuser O, Garbade S. The Relationship Between Self-Compassion and Well-Being: A Meta-Analysis. Appl Psychol Health Well-Being. 2015;7: 340–364. doi: 10.1111/aphw.12051 26311196

[18] Brophy K, Brähler E, Hinz A, Schmidt S, Körner A. The Role of Self-Compassion in the Relationship Between Attachment, Depression, and Quality of Life. Journal of Affective Disorders. 2020;260: 45–52. doi: 10.1016/j.jad.2019.08.066 31493638

[19] Körner A, Coroiu A, Copeland L, Gomez-Garibello C, Albani C, Zenger M, et al. The Role of Self-Compassion in Buffering Symptoms of Depression in the General Population. PLOS ONE. 2015;10: e0136598. doi: 10.1371/journal.pone.0136598 26430893 PMC4591980

[20] MacBeth A, Gumley A. Exploring compassion: A meta-analysis of the association between self-compassion and psychopathology. Clinical Psychology Review. 2012;32: 545–552. doi: 10.1016/j.cpr.2012.06.003 22796446

[21] Kirby JN. Compassion interventions: The programmes, the evidence, and implications for research and practice. Psychol Psychother Theory Res Pract. 2017;90: 432–455. doi: 10.1111/papt.12104 27664071

[22] Kirby JN, Tellegen CL, Steindl SR. A Meta-Analysis of Compassion-Based Interventions: Current State of Knowledge and Future Directions. Behavior Therapy. 2017;48: 778–792. doi: 10.1016/j.beth.2017.06.003 29029675

[23] Montero-Marin J, Andrés-Rodríguez L, Tops M, Luciano JV, Navarro-Gil M, Feliu-Soler A, et al. Effects of attachment-based compassion therapy (ABCT) on brain-derived neurotrophic factor and low-grade inflammation among fibromyalgia patients: A randomized controlled trial. Scientific Reports. 2019;9: 15639. doi: 10.1038/s41598-019-52260-z 31666651 PMC6821772

[24] Strauss C, Lever Taylor B, Gu J, Kuyken W, Baer R, Jones F, et al. What is compassion and how can we measure it? A review of definitions and measures. Clinical Psychology Review. 2016;47: 15–27. doi: 10.1016/j.cpr.2016.05.004 27267346

[25] Muris P, Otgaar H, Pfattheicher S. Stripping the Forest from the Rotten Trees: Compassionate Self-Responding Is a Way of Coping, but Reduced Uncompassionate Self-Responding Mainly Reflects Psychopathology. Mindfulness. 2019;10: 196–199. doi: 10.1007/s12671-018-1030-0

[26] Neff K, Tóth-Király I, Yarnell LM, Arimitsu K, Castilho P, Ghorbani N, et al. Examining the factor structure of the Self-Compassion Scale in 20 diverse samples: Support for use of a total score and six subscale scores. Psychological Assessment. 2019;31: 27–45. doi: 10.1037/pas0000629 30124303

[27] Mascaro JS, Florian MP, Ash MJ, Palmer PK, Frazier T, Condon P, et al. Ways of Knowing Compassion: How Do We Come to Know, Understand, and Measure Compassion When We See It? Frontiers in Psychology. 2020;11. Available: https://www.frontiersin.org/articles/10.3389/fpsyg.2020.547241. 33132956 10.3389/fpsyg.2020.547241 PMC7561712

[28] Montero-Marin J, Kuyken W, Crane C, Gu J, Baer R, Al-Awamleh AA, et al. Self-Compassion and Cultural Values: A Cross-Cultural Study of Self-Compassion Using a Multitrait-Multimethod (MTMM) Analytical Procedure. Front Psychol. 2018;9. doi: 10.3389/fpsyg.2018.02638 30622499 PMC6308155

[29] Wu Q, Chen C, Liang Y, Zhou N, Cao H, Du H, et al. Not Only the Forest and Trees but Also the Ground They Are Rooted in: Identifying Profiles of Self-Compassion from the Perspective of Dialecticism. Mindfulness. 2020;11: 1967–1977. doi: 10.1007/s12671-020-01406-6

[30] Lazarus RS. Emotion and Adaptation. Oxford: Oxford University Press; 1994. Available: http://public.ebookcentral.proquest.com/choice/publicfullrecord.aspx?p=4700723.

[31] Goetz JL, Keltner D, Simon-Thomas E. Compassion: An evolutionary analysis and empirical review. Psychological Bulletin. 2010;136: 351–374. doi: 10.1037/a0018807 20438142 PMC2864937

[32] Gilbert P, Catarino F, Duarte C, Matos M, Kolts R, Stubbs J, et al. The development of compassionate engagement and action scales for self and others. Journal of Compassionate Health Care. 2017;4. doi: 10.1186/s40639-017-0033-3

[33] Pommier EA, Neff K, Tóth-Király I. The Development and Validation of the Compassion Scale. Assessment. 2020;27: 21–39. doi: 10.1177/1073191119874108 31516024

[34] Gu J, Baer R, Cavanagh K, Kuyken W, Strauss C. Development and Psychometric Properties of the Sussex-Oxford Compassion Scales (SOCS). Assessment. 2020;27: 3–20. doi: 10.1177/1073191119860911 31353931 PMC6906538

[35] Gilbert P. Introducing and developing CFT functinos and competencies. In: Simos G, Gilbert P, editors. Compassion focused therapy: clinical practice and applications. Abingdon, Oxon; Routledge; 2022. pp. 243–273. doi: 10.4324/9781003035879

[36] Gilbert P. The compassionate mind: A new approach to life’s challenges. London: Constable; 2009.

[37] Kirby JN, Day J, Sagar V. The ‘Flow’ of compassion: A meta-analysis of the fears of compassion scales and psychological functioning. Clinical Psychology Review. 2019;70: 26–39. doi: 10.1016/j.cpr.2019.03.001 30884253

[38] Gilbert P, Procter S. Compassionate mind training for people with high shame and self-criticism: overview and pilot study of a group therapy approach. Clinical Psychology & Psychotherapy. 2006;13: 353–379. doi: 10.1002/cpp.507

[39] Gilbert P, Woodyatt L. An evolutionary approach to shame-based self-criticism, self-forgiveness, and compassion. In: Woodyatt L, Worthington Jr. EL, Wenzel M, Griffin BJ, editors. Handbook of the Psychology of Self-Forgiveness. New York, NY: Springer International Publishing; 2017. pp. 29–41.

[40] Lindsey S. Examining the Psychometric Properties of the Compassionate Engagement and Action Scales in the General Population. other, University of Essex. 2017. Available: http://repository.essex.ac.uk/20473/.

[41] Pommier EA. The Compassion Scale. thesis. 2010. Available: https://repositories.lib.utexas.edu/handle/2152/ETD-UT-2010-12-2213.

[42] Neff K. The Development and Validation of a Scale to Measure Self-Compassion. Self and Identity. 2003;2: 223–250. doi: 10.1080/15298860309027

[43] Brenner RE, Heath PJ, Vogel DL, Credé M. Two is more valid than one: Examining the factor structure of the Self-Compassion Scale (SCS). Journal of Counseling Psychology. 2017;64: 696–707. doi: 10.1037/cou0000211 28358523

[44] Longe O, Maratos FA, Gilbert P, Evans G, Volker F, Rockliff H, et al. Having a word with yourself: Neural correlates of self-criticism and self-reassurance. NeuroImage. 2010;49: 1849–1856. doi: 10.1016/j.neuroimage.2009.09.019 19770047

[45] Muris P, Otgaar H. The Process of Science: A Critical Evaluation of more than 15 Years of Research on Self-Compassion with the Self-Compassion Scale. Mindfulness. 2020 [cited 23 Apr 2020]. doi: 10.1007/s12671-020-01363-0

[46] Muris P, Otgaar H. Deconstructing Self-Compassion: How the Continued Use of the Total Score of the Self-Compassion Scale Hinders Studying a Protective Construct Within the Context of Psychopathology and Stress. Mindfulness. 2022;13: 1403–1409. doi: 10.1007/s12671-022-01898-4 35578653 PMC9095813

[47] Neff K. The Self-Compassion Scale is a Valid and Theoretically Coherent Measure of Self-Compassion. Mindfulness. 2016;7: 264–274. doi: 10.1007/s12671-015-0479-3

[48] Neff K. The Differential Effects Fallacy in the Study of Self-compassion: Misunderstanding the Nature of Bipolar Continuums. Mindfulness. 2022 [cited 4 Mar 2022]. doi: 10.1007/s12671-022-01832-8

[49] Neff K, Tóth‐Király I, Colosimo K. Self-compassion Is Best Measured as a Global Construct and Is Overlapping with but Distinct from Neuroticism: A Response to Pfattheicher, Geiger, Hartung, Weiss, and Schindler (2017). European Journal of Personality. 2018;0. doi: 10.1002/per.2148

[50] Gu J, Cavanagh K, Baer R, Strauss C. An empirical examination of the factor structure of compassion. PLOS ONE. 2017;12: e0172471. doi: 10.1371/journal.pone.0172471 28212391 PMC5315311

[51] Lindsey S, Hiskey S, Irons C, Andrews L. Examining the Psychometric Properties of the Compassionate Engagement and Action Scales (CEAS) in the UK General Population. OBM Integrative and Complementary Medicine. 2022;7: 1–29. doi: 10.21926/obm.icm.2203039

[52] Henry JD, Crawford JR. The short-form version of the Depression Anxiety Stress Scales (DASS-21): construct validity and normative data in a large non-clinical sample. Br J Clin Psychol. 2005;44: 227–239. doi: 10.1348/014466505X29657 16004657

[53] Lovibond SH, Lovibond PF. Manual for the Depression Anxiety & Stress Scales. 2nd ed. Sydney: Psychology Foundation; 1995.

[54] Tennant R, Hiller L, Fishwick R, Platt S, Joseph S, Weich S, et al. The Warwick-Edinburgh Mental Well-being Scale (WEMWBS): development and UK validation. Health Qual Life Outcomes. 2007;5: 1–13. doi: 10.1186/1477-7525-5-63 18042300 PMC2222612

[55] Pinto‐Gouveia J, Duarte C, Matos M, Fráguas S. The Protective Role of Self-compassion in Relation to Psychopathology Symptoms and Quality of Life in Chronic and in Cancer Patients. Clinical Psychology & Psychotherapy. 2014;21: 311–323. doi: 10.1002/cpp.1838 23526623

[56] Homan KJ. Self-Compassion and Psychological Well-Being in Older Adults. J Adult Dev. 2016;23: 111–119. doi: 10.1007/s10804-016-9227-8

[57] Kotera Y, Van Gordon W. Effects of Self-Compassion Training on Work-Related Well-Being: A Systematic Review. Frontiers in Psychology. 2021;12. Available: https://www.frontiersin.org/articles/10.3389/fpsyg.2021.630798. doi: 10.3389/fpsyg.2021.630798 33967896 PMC8102699

[58] Lee EE, Govind T, Ramsey M, Wu TC, Daly R, Liu J, et al. Compassion toward others and self-compassion predict mental and physical well-being: a 5-year longitudinal study of 1090 community-dwelling adults across the lifespan. Transl Psychiatry. 2021;11: 1–9. doi: 10.1038/s41398-021-01491-8 34282145 PMC8287292

[59] Tran US, Glück TM, Nader IW. Investigating the Five Facet Mindfulness Questionnaire (FFMQ): Construction of a Short Form and Evidence of a Two-Factor Higher Order Structure of Mindfulness. Journal of Clinical Psychology. 2013;69: 951–965. doi: 10.1002/jclp.21996 23784693

[60] Germer CK, Barnhofer T. Mindfulness and compassion: Similarities and differences. In: Gilbert P, editor. Compassion: Concepts, research and applications. New York, NY: Taylor and Francis; 2017. pp. 69–86.

[61] Khoury B, Knäuper B, Pagnini F, Trent N, Chiesa A, Carrière K. Embodied Mindfulness. Mindfulness. 2017;8: 1160–1171. doi: 10.1007/s12671-017-0700-7

[62] Mackintosh K, Power K, Schwannauer M, Chan SWY. The Relationships Between Self-Compassion, Attachment and Interpersonal Problems in Clinical Patients with Mixed Anxiety and Depression and Emotional Distress. Mindfulness. 2018;9: 961–971. doi: 10.1007/s12671-017-0835-6 29875883 PMC5968043

[63] Pepping CA, Davis PJ, O’Donovan A, Pal J. Individual Differences in Self-Compassion: The Role of Attachment and Experiences of Parenting in Childhood. Self and Identity. 2015;14: 104–117. doi: 10.1080/15298868.2014.955050

[64] Raque-Bogdan TL, Ericson SK, Jackson J, Martin HM, Bryan NA. Attachment and mental and physical health: Self-compassion and mattering as mediators. Journal of Counseling Psychology. 2011;58: 272–278. doi: 10.1037/a0023041 21463033

[65] Wei M, Liao KY-H, Ku T-Y, Shaffer PA. Attachment, Self-Compassion, Empathy, and Subjective Well-Being Among College Students and Community Adults. Journal of Personality. 2011;79: 191–221. doi: 10.1111/j.1467-6494.2010.00677.x 21223269

[66] Acquadro C, Conway K, Hareendran A, Aaronson N, European Regulatory Issues and Quality of Life Assessment (ERIQA) Group. Literature review of methods to translate health-related quality of life questionnaires for use in multinational clinical trials. Value Health. 2008;11: 509–521. doi: 10.1111/j.1524-4733.2007.00292.x 18179659

[67] Beaton DE, Bombardier C, Guillemin F, Ferraz MB. Guidelines for the process of cross-cultural adaptation of self-report measures. SPINE. 2000;25: 3186–3191. doi: 10.1097/00007632-200012150-00014 11124735

[68] Brislin RW. Back-Translation for Cross-Cultural Research. Journal of Cross-Cultural Psychology. 1970;1: 185–216. doi: 10.1177/135910457000100301

[69] Jackson DL. Revisiting Sample Size and Number of Parameter Estimates: Some Support for the N:q Hypothesis. Structural Equation Modeling: A Multidisciplinary Journal. 2003;10: 128–141. doi: 10.1207/S15328007SEM1001_6

[70] Kline RB. Principles and Practice of Structural Equation Modeling, Fourth Edition. Guilford Publications; 2015.

[71] Schumacker RE, Lomax RG. A Beginner’s Guide to Structural Equation Modeling. 4th ed. London, UK: Routledge; 2016.

[72] Kyriazos TA. Applied Psychometrics: Sample Size and Sample Power Considerations in Factor Analysis (EFA, CFA) and SEM in General. Psychology. 2018;9: 2207–2230. doi: 10.4236/psych.2018.98126

[73] Comrey AL. Factor-analytic methods of scale development in personality and clinical psychology. J Consult Clin Psychol. 1988;56: 754–761. doi: 10.1037//0022-006x.56.5.754 3057010

[74] Hoe SL. Issues and Procedures in Adopting Structural Equation Modeling Technique. Journal of Applied Quantitative Methods. 2008;3: 76–83.

[75] Singh K, Junnarkar M, Kaur J. Measures of Positive Psychology, Development and Validation. Berlin; 2016.

[76] Comrey AL, Lee HB. A first course in factor analysis. Psychology press; 1992.

[77] Martin D. French translation of the DASS. In: Depression Anxiety Stress Scales [Internet]. [cited 1 Aug 2022]. Available: http://www2.psy.unsw.edu.au/Groups/Dass/French/French.htm.

[78] Lovibond PF. Depression Anxiety Stress Scales ‐ DASS. In: Psychology Foundation of Australia and University of New South Wales [Internet]. 22 Jun 2022 [cited 1 Aug 2022]. Available: http://www2.psy.unsw.edu.au/groups/dass/.

[79] Fraley RC, Waller NG, Brennan KA. An item response theory analysis of self-report measures of adult attachment. Journal of Personality and Social Psychology. 2000;78: 350–365. doi: 10.1037//0022-3514.78.2.350 10707340

[80] Favez N, Tissot H, Ghisletta P, Golay P, Notari SC. Validation of the French Version of the Experiences in Close Relationships–Revised (ECR-R) Adult Romantic Attachment Questionnaire. Swiss Journal of Psychology. 2016 [cited 20 Mar 2020]. Available: https://econtent.hogrefe.com/doi/abs/10.1024/1421-0185/a000177.

[81] Heeren A, Douilliez C, Peschard V, Debrauwere L, Philippot P. Cross-cultural validity of the Five Facets Mindfulness Questionnaire: Adaptation and validation in a French-speaking sample. Revue Européenne de Psychologie Appliquée/European Review of Applied Psychology. 2011;61: 147–151. doi: 10.1016/j.erap.2011.02.001

[82] Herrera JA, Lepage M-C, Gosselin P. Psychometric Properties of a Short Form of the Five Facets Mindfulness Questionnaire Among a Sample of French-Canadian Adolescents. Association for Contextual Behavioural Science; 2019 Jun; Dublin, Ireland.

[83] Cleare S, Gumley A, Cleare CJ, O’Connor RC. An Investigation of the Factor Structure of the Self-Compassion Scale. Mindfulness. 2018;9: 618–628. doi: 10.1007/s12671-017-0803-1 29599853 PMC5866823

[84] López A, Sanderman R, Smink A, Zhang Y, Sonderen E van, Ranchor A, et al. A Reconsideration of the Self-Compassion Scale’s Total Score: Self-Compassion versus Self-Criticism. PLoS One; San Francisco. 2015;10: e0132940. https://doi.org.ezproxy.library.ubc.ca/10.1371/journal.pone.0132940. doi: 10.1371/journal.pone.0132940 26193654 PMC4508060

[85] Muris P, van den Broek M, Otgaar H, Oudenhoven I, Lennartz J. Good and Bad Sides of Self-Compassion: A Face Validity Check of the Self-Compassion Scale and an Investigation of its Relations to Coping and Emotional Symptoms in Non-Clinical Adolescents. J Child Fam Stud. 2018;27: 2411–2421. doi: 10.1007/s10826-018-1099-z 30100697 PMC6061019

[86] Kotsou I, Leys C. Self-Compassion Scale (SCS): Psychometric Properties of The French Translation and Its Relations with Psychological Well-Being, Affect and Depression. PLOS ONE. 2016;11: e0152880. doi: 10.1371/journal.pone.0152880 27078886 PMC4831759

[87] Trousselard M, Steiler D, Dutheil F, Claverie D, Canini F, Fenouillet F, et al. Validation of the Warwick-Edinburgh Mental Well-Being Scale (WEMWBS) in French psychiatric and general populations. Psychiatry Research. 2016;245: 282–290. doi: 10.1016/j.psychres.2016.08.050 27565700

[88] Emery M, Brophy K, MacDonald C, Côté C, Annett K. Adaptation and validation of the Compassionate Engagement and Action Scales, Compassion Scale, and Sussex-Oxford Compassion Scales in a French-Canadian sample analysis scripts. Zenodo; 2024. doi: 10.5281/zenodo.10695133 PMC11195958 38913657

[89] Emery M, Brophy K, MacDonald C, Côté C, Körner A. Adaptation and validation of the Compassionate Engagement and Action Scales, Compassion Scale, and Sussex-Oxford Compassion Scales in a French-Canadian sample dataset. Zenodo; 2024. doi: 10.5281/zenodo.10695095 PMC11195958 38913657

[90] Rosseel Y. lavaan: An R Package for Structural Equation Modeling. Journal of Statistical Software. 2012;48: 1–36. doi: 10.18637/jss.v048.i02

[91] Rosseel Y, Jorgensen, Rockwood N. lavaan: Latent Variable Analysis version 0.6–12 from CRAN. 2021. Available: https://lavaan.ugent.be.

[92] Jorgensen TD, Pornprasertmanit S, Schoemann AM, Rosseel Y. semTools: Useful tools for structural equation modeling. 2022. Available: https://CRAN.R-project.org/package=semTools.

[93] Revelle W. psych: Procedures for Psychological, Psychometric, and Personality Research. Evanston Illinois: Northwestern University; 2021. Available: https://mran.revolutionanalytics.com/snapshot/2021-09-26/web/packages/psych/psych.pdf.

[94] Bentler PM. Comparative fit indexes in structural models. Psychol Bull. 1990;107: 238–246. doi: 10.1037/0033-2909.107.2.238 2320703

[95] Bentler PM, Bonett DG. Significance tests and goodness of fit in the analysis of covariance structures. Psychological Bulletin. 1980;88: 588–606. doi: 10.1037/0033-2909.88.3.588

[96] Steiger JH. Structural Model Evaluation and Modification: An Interval Estimation Approach. Multivariate Behavioral Research. 1990;25: 173–180. doi: 10.1207/s15327906mbr2502_4 26794479

[97] Hu L, Bentler PM. Cutoff criteria for fit indexes in covariance structure analysis: Conventional criteria versus new alternatives. Structural Equation Modeling: A Multidisciplinary Journal. 1999;6: 1–55. doi: 10.1080/10705519909540118

[98] Akaike H. A new look at the statistical model identification. IEEE Transactions on Automatic Control. 1974;19: 716–723. doi: 10.1109/TAC.1974.1100705

[99] Williams MJ, Dalgleish T, Karl A, Kuyken W. Examining the factor structures of the five facet mindfulness questionnaire and the self-compassion scale. Psychol Assess. 2014;26: 407–418. doi: 10.1037/a0035566 24490681

[100] Burnham KP, Anderson DR 1942-. Information and Likelihood Theory: A Basis for Model Selection and Inference. 2nd ed. Model selection and multimodel inference: a practical information-theoretic approach. 2nd ed. New York: Springer; 2002. pp. 49–97.

[101] McNeish D. Thanks coefficient alpha, we’ll take it from here. Psychological Methods. 2018;23: 412–433. doi: 10.1037/met0000144 28557467

[102] Taber KS. The Use of Cronbach’s Alpha When Developing and Reporting Research Instruments in Science Education. Res Sci Educ. 2018;48: 1273–1296. doi: 10.1007/s11165-016-9602-2

[103] Terwee CB, Bot SDM, de Boer MR, van der Windt DAWM, Knol DL, Dekker J, et al. Quality criteria were proposed for measurement properties of health status questionnaires. J Clin Epidemiol. 2007;60: 34–42. doi: 10.1016/j.jclinepi.2006.03.012 17161752

[104] Vandenberg RJ, Lance CE. A Review and Synthesis of the Measurement Invariance Literature: Suggestions, Practices, and Recommendations for Organizational Research. Organizational Research Methods. 2000;3: 4–70. doi: 10.1177/109442810031002

[105] de Krijger E, Willems R, ten Klooster P, Bakker E, Miedema H, Drossaert C, et al. Further Validation of a Dutch Translation of the Sussex Oxford Compassion for the Self Scale in Samples of Crisis Line Volunteers, Military Personnel and Nursing Students. Frontiers in Psychology. 2022;13. Available: https://www.frontiersin.org/articles/10.3389/fpsyg.2022.895850. doi: 10.3389/fpsyg.2022.895850 35859833 PMC9289624

[106] Benjamini Y, Hochberg Y. Controlling the False Discovery Rate: A Practical and Powerful Approach to Multiple Testing. Journal of the Royal Statistical Society: Series B (Methodological). 1995;57: 289–300. doi: 10.1111/j.2517-6161.1995.tb02031.x

[107] Cohen J. Statistical power analysis for the behavioural sciences. 2nd ed. Hillsdale, N.J.: Lawrence Erlbaum Associates; 1988.

[108] Halamová J, Kanovský M, Pacúchová M. Psychometric Analysis of the Slovak Version of the Compassionate Engagement and Action Scales. Journal of Psychological and Educational Research. 2020;28: 64–80.

[109] Murfield J, Moyle W, O’Donovan A, Ware RS. Validity of the Compassionate Engagement and Action Scales with family carers of older adults: confirmatory factor analyses. International Psychogeriatrics. 2021;33: 373–383. doi: 10.1017/S1041610220001738 32928331

[110] Asano K, Kotera Y, Tsuchiya M, Ishimura I, Lin S, Matsumoto Y, et al. The development of the Japanese version of the compassionate engagement and action scales. PLOS ONE. 2020;15: e0230875. doi: 10.1371/journal.pone.0230875 32236112 PMC7112184

[111] Sonderen E van, Sanderman R, Coyne JC. Ineffectiveness of Reverse Wording of Questionnaire Items: Let’s Learn from Cows in the Rain. PLOS ONE. 2013;8: e68967. doi: 10.1371/journal.pone.0068967 PMC3729568 23935915

[112] Lucarini A, Fuochi G, Voci A. A deep dive into compassion: Italian validation, network analysis, and correlates of recent compassion scales. European Journal of Psychological Assessment. 2022; 371–384. doi: 10.1027/1015-5759/a000717

[113] Halamová J, Kanovský M. Factor Structure of the Sussex-Oxford Compassion Scales. Psychological Topics. 2021;30: 489–508.

[114] Kim J, Seo J-W. Assessing Compassion in Korean Population: Psychometric Properties of the Korean Version of Sussex-Oxford Compassion Scales. Frontiers in Psychology. 2021;12: 744481. doi: 10.3389/fpsyg.2021.744481 34707546 PMC8544640

[115] Sarling A, Sundin Ö, Åhs F, Gu J, Jansson B. Factor structure and psychometric properties of a Swedish version of the Sussex-Oxford Compassion Scales (SOCS). Nordic Psychology. 2022; 1–19. doi: 10.1080/19012276.2022.2156381

[116] Dudley J, Eames C, Mulligan J, Fisher N. Mindfulness of voices, self-compassion, and secure attachment in relation to the experience of hearing voices. British Journal of Clinical Psychology. 57: 1–17. doi: 10.1111/bjc.12153 28801978 PMC5811822

[117] Joeng JR, Turner SL, Kim EY, Choi SA, Kim JK, Lee YJ. Data for Korean college students׳ anxious and avoidant attachment, self-compassion, anxiety and depression. Data in Brief. 2017;13: 316–319. doi: 10.1016/j.dib.2017.06.006 28653024 PMC5476454

[118] Navarro-Gil M, Lopez-del-Hoyo Y, Modrego-Alarcón M, Montero-Marin J, Gordon WV, Shonin E, et al. Effects of Attachment-Based Compassion Therapy (ABCT) on Self-compassion and Attachment Style in Healthy People. Mindfulness. 2018; 1–12. doi: 10.1007/s12671-018-0896-1

[119] Raque-Bogdan TL, Piontkowski S, Hui K, Ziemer KS, Garriott PO. Self-compassion as a mediator between attachment anxiety and body appreciation: An exploratory model. Body Image. 2016;19: 28–36. doi: 10.1016/j.bodyim.2016.08.001 27597725

[120] Gilbert P, McEwan K, Catarino F, Baião R, Palmeira L. Fears of happiness and compassion in relationship with depression, alexithymia, and attachment security in a depressed sample. British Journal of Clinical Psychology. 2014;53: 228–244. doi: 10.1111/bjc.12037 24283291

[121] Joeng JR, Turner SL, Kim EY, Choi SA, Lee YJ, Kim JK. Insecure attachment and emotional distress: Fear of self-compassion and self-compassion as mediators. Personality and Individual Differences. 2017;112: 6–11. doi: 10.1016/j.paid.2017.02.048

[122] Cameron JJ, Finnegan H, Morry MM. Orthogonal dreams in an oblique world: A meta-analysis of the association between attachment anxiety and avoidance. Journal of Research in Personality. 2012;46: 472–476. doi: 10.1016/j.jrp.2012.05.001

[123] Gilbert P. Compassion: From Its Evolution to a Psychotherapy. Frontiers in Psychology. 2020;11. Available: https://www.frontiersin.org/articles/10.3389/fpsyg.2020.586161. 33362650 10.3389/fpsyg.2020.586161 PMC7762265

[124] Arch JJ, Brown KW, Dean DJ, Landy LN, Brown K, Laudenslager ML. Self-compassion training modulates alpha-amylase, heart rate variability, and subjective responses to social evaluative threat in women. Psychoneuroendocrinology. 2014;42: 49–58. doi: 10.1016/j.psyneuen.2013.12.018 24636501 PMC3985278

[125] Svendsen JL, Osnes B, Binder P-E, Dundas I, Visted E, Nordby H, et al. Trait Self-Compassion Reflects Emotional Flexibility Through an Association with High Vagally Mediated Heart Rate Variability. Mindfulness (N Y). 2016;7: 1103–1113. doi: 10.1007/s12671-016-0549-1 27642372 PMC5010618

[126] Kirby JN, Doty JR, Petrocchi N, Gilbert P. The Current and Future Role of Heart Rate Variability for Assessing and Training Compassion. Front Public Health. 2017;5. doi: 10.3389/fpubh.2017.00040 28337432 PMC5340770

[127] Luo X, Qiao L, Che X. Self-compassion Modulates Heart Rate Variability and Negative Affect to Experimentally Induced Stress. Mindfulness. 2018;9: 1522–1528. doi: 10.1007/s12671-018-0900-9

[128] Di Bello M, Carnevali L, Petrocchi N, Thayer JF, Gilbert P, Ottaviani C. The compassionate vagus: A meta-analysis on the connection between compassion and heart rate variability. Neuroscience & Biobehavioral Reviews. 2020;116: 21–30. doi: 10.1016/j.neubiorev.2020.06.016 32554001

[129] Di Bello M, Ottaviani C, Petrocchi N. Compassion Is Not a Benzo: Distinctive Associations of Heart Rate Variability With Its Empathic and Action Components. Frontiers in Neuroscience. 2021;15. Available: https://www.frontiersin.org/articles/10.3389/fnins.2021.617443. 10.3389/fnins.2021.617443 PMC7994334 33776635

[130] Daouk-Öyry L, McDowal A. Using Cognitive Interviewing for the Semantic Enhancement of Multilingual Versions of Personality Questionnaires. Journal of Personality Assessment. 2013;95: 407–416. doi: 10.1080/00223891.2012.735300 23113474

[131] Byrne BM. Adaptation of assessment scales in cross-national research: Issues, Guidelines, and Caveats. International Perspectives in Psychology: Research, Practice, Consultation. 2016;5: 51–65. doi: 10.1037/ipp0000042

[132] Statistics Canada. Census Profile Table, 2021 Census of Population ‐ Quebec [Province]. 2023. Available: https://www12.statcan.gc.ca/census-recensement/2021/dp-pd/prof/index.cfm?Lang=E.

