# Supplementary material for: Validation of the compassionate engagement and action scales, compassion scale, and Sussex-Oxford compassion scales in a French-Canadian sample
Source: PLoS One. 2024 Jun 24;19(6):e0305776. doi: 10.1371/journal.pone.0305776 (PMC11195958; doi:10.1371/journal.pone.0305776)
Supplement: S1 Appendix — Model Estimators; Scale Development Summary (S1 Table in S2 Appendix); Floor and Ceiling Effects (S2 Table in S2 Appendix); Pearson Correlation Coefficients (S3 Table in S2 Appendix); Path Diagrams (S1-S18 Figs in S2 Appendix). (DOCX) [file pone.0305776.s001.docx]

**The Compassionate Engagement and Action Scales**

***Les échelles de l’engagement et de l’action dans la compassion***

English Version: Gilbert, P., Catarino, F., Duarte, C., Matos, M., Kolts, R., Stubbs, J., Ceresatto, L., Duarte, J., Pinto-Gouveia, J., & Basran, J. (2017). The development of compassionate engagement and action scales for self and others. *Journal of Compassionate Health Care*, *4*. <https://doi.org/10.1186/s40639-017-0033-3>

French translation: Brophy, K., Emery, M., MacDonald, C., Côté, C., & Körner, A. (2024). Validation of the Compassionate Engagement and Action Scales, Compassion Scale, and Sussex-Oxford Compassion Scales in a French-Canadian sample. *PLoS ONE.* <https://doi.org/10.1371/journal.pone.0305776>

| ***L’auto-compassion*** |
| --- |
| Lorsque les choses vont mal pour nous et que nous ressentons de la détresse vis-à-vis des difficultés, des échecs, des déceptions ou des pertes que nous vivons, nous pouvons faire face à cette détresse de différentes manières. Nous nous intéressons au degré de compassion dont les gens peuvent **faire preuve envers eux-mêmes**. Nous définissons la compassion comme « une sensibilité à la souffrance en soi et chez les autres accompagnée d’un dévouement à essayer de l’atténuer et de la prévenir. » Cela signifie que la compassion présente deux composantes. La *première* est la motivation à se mobiliser envers les choses et les émotions qui sont difficiles, au lieu de les éviter ou de les réprimer. La *deuxième* composante de la compassion est la capacité à se concentrer sur ce qui nous est utile. Tout comme un médecin avec son patient. La première consiste à être capable et motivé(e) à porter attention à la douleur et à la comprendre (ou à apprendre à la comprendre). La deuxième est d’être capable de prendre des mesures qui seront utiles. Vous trouverez ci-dessous une série de questions portant sur ces deux composantes de la compassion. Veuillez donc lire attentivement chaque énoncé en pensant à la manière dont cela s’applique à vous lorsque vous vivez de la détresse. Veuillez évaluer les items au moyen de l’échelle suivante :  Jamais-Toujours (1-10) |
| **Section 1 – Voici des questions concernant votre niveau de motivation et votre capacité à faire face à la détresse que vous ressentez. Donc :**  **Lorsque je suis en détresse, bouleversé(e), ou contrarié(e) par quelque chose …** |
| 1. Je suis *motivé(e)* à m’engager et à gérer ma détresse lorsqu’elle survient. |
| 1. Je *remarque* et je suis *sensible* à mes sentiments de détresse lorsqu’ils surviennent. |
| 1. J’évite de penser à ma détresse et j’essaie de me distraire et de la chasser de mon esprit.   (r) |
| 1. Je suis *ému(e)* ou émotionnellement touché(e) par les situations ou sentiments de détresse que je vis. |
| 1. Je *tolère* les divers sentiments qui font partie de ma détresse. |
| 1. Je *réfléchis* sur les sentiments de détresse que je ressens et j’essaie de les *comprendre*. |
| 1. Je ne tolère pas de ressentir de la détresse.   (r) |
| 1. J’*accepte*, je suis *non-critique* et je *ne porte pas de jugement* sur mes sentiments de détresse. |
| **Section 2 – Ces questions concernent les façons dont vous faites activement face, avec compassion, aux émotions, aux pensées et aux situations qui provoquent de la détresse en vous. Donc :**  **Lorsque je suis en détresse, bouleversé(e), ou contrarié(e) par quelque chose …** |
| 1. Je dirige mon *attention* vers ce qui pourrait m’aider. |
| 1. Je *réfléchis* et je trouve des façons utiles pour faire face à ma détresse. |
| 1. Je ne sais pas comment m’aider moi-même.   (r) |
| 1. Je prends les *mesures* et fais les choses qui pourront m’aider. |
| 1. Je génère en moi des sentiments de *soutien*, d’*aide* et d’*encouragement*. |
| **À NOTER : LES ITEMS INVERSÉS (r) NE SONT PAS INCLUS DANS LA NOTATION** |

| ***Compassion envers les autres*** |
| --- |
| Lorsque les choses vont mal pour les autres et qu’ils ou elles ressentent de la détresse vis-à-vis des difficultés, des échecs, des déceptions ou des pertes qu’ils ou elles vivent, nous pouvons faire face à leur détresse de différentes manières. Nous nous intéressons au degré de **compassion** dont les gens peuvent faire preuve **envers les autres**. Nous définissons la compassion comme « une sensibilité à la souffrance en soi et chez les autres accompagnée d’un dévouement à essayer de l’atténuer et de la prévenir. » Cela signifie que la compassion présente deux composantes. La *première* est la motivation à se mobiliser envers les choses et les émotions qui sont difficiles, au lieu de les éviter ou de les réprimer. La *deuxième* composante de la compassion est la capacité à se concentrer sur ce qui nous est utile. Tout comme un médecin avec son patient. La première est d’être motivé et capable de prêter attention à la douleur et (apprendre à) la comprendre. La deuxième est d’être capable de prendre des mesures qui seront utiles. Vous trouverez ci-dessous une série de questions portant sur ces deux composantes de la compassion. Veuillez donc lire attentivement chaque énoncé en pensant à la manière dont cela s’applique à vous lorsque des **personnes dans votre vie vivent de la détresse**. Veuillez évaluer les items au moyen de l’échelle suivante :  Jamais-Toujours (1-10) |
| **Section 1 – Voici des questions concernent votre niveau de motivation et votre capacité à faire face à la détresse que d’autres personnes ressentent. Donc :**  **Lorsque je suis en détresse, bouleversé(e), ou contrarié(e) par quelque chose …** |
| Je suis *motivé(e)* à m’engager et à gérer la détresse des autres lorsqu’elle survient. |
| Je *remarque* et je suis *sensible* à la détresse des autres lorsqu’elle survient. |
| J’évite de penser à la détresse des autres et j’essaie de me distraire et de la chasser de mon esprit.  (r) |
| Je suis *ému(e)* ou émotionnellement touché(e) par les manifestations de détresse des autres. |
| Je *tolère* les divers sentiments qui font partie de la détresse des autres. |
| Je *réfléchis* sur et j’*essaie de comprendre* la détresse des autres. |
| Je ne tolère pas la détresse des autres.  (r) |
| J’*accepte*, je suis *non-critique* et je ne *porte pas de jugement* sur la détresse des autres. |
| **Section 2 – Ces questions concernent les façons dont vous répondez activement, avec compassion, lorsque d’autres personnes vivent de la détresse. Donc :**  **Lorsque je suis en détresse, bouleversé(e), ou contrarié(e) par quelque chose …** |
| Je dirige mon *attention* vers ce qui pourrait aider les autres. |
| Je *réfléchis et trouve* des façons utiles pour que les autres puissent faire face à leur détresse. |
| Je ne sais pas comment aider les autres lorsqu’ils ou elles ressentent de la détresse.  (r) |
| Je prends les *mesures* et *fais les choses* qui pourront aider les autres. |
| J’exprime des sentiments de *soutien,* d’*aide* et d’*encouragement* envers les autres. |
| **À NOTER : LES ITEMS INVERSÉS (r) NE SONT PAS INCLUS DANS LA NOTATION** |

| ***Compassion de la part des autres*** |
| --- |
| Lorsque les choses vont mal pour nous et que nous ressentons de la détresse vis-à-vis des difficultés, des échecs, des déceptions ou des pertes que nous vivons, les autres peuvent faire face à notre détresse de différentes manières. Nous nous intéressons au degré de compassion dont les personnes importantes dans votre vie font preuve lorsque **vous êtes en détresse**. Nous définissons la compassion comme « une sensibilité à la souffrance en soi et chez les autres accompagnée d’un dévouement à essayer de l’atténuer et de la prévenir. » Cela signifie que la compassion présente deux composantes. La *première* est la motivation à se mobiliser envers les choses et les émotions qui sont difficiles, au lieu de les éviter ou de les réprimer. La *deuxième* composante de la compassion est la capacité à se concentrer sur ce qui nous est utile. Tout comme un médecin avec son patient. La première est d’être motivé et capable de prêter attention à la douleur et (apprendre à) la comprendre. La deuxième est d’être capable de prendre des mesures qui seront utiles. Vous trouverez ci-dessous une série de questions portant sur ces deux composantes de la compassion. Veuillez donc lire attentivement chaque énoncé en pensant à la manière dont cela s’applique aux personnes importantes de votre vie lorsque vous vivez de la détresse. Veuillez évaluer les items au moyen de l’échelle suivante :  Jamais-Toujours (1-10) |
| **Section 1 – Voici des questions mesurant à quel point vous pensez que les autres sont motivés et à quel point qu’ils ou elles s’impliquent dans votre détresse lorsque vous en faites l’expérience. Donc :**  **Lorsque je suis en détresse, bouleversé(e), ou contrarié(e) par quelque chose …** |
| 1. Les autres personnes sont activement *motivées* à s’engager et à gérer ma détresse lorsqu’elle survient. |
| 1. Les autres *remarquent* et sont *sensible*s à mes sentiments de détresse lorsqu’ils surviennent. |
| 1. Les autres évitent de penser à ma détresse, essaient de se distraire et de la chasser de leur esprit.   (r) |
| 1. Les autres sont *ému(e)s* ou *émotionnellement touché(e)s* par mes sentiments de détresse. |
| 1. Les autres *tolèrent* les divers sentiments qui font partie de ma détresse. |
| 1. Les autres *réfléchissent* sur mes sentiments de détresse et *essaient de les comprendre.* |
| 1. Les autres ne tolèrent pas ma détresse.   (r) |
| 1. Les autres *acceptent*, *ne critiquent pas* et *ne jugent pas* mes sentiments de détresse. |
| **Section 2 – Ces questions concernent les façons dont les autres font activement face, avec compassion, aux émotions et aux situations qui provoquent de la détresse en vous. Donc :**  **Lorsque je suis en détresse, bouleversé(e), ou contrarié(e) par quelque chose …** |
| 1. Les autres dirigent leur *attention* vers ce qui est susceptible de m’aider. |
| 1. Les autres *pensent à* et trouvent des façons utiles afin que je puisse faire face à ma détresse. |
| 1. Les autres ne savent pas comment m’aider lorsque je ressens de la détresse.   (r) |
| 1. Les autres prennent des *mesures* et *font des choses* qui m’aideront. |
| 1. Les autres me traitent avec des sentiments de *soutien*, d’*aide* et d’*encouragement*. |
| **À NOTER : LES ITEMS INVERSÉS (r) NE SONT PAS INCLUS DANS LA NOTATION** |
| **NOTATION**  Les trois échelles – Compassion pour les autres, compassion de la part des autres, et compassion pour soi sont notées séparément.  Pour chaque échelle, deux sous-échelles peuvent être calculées : l’échelle portant sur l’engagement (items 1, 2, 4, 5, 6, 8) et l’échelle portant sur les actions (1, 2, 4, 5).  Pour l’échelle *Compassion pour soi*, deux dimensions peuvent être analysées dans la sous-échelle Engagement (la somme des items 2 et 4, et la somme des items 1, 5, 6, et 8).  Un score total peut être calculé (la somme des items des sous-échelles Engagement et Actions) pour chaque échelle – *Compassion pour les autres, compassion de la part des autres,* et *compassion pour soi*.  Veuillez noter que les items inversés (r) ne sont pas inclus dans la notation.  **DESCRIPTION**  *Les échelles de l’engagement et de l’action dans la compassion*  Les échelles de l’engagement et de l’action dans la compassion sont trois échelles qui mesurent l’auto-compassion (« Je suis *motivé(e)* à m’engager et à gérer ma détresse lorsqu’elle survient »), la capacité à ressentir de la compassion envers les autres en détresse (« Je suis *motivé(e)* à m’engager et à gérer la détresse des autres lorsqu’elle survient ») et la capacité à recevoir de la compassion de la part de personnes importantes dans la vie du répondant (« Les personnes autour de moi sont motivées à s’engager et à confronter ma détresse lorsqu’elle survient »). Dans la première section de chaque échelle, six items sont formulés pour refléter les six attributs retrouvés dans le modèle CFT : la sensibilité à la souffrance, la sympathie, le non-jugement, l’empathie, la tolérance à la détresse, et le soin du bien-être. Ces sections comprennent également deux éléments de remplissage inversés. La deuxième section de l’échelle comprend quatre autres items qui reflètent des actions de compassion spécifiques pour faire face à la détresse et un item de remplissage inversé. Les participants sont invités à évaluer chaque énoncé en fonction de la fréquence à laquelle cela se produit sur une échelle de 1 à 10 (1 = Jamais ; 10 = Toujours). |

**Compassion Scale**

***L’échelle de compassion (EC)***

English version: Pommier, E. A., Neff, K., & Tóth-Király, I. (2020). The Development and Validation of the Compassion Scale. *Assessment*, *27*(1), 21–39. <https://doi.org/10.1177/1073191119874108>

French translation: Brophy, K., Emery, M., MacDonald, C., Côté, C., & Körner, A. (2024). Validation of the Compassionate Engagement and Action Scales, Compassion Scale, and Sussex-Oxford Compassion Scales in a French-Canadian sample. *PLoS ONE.* <https://doi.org/10.1371/journal.pone.0305776>

|  |
| --- |
| Veuillez lire attentivement chaque énoncé avant de répondre. Indiquez la fréquence à laquelle vous vous sentez ou vous comportez de la manière indiquée sur une échelle de 1 « presque jamais » à 5 « presque toujours ». Veuillez répondre selon votre expérience réelle et non selon ce que vous croyez que votre expérience devrait être. |
| 1. J’accorde une attention toute particulière aux autres personnes lorsqu’elles me parlent de leurs problèmes. |
| 1. Si je vois que quelqu’un vit une période difficile, j’essaie d’être attentionné(e) envers cette personne. |
| 1. Je reste indiffèrent vis à vis les problèmes des autres. |
| 1. Je suis conscient(e) que tout le monde peut parfois se sentir déprimé(e), cela fait partie de la nature humaine. |
| 1. Je remarque quand les gens sont contrarié(e)s, même s’ils/elles ne disent rien. |
| 1. J’aime être là pour les autres lorsqu’ils/elles traversent des périodes difficiles. |
| 1. Je passe peu de temps à penser aux soucis des autres. |
| 1. Je crois qu’il est important de reconnaître que tout le monde a des faiblesses et que personne n’est parfait. |
| 1. J’écoute patiemment lorsque les gens me parlent de leurs problèmes. |
| 1. Je me sens de tout cœur avec les personnes qui sont malheureuses. |
| 1. J’essaie d’éviter les personnes qui éprouvent beaucoup de douleur. |
| 1. Je pense que la souffrance fait simplement partie de l’expérience humaine commune. |
| 1. Lorsque les gens me parlent de leurs problèmes, j’essaie de garder un point de vue équilibré de la situation. |
| 1. Lorsque les autres ressentent de la tristesse, j’essaie de les réconforter. |
| 1. Je n’arrive pas vraiment à établir un lien avec les autres lorsqu’ils/elles souffrent. |
| 1. Malgré mes différences avec les autres, je sais que, comme moi, tout le monde ressent de la douleur. |
| Système de codage  Items liés à la bienveillance : 2, 6, 10, 14  Items liés à l’expérience humaine commune : 4, 8, 12, 16  Items liés à la pleine conscience : 1, 5, 9, 13  Items liés à l’indifférence (cotation inverse) : 3, 7, 11, 15  Pour calculer un score total de compassion, prenez la moyenne de tous les items. |

**Sussex-Oxford Compassion Scales for Self and Others Scale**

English Version: Gu, J., Baer, R., Cavanagh, K., Kuyken, W., & Strauss, C. (2020). Development and Psychometric Properties of the Sussex-Oxford Compassion Scales (SOCS). *Assessment*, *27*(1), 3–20. <https://doi.org/10.1177/1073191119860911>

French translation: Brophy, K., Emery, M., MacDonald, C., Côté, C., & Körner, A. (2024). Validation of the Compassionate Engagement and Action Scales, Compassion Scale, and Sussex-Oxford Compassion Scales in a French-Canadian sample. *PLoS ONE.* <https://doi.org/10.1371/journal.pone.0305776>

| *Questionnaire de compassion pour les autres* |
| --- |
| Vous trouverez ci-dessous des énoncés décrivant comment vous pouvez vous identifier à **d’autres personnes** et les comprendre. Merci d’indiquer à quel point les affirmations suivantes sont vraies pour vous en utilisant l’échelle en 5 points (1=pas du tout vrai ; 2=rarement vrai ; 3=parfois vrai ; 4=souvent vrai ; 5=toujours vrai). Par exemple, si vous pensez qu’une affirmation est souvent vraie pour vous, vous entourez le chiffre « 4 ».  Note : dans les énoncés ci-dessous, des termes génériques (p.ex : « contrarié(e) », « bouleversé(e) », « en souffrance », « en difficulté », etc.) sont utilisés pour couvrir un large éventail d’émotions désagréables comme la tristesse, la peur, la colère, la frustration, la culpabilité, la honte, etc.  Veuillez fournir une réponse pour chaque énoncé. |
| 1. Je reconnais lorsque les autres se sentent en détresse sans qu’ils aient à me le dire |
| 1. Je comprends que tout le monde souffre à certains moments de sa vie |
| 1. Lorsque quelqu’un vit une passe difficile, j’agis de façon bienveillante avec elle/lui |
| 1. Lorsque quelqu’un est bouleversé/contrarié, j’essaie de rester ouvert(e) à ses sentiments plutôt que de les éviter |
| 1. Lorsque d’autres personnes rencontrent des difficultés, j’essaie de faire des choses qui pourraient être utiles |
| 1. Je remarque quand d’autres personnes se sentent en détresse |
| 1. Je sais que se sentir bouleversé(e)/ contrarié(e) par moments fait partie de la nature humaine |
| 1. Lorsque j’apprends que de mauvaises choses arrivent à d’autres, je suis inquiet(ète) pour leur bien être |
| 1. Lorsque d’autres personnes sont contrarié(e)s/bouleversé(e)s, je reste avec elles et je les écoute, même si c’est difficile |
| 1. Quand quelqu’un traverse une période difficile, j’essaie de prendre soin d’elle/lui |
| 1. Je repère rapidement les premiers signes de détresse chez les autres |
| 1. Je sais que, comme moi, d’autres personnes rencontrent aussi des difficultés dans leur vie |
| 1. Lorsque quelqu’un est bouleversé~~(e)~~/ contrarié~~(e)~~, j’essaie d’être à l’écoute de ce qu’ils ressentent |
| 1. Je me connecte avec la souffrance des autres sans les juger |
| 1. Quand je vois quelqu’un qui a besoin d’aide, j’essaie de faire ce qu’il y a de mieux pour elle/lui |
| 1. J’identifie les signes de souffrance chez les autres |
| 1. Je sais que nous pouvons tous et toutes nous sentir bouleversé(e)s/contrarié(e)s lorsqu’on nous fait du tort. |
| 1. Je suis sensible à la détresse des autres |
| 1. Quand quelqu’un est bouleversé(e)/contrarié(e), je peux être présent(e) pour elle/lui sans être submergé(e) par sa détresse |
| 1. Quand je vois quelqu’un qui est bouleversé/contrarié, je fais de mon mieux pour prendre soin d’elle/lui |
| **Guide de notation**  Les utilisateurs peuvent calculer à la fois les scores totaux des sous-échelles et le score total SOCS-O. Pour calculer les scores totaux des sous-échelles, additionnez les scores de chaque item, et ce pour chaque sous-échelle (voir ci-dessous les items associés à chaque sous-échelle). Pour calculer le score total SOCS-O, additionnez le score total des cinq sous-échelles ou additionnez le score des 20 items.  Items liés à la reconnaissance de la souffrance : 1, 6, 11, 16.  Items liés à la compréhension de l’universalité de la souffrance : 2, 7, 12, 17.  Items liés à la sensibilité envers la personne qui souffre : 3, 8, 13, 18.  Items liés à la tolérance face aux sentiments inconfortables : 4, 9, 14, 19.  Items liés à l’action ou la motivation à agir pour soulager la souffrance : 5, 10, 15, 20. |

| *Questionnaire de compassion pour soi* |
| --- |
| Vous trouverez ci-dessous des énoncés décrivant comment vous pouvez vous comprendre **vous-même**. Merci d’indiquer à quel point les affirmations suivantes sont vraies pour vous en utilisant l’échelle en 5 points (1=pas du tout vrai ; 2=rarement vrai ; 3=parfois vrai ; 4=souvent vrai ; 5=toujours vrai). Par exemple, si vous pensez qu’une affirmation est souvent vraie pour vous, vous entourez le chiffre « 4 ».  Note : dans les énoncés ci-dessous, des termes génériques (p.ex : « contrarié(e) », « bouleversé(e) », « en souffrance », « en difficulté », etc.) sont utilisés pour couvrir un large éventail d’émotions désagréables comme la tristesse, la peur, la colère, la frustration, la culpabilité, la honte, etc.  Veuillez fournir une réponse pour chaque énoncé. |
| 1. J’arrive bien à reconnaître quand je me sens en détresse |
| 1. Je comprends que tout le monde souffre à certains moments de sa vie. |
| 1. Lorsque je vis une passe difficile, j’agis de façon bienveillante avec moi-même |
| 1. Lorsque je suis bouleversé(e)/contrarié(e), j’essaie de rester ouvert(e) à mes sentiments plutôt que de les éviter |
| 1. J’essaie de faire en sorte de me sentir mieux lorsque je suis en détresse même si je ne peux pas agir sur la cause |
| 1. Je le remarque quand je me sens en détresse |
| 1. Je sais que se sentir bouleversé(e)/contrarié(e) par moments fait partie de la nature humaine |
| 1. Lorsque des choses difficiles m’arrivent, je me sens bienveillant(e) envers moi-même. |
| 1. J’entre en contact avec ma propre détresse sans me laisser envahir par elle |
| 1. Quand je traverse une période difficile, j’essaie de prendre soin de moi |
| 1. Je repère rapidement en moi les premiers signes de détresse |
| 1. Je sais que, comme moi, d’autres personnes font aussi l’expérience de difficultés dans leur vie |
| 1. Lorsque je suis bouleversé(e)/contrarié(e), j’entre en contact avec ce que je ressens. |
| 1. J’entre en contact avec ma propre souffrance sans me juger |
| 1. Quand je suis bouleversé(e)/contrarié(e), j’essaie de faire ce qu’il y a de mieux pour moi |
| 1. J’identifie les signes de souffrance en moi |
| 1. Je sais que nous pouvons tous nous sentir en détresse lorsque les choses ne vont pas bien dans notre vie |
| 1. Même quand je suis déçu(e) de moi, je peux me sentir bienveillant(e) envers moi-même lorsque je ressens de la détresse. |
| 1. Lorsque je suis bouleversé(e)/contrarié(e), je laisse mes émotions exister sans me sentir submergé(e) |
| 1. Quand je suis bouleversé(e)/contrarié(e), je fais de mon mieux pour prendre soin de moi |
| **Guide de notation**  Les utilisateurs peuvent calculer à la fois les scores totaux des sous-échelles et le score total SOCS-S. Pour calculer les scores totaux des sous-échelles, additionnez les scores de chaque item, et ce pour chaque sous-échelle (voir ci-dessous les items associés à chaque sous-échelle). Pour calculer le score total SOCS-S, additionnez le score total des cinq sous-échelles ou additionnez le score des 20 items.  Items liés à la reconnaissance de la souffrance : 1, 6, 11, 16.  Items liés à la compréhension de l’universalité de la souffrance : 2, 7, 12, 17.  Items liés à la sensibilité envers la personne qui souffre : 3, 8, 13, 18.  Items liés à la tolérance face aux sentiments inconfortables : 4, 9, 14, 19.  Items liés à l’action ou la motivation à agir pour soulager la souffrance : 5, 10, 15, 20. |
