# Supplementary material for: Validation of the compassionate engagement and action scales, compassion scale, and Sussex-Oxford compassion scales in a French-Canadian sample
Source: PLoS One. 2024 Jun 24;19(6):e0305776. doi: 10.1371/journal.pone.0305776 (PMC11195958; doi:10.1371/journal.pone.0305776)

**Validation of the Compassionate Engagement and Action Scales, Compassion Scale, and Sussex-Oxford Compassion Scales in a French-Canadian sample**

Kyla Brophy^1*^, Matthew Emery^2^, Ceilagh MacDonald^1^, Catherine Isadora Côté^3^, Annett Körner^1,4,5,6,7^

^1^Department of Counselling Psychology, McGill University, Montréal, Quebec, Canada

^2^Department of Statistics, University of British Columbia, Vancouver, British Columbia, Canada

^3^Department of Psychology, University of Montreal, Montréal, Quebec, Canada

^4^Lady Davis Institute, Jewish General Hospital, Montréal, Quebec, Canada

^5^Department of Oncology, McGill University, Montréal, Quebec, Canada

^6^Louise Granofsky Psychosocial Oncology Program, Segal Cancer Center, Montréal, Quebec, Canada

^7^Psychosocial Oncology Program, McGill University Health Centre, Montréal, Quebec, Canada

*** Corresponding author:**
Kyla Brophy: kyla.brophy@mail.mcgill.ca

S1 Appendix: Supporting Information

Table of Contents

[1 Model Estimators 3](#_Toc168584981)

[2 S1 Table. Scale Development Summary 4](#_Toc168584982)

[3 S2 Table. Floor and Ceiling Effects 7](#_Toc168584983)

[4 S3 Table. Pearson Correlation Coefficients adjusted by Benjamini-Hochberg Procedure. 10](#_Toc168584984)

[5 S1 Fig. CEAS-SC, One-Factor Model. 12](#_Toc168584985)

[6 S2 Fig. CEAS-SC, Two-Factor Model. 13](#_Toc168584986)

[7 S3 Fig. CEAS-SC, Two-Factor Hierarchical Model. 14](#_Toc168584987)

[8 S4 Fig. CEAS-SC, Three-Factor Model. 15](#_Toc168584988)

[9 S5 Fig. CEAS-TO, One-Factor Model. 16](#_Toc168584989)

[10 S6 Fig. CEAS-TO, Two-Factor Model. 17](#_Toc168584990)

[11 S7 Fig. CEAS-FROM, One-Factor Model. 18](#_Toc168584991)

[12 S8 Fig. CEAS-FROM, Two-Factor Model. 19](#_Toc168584992)

[13 S9 Fig. CEAS-FROM, Two-Factor Hierarchical Model. 20](#_Toc168584993)

[14 S10 Fig. Compassion Scale, One-Factor Model. 21](#_Toc168584994)

[15 S11 Fig. Compassion Scale, Four-Factor Model. 22](#_Toc168584995)

[16 S12 Fig. Four-Factor Hierarchical Model. 23](#_Toc168584996)

[17 S13 Fig. SOCS-S, One-Factor Model. 24](#_Toc168584997)

[18 S14 Fig. SOCS-S Five-Factor Model. 25](#_Toc168584998)

[19 S15 Fig. SOCS-S, Five-Factor Hierarchical Model. 26](#_Toc168584999)

[20 S16 Fig. SOCS-O, One-Factor Model. 27](#_Toc168585000)

[21 S17 Fig. SOCS-O, Four-Factor Model. 28](#_Toc168585001)

[22 S18 Fig. SOCS-O, Five-Factor Hierarchical Model. 29](#_Toc168585002)

# Model Estimators

As noted by Pommier et al. [1], the weighted least squares mean- and variance-adjusted estimator (WLSMV) was chosen as an estimator in developing the CS as it is best suited for ordered-categorical data with five or fewer response options [2]. This rationale was considered when choosing an estimator to analyze the translated scales, and while WLSMV appears best suited to analyze categorical data, there are other factors to consider [3]. While maximum likelihood estimation (MLR) assumes that items are continuous and normally distributed, the robust correction was introduced to improve model reliability when these assumptions are violated and is observed to perform well when used with ordered-categorical data [2–4]. Furthermore, it has been suggested that MLR outperforms WLSMV with small sample sizes [4]. Although WLSMV is well-suited to ordinal data, it requires a large sample size—in particular, each response category for each item in a scale should be observed multiple times. In the present study, certain responses for items in the CEAS and SOCS were rarely observed, and as such MLR was chosen as the appropriate estimator for these scales. As noted, the choice of estimator for each scale is consistent with the English studies.

**References**

1. Pommier EA, Neff K, Tóth-Király I. The Development and Validation of the Compassion Scale. Assessment. 2020 Jan 1;27(1):21–39.

2. Bandalos DL. Relative Performance of Categorical Diagonally Weighted Least Squares and Robust Maximum Likelihood Estimation. Structural Equation Modeling: A Multidisciplinary Journal. 2014 Jan 2;21(1):102–16.

3. Li CH. The performance of MLR, USLMV, and WLSMV estimation in structural regression models with ordinal variables [Internet] [Ph.D.]. ProQuest Dissertations and Theses. [United States -- Michigan]: Michigan State University; 2014. Available from: https://proxy.library.mcgill.ca/login?url=https://www.proquest.com/dissertations-theses/performance-mlr-uslmv-wlsmv-estimation-structural/docview/1538039469/se-2?accountid=12339

4. Li CH. Confirmatory factor analysis with ordinal data: Comparing robust maximum likelihood and diagonally weighted least squares. Behav Res. 2016 Sep 1;48(3):936–49.

# S1 Table. Scale Development Summary

| **Scale(s)** | **Subscales** | **Development** | |
| --- | --- | --- | --- |
|  |  | **Procedure** | **Participants** |
| **Compassionate Engagement and Action Scales:**   1. **Compassion for Self (CEAS-SC)** 2. **Compassion to others (CEAS-TO)** 3. **Compassion from others (CEAS-FROM)**   (Gilbert et al., 2017) | Each scale consists of:   1. Compassionate Engagement 2. Compassionate Action | Item generation: items generated based on theory (6 competencies related to engagement, 4 competencies related to action). | Research team |
|  |  | Exploratory factor analysis (EFA) used to separately analyze “engagement” and “action” subscales for each of the three orientations of compassion. EFA identified one factor for each of the six subscales, with the exception of “engagement” for self-compassion, which identified two factors. | British university students  (*N* = 278, 62.23% female) |
|  |  | Confirmatory factor analysis (CFA) used to test two-factor hierarchical models with three independent samples.  For CEAS-SC, a three-factor hierarchical model was fit based on EFA results. Model fit indices ranged from acceptable to good. Moderate correlations were observed between the three scales comprising the CEAS. | Portuguese university students  (*N* = 418, 86.12% female) |
|  |  |  | Portuguese community sample recruited through convenience sampling  (*N* = 344, 69.19% female) |
|  |  |  | American university students  (*N* = 312, 72.76% female) |

| **Compassion Scale (CS)**  (Pommier et al., 2019) | 1. Kindness 2. Mindfulness 3. Common humanity 4. Indifference (reverse-scored) | Item generation: items generated based on 6 proposed theoretical factors, inspected by researchers for content validity; 80 items retained. | Researchers (*N* = 6) and practitioners of Buddhist compassion practices (*N* = 2) |
| --- | --- | --- | --- |
|  |  | CFA and bifactor exploratory structural equation modeling (ESEM) used to examine a six-factor correlated model as well as a bifactor model (representing a general compassion factor as well as six components).  Negative components were not found to be empirically distinct using ESEM, and were thus collapsed into a single factor labelled “indifference.” | Student sample  (*N* = 465, 65% women) |
|  |  | Cross-validation using CFA and ESEM to examine four-factor correlated first-order and bifactor models. | Student sample  (*N*  = 510, 53% women) |
|  |  | Test-retest reliability over a one-month period. | Students (*N* = 80, 46% women) |
|  |  | Cross-validation using CFA and ESEM to examine four-factor correlated first-order and bifactor models with a community sample. | Community sample recruited from Mechanical Turk  (*N* = 1,394, 65% women) |
|  |  | Validation with a sample of individuals practicing Buddhist meditation. CFA and ESEM used to examine four-factor correlated first-order and bifactor models. | Meditators (*N* = 172, 72% women) |
|  |  | Examination of final version. | Community sample recruited from Mechanical Turk  (*N* = 913, 45% female) |
| **Sussex-Oxford Compassion Scales:**   1. **For Self (SOCS-S)** 2. **For Others (SOCS-O)**   (Gu et al., 2020) | Each scale consists of:   1. Recognizing suffering 2. Understanding the universality of human suffering 3. Feeling for the person suffering 4. Tolerating uncomfortable feelings 5. Motivation to act/acting to alleviate suffering | Item generation: items generated by interviewing teachers or researchers in the fields of mindfulness and compassion (including at least one teacher from each of six continents) and iterative review; 120 total items. | Experts in contemplative approaches  (*N* = 22, 72.7% female) |
|  |  | Item review: 120 items reduced to 115 items. | Experts (*N* = 15) and undergraduate students  (*N* = 15, 60% female) |
|  |  | Item reduction: CFA used to test proposed five-factor model; four highest loading items for each of the five proposed factors were retained (result: 20 items for each scale). | Health care workers  (*N* = 1,017, 79.6% female) |
|  |  | CFA to test five-factor hierarchical model. | Health care workers  (*N* = 1,319, 83.1% female) |
|  |  | Cross validation: CFA results support a five-factor hierarchical model. | Undergraduate students  (*N* = 371, 87.9% female) |

# S2 Table. Floor and Ceiling Effects

| **Item** | **Score** |
| --- | --- |
| CEAS-SC Engagement |  |
| CEAS_SC1_1 | 0.63 |
| CEAS_SC1_2 | 0.73 |
| CEAS_SC1_4 | 0.67 |
| CEAS_SC1_5 | 0.56 |
| CEAS_SC1_6 | 0.72 |
| CEAS_SC1_8 | 0.41 |
| CEAS-SC Action |  |
| CEAS_SC2_1 | 0.65 |
| CEAS_SC2_2 | 0.66 |
| CEAS_SC2_4 | 0.65 |
| CEAS_SC2_5 | 0.55 |
| CEAS-TO Engagement |  |
| CEAS_TO1_1 | 0.73 |
| CEAS_TO1_2 | 0.80 |
| CEAS_TO1_4 | 0.77 |
| CEAS_TO1_5 | 0.74 |
| CEAS_TO1_6 | 0.81 |
| CEAS_TO1_8 | 0.74 |
| CEAS-TO Action |  |
| CEAS_TO2_1 | 0.81 |
| CEAS_TO2_2 | 0.80 |
| CEAS_TO2_4 | 0.75 |
| CEAS_TO2_5 | 0.85 |
| CEAS-FROM Engagement |  |
| CEAS_FROM1_1 | 0.54 |
| CEAS_FROM1_2 | 0.52 |
| CEAS_FROM1_4 | 0.54 |
| CEAS_FROM1_5 | 0.56 |
| CEAS_FROM1_6 | 0.54 |
| CEAS_FROM1_8 | 0.58 |
| CEAS-FROM Action |  |
| CEAS_FROM2_1 | 0.62 |
| CEAS_FROM2_2 | 0.60 |
| CEAS_FROM2_4 | 0.55 |
| CEAS_FROM2_5 | 0.66 |
| CS |  |
| CS_1 | 0.87 |
| CS_2 | 0.87 |
| CS_3 | 0.89 |
| CS_4 | 0.93 |
| CS_5 | 0.77 |
| CS_6 | 0.82 |
| CS_7 | 0.64 |
| CS_8 | 0.93 |
| CS_9 | 0.84 |
| CS_10 | 0.77 |
| CS_11 | 0.66 |
| CS_12 | 0.83 |
| CS_13 | 0.82 |
| CS_14 | 0.84 |
| CS_15 | 0.80 |
| CS_16 | 0.92 |
| SOCS-S |  |
| SOCS_S_1 | 0.76 |
| SOCS_S_2 | 0.92 |
| SOCS_S_3 | 0.54 |
| SOCS_S_4 | 0.58 |
| SOCS_S_5 | 0.65 |
| SOCS_S_6 | 0.77 |
| SOCS_S_7 | 0.88 |
| SOCS_S_8 | 0.54 |
| SOCS_S_9 | 0.47 |
| SOCS_S_10 | 0.61 |
| SOCS_S_11 | 0.65 |
| SOCS_S_12 | 0.90 |
| SOCS_S_13 | 0.62 |
| SOCS_S_14 | 0.46 |
| SOCS_S_15 | 0.61 |
| SOCS_S_16 | 0.68 |
| SOCS_S_17 | 0.90 |
| SOCS_S_18 | 0.53 |
| SOCS_S_19 | 0.47 |
| SOCS_S_20 | 0.61 |
| SOCS-O |  |
| SOCS_O_1 | 0.72 |
| SOCS_O_2 | 0.92 |
| SOCS_O_3 | 0.83 |
| SOCS_O_4 | 0.81 |
| SOCS_O_5 | 0.80 |
| SOCS_O_6 | 0.74 |
| SOCS_O_7 | 0.92 |
| SOCS_O_8 | 0.83 |
| SOCS_O_9 | 0.80 |
| SOCS_O_10 | 0.80 |
| SOCS_O_11 | 0.71 |
| SOCS_O_12 | 0.92 |
| SOCS_O_13 | 0.83 |
| SOCS_O_14 | 0.75 |
| SOCS_O_15 | 0.80 |
| SOCS_O_16 | 0.72 |
| SOCS_O_17 | 0.92 |
| SOCS_O_18 | 0.84 |
| SOCS_O_19 | 0.67 |
| SOCS_O_20 | 0.79 |

*Note.* Compassionate Engagement and Action Scale – Self-Compassion (CEAS-SC); Compassionate Engagement and Action Scale – Compassion to Others (CEAS-TO); Compassionate Engagement and Action Scales – Compassion from Others (CEAS-FROM); Compassion Scale (CS); Sussex-Oxford Compassion Scale – Compassion for Self (SOCS-S); Sussex-Oxford Compassion Scale – Compassion for Others (SOCS-O). For CEAS-SC, CEAS-TO, and CEAS-FROM, items 3 and 7 within “engagement” and item 3 within “action” are not reported, as they are reversed filler items excluded from scoring.

# S3 Table. Pearson Correlation Coefficients adjusted by Benjamini-Hochberg Procedure.

|  | **1** | **2** | **3** | **4** | **5** | **6** | **7** | **8** | **9** | **10** | **11** | **12** | **13** | **14** | **15** | **16** | **17** | **18** | **19** | **20** | **21** | **22** | **23** | **24** | **25** | **26** | **27** |
| --- | --- | --- | --- | --- | --- | --- | --- | --- | --- | --- | --- | --- | --- | --- | --- | --- | --- | --- | --- | --- | --- | --- | --- | --- | --- | --- | --- |
| **1. CEAS-SC** | – |  |  |  |  |  |  |  |  |  |  |  |  |  |  |  |  |  |  |  |  |  |  |  |  |  |  |
| 2. Engagement Dimension 1 | 0.53*** | – |  |  |  |  |  |  |  |  |  |  |  |  |  |  |  |  |  |  |  |  |  |  |  |  |  |
| 3. Engagement Dimension 2 | 0.91*** | 0.38*** | – |  |  |  |  |  |  |  |  |  |  |  |  |  |  |  |  |  |  |  |  |  |  |  |  |
| 4. Engagement | 0.92*** | 0.7*** | 0.93*** | – |  |  |  |  |  |  |  |  |  |  |  |  |  |  |  |  |  |  |  |  |  |  |  |
| 5. Action | 0.91*** | 0.26*** | 0.74*** | 0.68*** | – |  |  |  |  |  |  |  |  |  |  |  |  |  |  |  |  |  |  |  |  |  |  |
| **6. CEAS-TO** | 0.26*** | 0.24*** | 0.2*** | 0.25*** | 0.21*** | – |  |  |  |  |  |  |  |  |  |  |  |  |  |  |  |  |  |  |  |  |  |
| 7. Engagement | 0.22*** | 0.25*** | 0.17** | 0.24*** | 0.15** | 0.96*** | – |  |  |  |  |  |  |  |  |  |  |  |  |  |  |  |  |  |  |  |  |
| 8. Action | 0.27*** | 0.18*** | 0.22*** | 0.24*** | 0.26*** | 0.9*** | 0.75*** | – |  |  |  |  |  |  |  |  |  |  |  |  |  |  |  |  |  |  |  |
| **9. CEAS-FROM** | 0.36*** | 0.14* | 0.31*** | 0.3*** | 0.37*** | 0.22*** | 0.18** | 0.25*** | – |  |  |  |  |  |  |  |  |  |  |  |  |  |  |  |  |  |  |
| 10. Engagement | 0.37*** | 0.14* | 0.32*** | 0.31*** | 0.35*** | 0.21*** | 0.17** | 0.23*** | 0.98*** | – |  |  |  |  |  |  |  |  |  |  |  |  |  |  |  |  |  |
| 11. Action | 0.34*** | 0.12* | 0.27*** | 0.26*** | 0.36*** | 0.23*** | 0.18** | 0.27*** | 0.96*** | 0.89*** | – |  |  |  |  |  |  |  |  |  |  |  |  |  |  |  |  |
| **12. CS** | 0.24*** | 0.26*** | 0.18** | 0.24*** | 0.19*** | 0.78*** | 0.76*** | 0.68*** | 0.13* | 0.12* | 0.14* | – |  |  |  |  |  |  |  |  |  |  |  |  |  |  |  |
| 13. Common Humanity | 0.19*** | 0.19*** | 0.14** | 0.19*** | 0.16** | 0.39*** | 0.38*** | 0.35*** | 0.09 | 0.09 | 0.08 | 0.62*** | – |  |  |  |  |  |  |  |  |  |  |  |  |  |  |
| 14. Indifference (Reverse) | 0.09 | 0.13* | 0.07 | 0.11* | 0.05 | 0.61*** | 0.62*** | 0.51*** | 0.09 | 0.09 | 0.09 | 0.79*** | 0.27*** | – |  |  |  |  |  |  |  |  |  |  |  |  |  |
| 15. Kindness | 0.18** | 0.24*** | 0.1 | 0.17** | 0.15** | 0.71*** | 0.67*** | 0.65*** | 0.14* | 0.11* | 0.16** | 0.86*** | 0.37*** | 0.6*** | – |  |  |  |  |  |  |  |  |  |  |  |  |
| 16. Mindfulness | 0.3*** | 0.23*** | 0.24*** | 0.28*** | 0.26*** | 0.63*** | 0.62*** | 0.56*** | 0.09 | 0.07 | 0.11 | 0.79*** | 0.4*** | 0.43*** | 0.65*** | – |  |  |  |  |  |  |  |  |  |  |  |
| **17. SOCS-S** | 0.78*** | 0.36*** | 0.73*** | 0.71*** | 0.72*** | 0.22*** | 0.18** | 0.24*** | 0.34*** | 0.35*** | 0.3*** | 0.26*** | 0.26*** | 0.26*** | 0.26*** | 0.26*** | – |  |  |  |  |  |  |  |  |  |  |
| 18. Acting/motivation to act to alleviate suffering | 0.73*** | 0.26*** | 0.67*** | 0.62*** | 0.72*** | 0.13* | 0.1 | 0.14** | 0.34*** | 0.34*** | 0.32*** | 0.16** | 0.16** | 0.16** | 0.16** | 0.16** | 0.84*** | – |  |  |  |  |  |  |  |  |  |
| 19. Feeling suffering | 0.75*** | 0.31*** | 0.7*** | 0.67*** | 0.7*** | 0.12* | 0.09 | 0.15** | 0.33*** | 0.34*** | 0.29*** | 0.14** | 0.14** | 0.14** | 0.14** | 0.14** | 0.89*** | 0.76*** | – |  |  |  |  |  |  |  |  |
| 20. Recognizing Suffering | 0.47*** | 0.38*** | 0.41*** | 0.47*** | 0.39*** | 0.17** | 0.14* | 0.19*** | 0.18** | 0.17** | 0.18** | 0.2*** | 0.2*** | 0.2*** | 0.2*** | 0.2*** | 0.71*** | 0.44*** | 0.49*** | – |  |  |  |  |  |  |  |
| 21. Tolerating uncomfortable feelings | 0.71*** | 0.25*** | 0.7*** | 0.65*** | 0.65*** | 0.1 | 0.06 | 0.14* | 0.28*** | 0.3*** | 0.24*** | 0.12* | 0.12* | 0.12* | 0.12* | 0.12* | 0.87*** | 0.67*** | 0.79*** | 0.51*** | – |  |  |  |  |  |  |
| 22. Understanding universality of suffering | 0.23*** | 0.19*** | 0.2*** | 0.23*** | 0.18*** | 0.37*** | 0.34*** | 0.35*** | 0.14* | 0.16** | 0.09 | 0.48*** | 0.48*** | 0.48*** | 0.48*** | 0.48*** | 0.43*** | 0.21*** | 0.19*** | 0.21*** | 0.21*** | – |  |  |  |  |  |
| **23. SOCS-O** | 0.24*** | 0.18*** | 0.18*** | 0.21*** | 0.21*** | 0.76*** | 0.72*** | 0.7*** | 0.19*** | 0.18** | 0.2*** | 0.77*** | 0.77*** | 0.77*** | 0.77*** | 0.77*** | 0.28*** | 0.17*** | 0.13* | 0.26*** | 0.1* | 0.54*** | – |  |  |  |  |
| 24. Acting/motivation to act to alleviate suffering | 0.18*** | 0.1 | 0.12* | 0.13* | 0.19*** | 0.67*** | 0.61*** | 0.66*** | 0.25*** | 0.23*** | 0.26*** | 0.61*** | 0.61*** | 0.61*** | 0.61*** | 0.61*** | 0.19*** | 0.16** | 0.07 | 0.16** | 0.04 | 0.33*** | 0.84*** | – |  |  |  |
| 25 Feeling suffering | 0.16** | 0.18*** | 0.11 | 0.15** | 0.12* | 0.71*** | 0.68*** | 0.63*** | 0.19*** | 0.18** | 0.2*** | 0.71*** | 0.71*** | 0.71*** | 0.71*** | 0.71*** | 0.15** | 0.1* | 0.05 | 0.16** | -0.03 | 0.35*** | 0.86*** | 0.74*** | – |  |  |
| 26. Recognizing Suffering | 0.15** | 0.11* | 0.1 | 0.13* | 0.13* | 0.49*** | 0.47*** | 0.44*** | 0 | -0.01 | 0.03 | 0.54*** | 0.54*** | 0.54*** | 0.54*** | 0.54*** | 0.18*** | 0.05 | 0.09 | 0.25*** | 0.07 | 0.23*** | 0.73*** | 0.45*** | 0.53*** | – |  |
| 27. Tolerating uncomfortable feelings | 0.24*** | 0.13* | 0.23*** | 0.23*** | 0.2*** | 0.66*** | 0.63*** | 0.6*** | 0.16** | 0.15** | 0.16** | 0.62*** | 0.62*** | 0.62*** | 0.62*** | 0.62*** | 0.28*** | 0.16** | 0.14** | 0.22*** | 0.19*** | 0.41*** | 0.83*** | 0.64*** | 0.65*** | 0.48*** | – |
| 28. Understanding universality of suffering | 0.22*** | 0.24*** | 0.16** | 0.22*** | 0.18** | 0.41*** | 0.4*** | 0.37*** | 0.14* | 0.14* | 0.12* | 0.55*** | 0.55*** | 0.55*** | 0.55*** | 0.55*** | 0.34*** | 0.19*** | 0.14** | 0.18*** | 0.11* | 0.83*** | 0.63*** | 0.38*** | 0.45*** | 0.27*** | 0.44*** |
| **29. SCS-Positive** | 0.77*** | 0.25*** | 0.75*** | 0.69*** | 0.74*** | 0.09 | 0.06 | 0.13* | 0.28*** | 0.3*** | 0.24*** | 0.15** | 0.15** | 0.15** | 0.15** | 0.15** | 0.72*** | 0.66*** | 0.71*** | 0.35*** | 0.7*** | 0.22*** | 0.14* | 0.08 | 0.04 | 0.09 | 0.16** |
| **30. SCS-Negative** | -0.6*** | -0.06 | -0.6*** | -0.49*** | -0.62*** | -0.05 | -0.01 | -0.1 | -0.3*** | -0.32*** | -0.24*** | -0.06 | -0.06 | -0.06 | -0.06 | -0.06 | -0.59*** | -0.51*** | -0.59*** | -0.25*** | -0.64*** | -0.14** | -0.08 | -0.09 | 0.01 | 0 | -0.16** |
| **31. DASS-21** | -0.55*** | -0.09 | -0.52*** | -0.44*** | -0.57*** | -0.01 | 0.03 | -0.08 | -0.39*** | -0.4*** | -0.35*** | -0.02 | -0.02 | -0.02 | -0.02 | -0.02 | -0.53*** | -0.52*** | -0.51*** | -0.22*** | -0.52*** | -0.18** | -0.06 | -0.08 | -0.03 | 0.08 | -0.09 |
| **32. ECR-Anx** | -0.30*** | 0.06 | -0.32*** | -0.22*** | -0.34*** | 0.02 | 0.04 | -0.01 | -0.13* | -0.14* | -0.12* | 0.05 | 0.00 | 0.06 | 0.08 | 0 | -0.33*** | -0.29*** | -0.33*** | -0.14* | -0.32*** | -0.13* | -0.02 | -0.04 | 0.03 | 0.07 | -0.08 |
| **33. ECR-Avoid** | -0.28*** | -0.12* | -0.26*** | -0.25*** | -0.27*** | -0.16** | -0.13* | -0.18** | -0.41*** | -0.40*** | -0.39*** | -0.13* | -0.13* | -0.10 | -0.08 | -0.10 | -0.35*** | -0.32*** | -0.32*** | -0.27*** | -0.26*** | -0.14* | -0.16** | -0.16** | -0.10 | -0.12* | -0.13* |
| **34. FFMQ** | 0.7*** | 0.26*** | 0.68*** | 0.63*** | 0.64*** | 0.13* | 0.12* | 0.14* | 0.21*** | 0.22*** | 0.19** | 0.17** | 0.17** | 0.17** | 0.17** | 0.17** | 0.7*** | 0.56*** | 0.63*** | 0.49*** | 0.7*** | 0.24*** | 0.17** | 0.08 | 0.06 | 0.15* | 0.22*** |
| **35. WEMWBS** | 0.57*** | 0.13* | 0.49*** | 0.43*** | 0.62*** | 0.21*** | 0.15** | 0.26*** | 0.39*** | 0.38*** | 0.38*** | 0.21*** | 0.21*** | 0.21*** | 0.21*** | 0.21*** | 0.61*** | 0.56*** | 0.57*** | 0.35*** | 0.57*** | 0.21*** | 0.24*** | 0.22*** | 0.14** | 0.21*** | 0.21*** |

*Note.* Compassionate Engagement and Action Scale – Self-Compassion (CEAS-SC); Compassionate Engagement and Action Scale – Compassion to Others (CEAS-TO); Compassionate Engagement and Action Scales – Compassion from Others (CEAS-FROM); Compassion Scale (CS); Sussex-Oxford Compassion Scale – Compassion for Self (SOCS-S); Sussex-Oxford Compassion Scale – Compassion for Others (SOCS-O); Self-Compassion Scale – Positive Subscales (SCS-POS); Self-Compassion Scale – Negative Subscales (SCS-NEG); Depression, Anxiety, and Stress Scale – 21 (DASS-21); Experiences in Close Relationships (ECR); Five Facet Mindfulness Questionnaire (FFMQ); Warwick Edinburgh Mental Wellbeing Scale (WEMBS).
The Benjamini-Hochberg procedure was performed to control the false discovery rate.
** p < 0.05, ** p < 0.01, *** p < 0.001*

# S1 Fig. CEAS-SC, One-Factor Model.


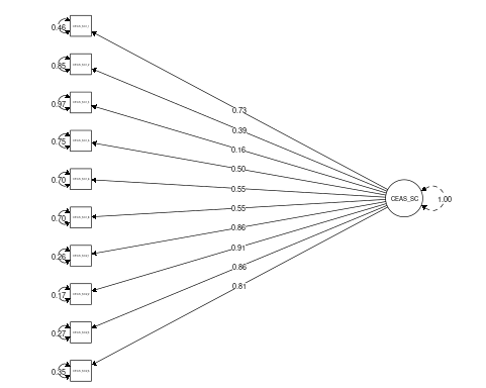


# S2 Fig. CEAS-SC, Two-Factor Model.


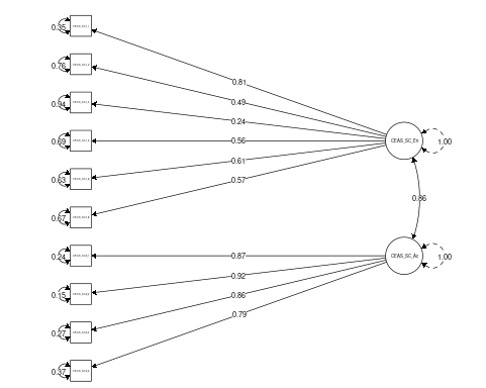


# S3 Fig. CEAS-SC, Two-Factor Hierarchical Model.


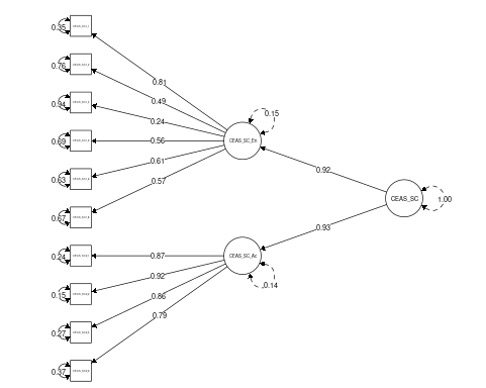


# S4 Fig. CEAS-SC, Three-Factor Model.


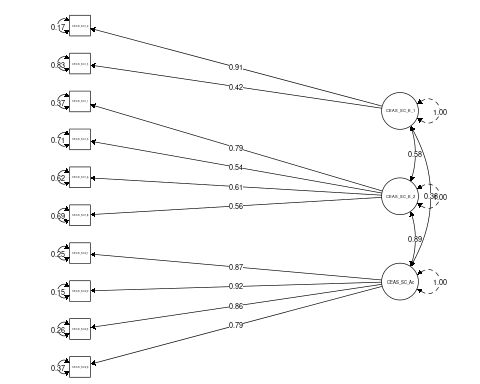


# S5 Fig. CEAS-TO, One-Factor Model.


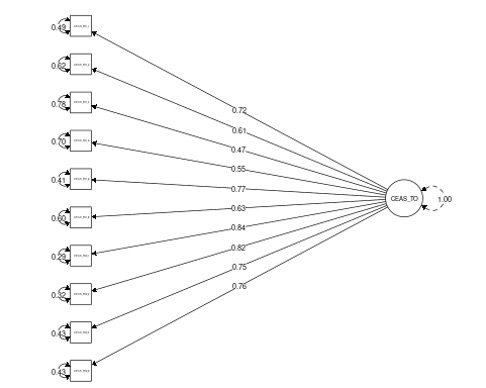


# S6 Fig. CEAS-TO, Two-Factor Model.


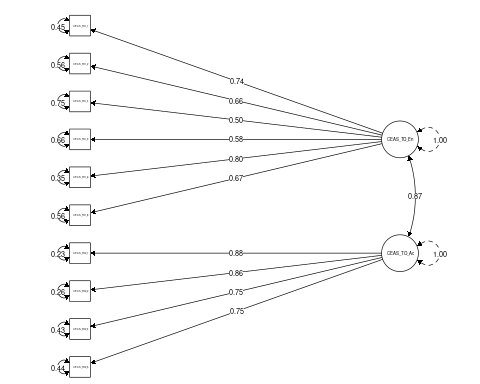


# S7 Fig. CEAS-FROM, One-Factor Model.

**
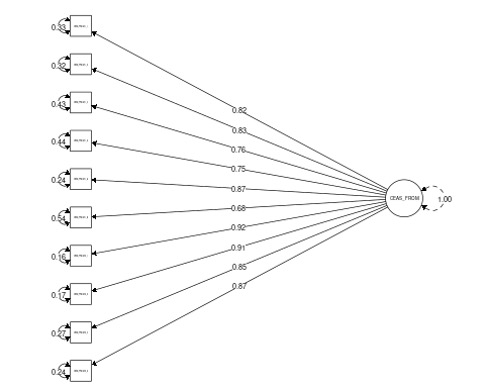
**

# S8 Fig. CEAS-FROM, Two-Factor Model.


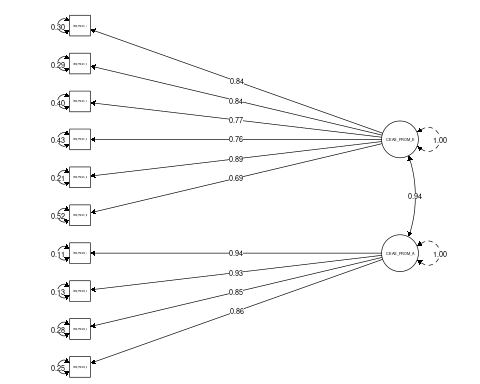


# S9 Fig. CEAS-FROM, Two-Factor Hierarchical Model.


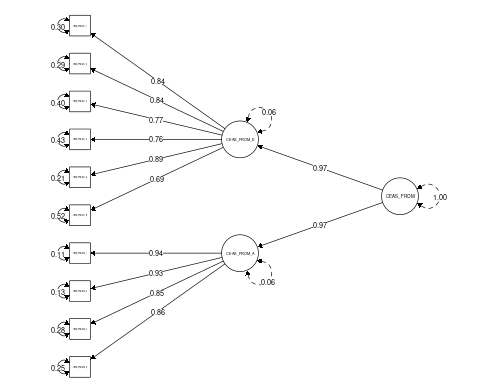


# S10 Fig. CS, One-Factor Model.

*
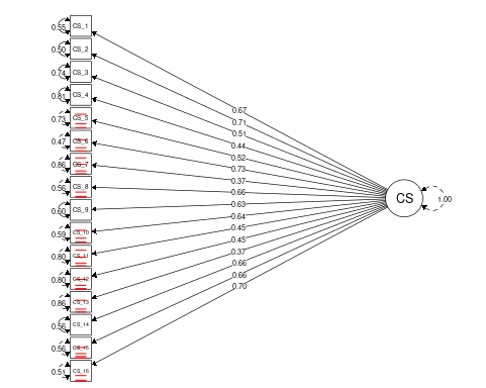
*

# S11 Fig. CS, Four-Factor Model.


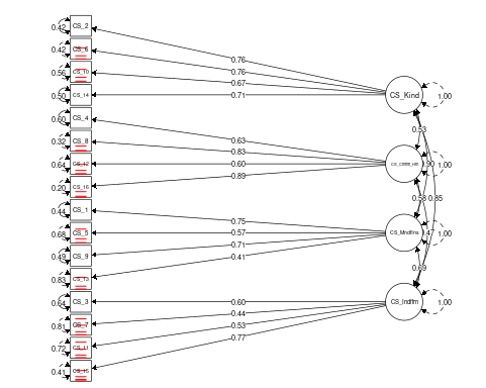


# S12 Fig. CS Four-Factor Hierarchical Model.


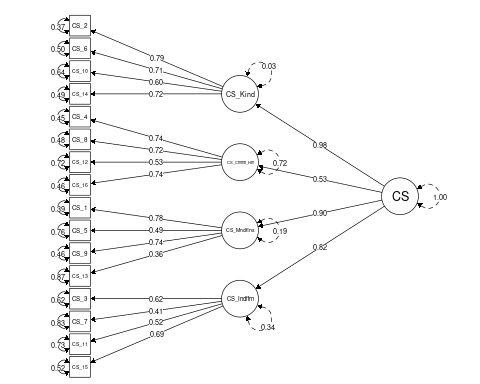


# S13 Fig. SOCS-S, One-Factor Model.

*
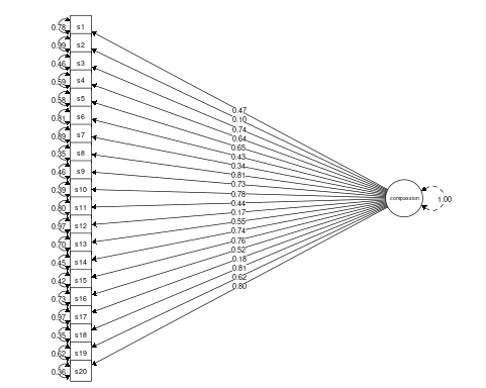
*

# S14 Fig. SOCS-S Five-Factor Model.


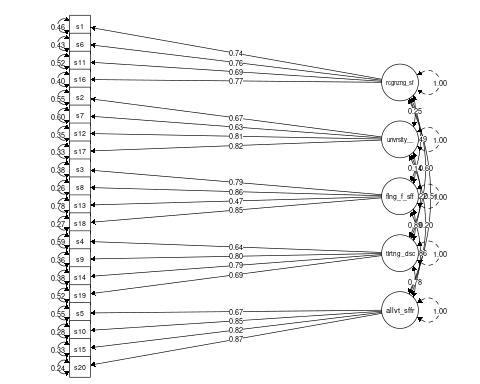


# S15 Fig. SOCS-S, Five-Factor Hierarchical Model.


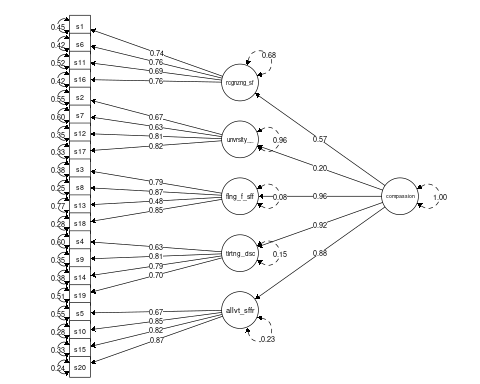


# S16 Fig. SOCS-O, One-Factor Model.

*
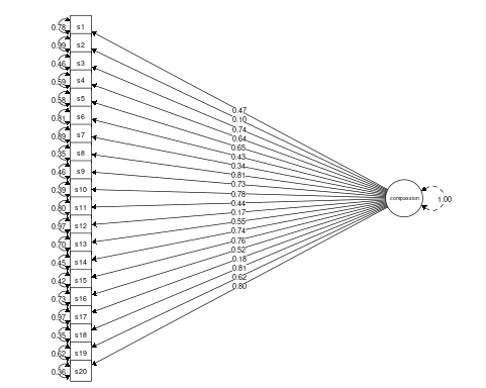
*

# S17 Fig. SOCS-O, Four-Factor Model.


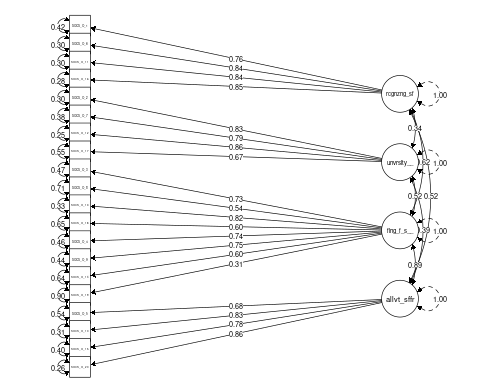


# S18 Fig. SOCS-O, Five-Factor Hierarchical Model.


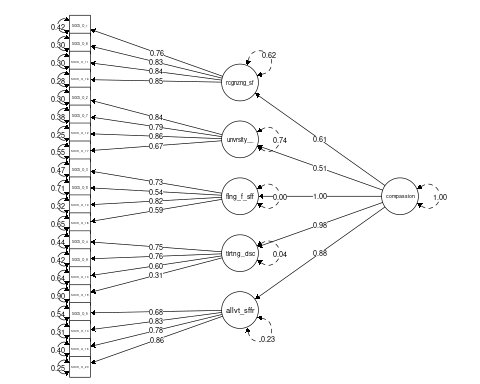

Supplement: S2 Appendix — (DOCX) [file pone.0305776.s002.docx]
