# Supplementary material for: Validation of the compassionate engagement and action scales, compassion scale, and Sussex-Oxford compassion scales in a French-Canadian sample
Source: PLoS One. 2024 Jun 24;19(6):e0305776. doi: 10.1371/journal.pone.0305776 (PMC11195958; doi:10.1371/journal.pone.0305776)
Supplement: S3 Appendix — French translations of the Compassionate Engagement and Action Scales, Compassion Scale, and Sussex-Oxford Compassion Scales for Self and Others. (ZIP) [file pone.0305776.s003.zip › S2 Translation Reports/SOCS.Translation Report.pdf]

**Sussex-Oxford Compassion for Others Scale (SOCS-O)**

***Questionnaire de compassion pour les autres (Sussex-Oxford Compassion for Others Scale - SOCS-O)***

| **English Original** | **French Translation**<br><br>*Independently translated (English to French) by two native French speakers, and synthesized during a consensus meeting.* | **Back Translation 1** | **Back Translation 2** | **French Translation after Back Translation**<br><br>*Where applicable, French items were revised following a committee meeting consisting of translators, back-translators, PI, and co-investigators.* |
|---|---|---|---|---|
| Below are statements describing how you might relate to other people. Please indicate how true the following statements are of you using the 5-point response scale (1 = Not at all true, 2 = Rarely true, 3 = Sometimes true, 4 = Often true, 5 = Always true). For example, if you think that a statement is often true of you, circle '4'.<br><br><u>Note</u>: In the below items, generic terms (e.g., 'upset', 'distress', 'suffering', struggling') are used to cover a range of unpleasant emotions, such as sadness, fear, anger, frustration, guilt, shame, etc.<br><br>Please provide an answer for each statement. | Vous trouverez ci-dessous des énoncés décrivant comment vous pouvez vous identifier à <u>**d'autres personnes**</u> et les comprendre. Merci d'indiquer à quel point les affirmations suivantes sont vraies pour vous en utilisant l'échelle en 5 points (1=pas du tout vrai ; 2=rarement vrai ; 3=parfois vrai ; 4=souvent vrai ; 5=toujours vrai). Par exemple, si vous pensez qu'une affirmation est souvent vraie pour vous, vous entourez le chiffre « 4 ».<br><br>Note : dans les énoncés ci-dessous, des termes génériques (p.ex : « contrarié(e) », « bouleversé(e) », « en souffrance », « en difficulté », etc.) sont utilisés pour couvrir un large éventail d'émotions désagréables comme la tristesse, la peur, la colère, la frustration, la culpabilité, la honte, etc. | You will find below statements describing ways in which you identify <u>**with other people**</u> and understand <u>**them**</u>. Please indicate the degree to which each statement is true for you using the 5-point scale (1 = not at all true; 2 = rarely true; 3 = sometimes true; 4 = often true; 5 = always true). For example, if you think a statement below is often true for you, circle the number 4.<br><br>Note: the statements below make use of generic terms (e.g. "upset", "overwhelmed", "suffering", "struggling", etc.) that are meant to cover a wide range of unpleasant emotions, such as sadness, fear, anger, frustration, guilt, shame, etc.<br><br>Please provide an answer for each item. | Below you will find statements describing how you can relate to <u>**other people**</u>. Please indicate using the 5-point scale to which degree the following assertions are true for you (1=not at all true ; 2=rarely true; 3=sometimes true ; 4=often true; 5=always true). For example, if you think that an assertion is often true for you, circle the number "4."<br><br>Note : in the following statements, general terms (ex. "upset," "distressed," "suffering," difficulties" :etc.) are used to cover a large variety of unpleasant feelings like sadness, fear, anger, frustration, guilt, shame, etc.<br><br>Please give an answer for each statement. | |

| | | | | |
|---|---|---|---|---|
| | Veuillez fournir une réponse pour chaque énoncé. | | | |
| 1. I recognise when other people are feeling distressed without them having to tell me. | 1. J'arrive bien à reconnaître quand les autres se sentent en détresse sans qu'ils/elles aient à me le dire | 1. I'm able to recognize when others are distraught without them having to tell me. | 1. I am good at noticing when others are in distress without them needing to tell me. | Je reconnais lorsque les autres se sentent en détresse sans qu'ils aient à me le dire |
| 2. I understand that everyone experiences suffering at some point in their lives. | 2. Je sais que tout le monde souffre à certains moments de sa vie | 2. I know that everyone suffers at certain points in life. | 2. I know that everyone experiences suffering at some point in their life. | Je comprends que tout le monde souffre à certains moments de sa vie |
| 3. When someone is going through a difficult time, I feel kindly towards them. | 3. Lorsque quelqu'un vit une passe difficile, j'agis de façon bienveillante avec elle/lui | 3. When others are going through a difficult time, I treat them with kindness. | 3. When someone is going through a difficult time, I am caring with them. | |
| 4. When someone else is upset, I try to stay open to their feelings rather than avoid them. | 4. Lorsque quelqu'un est bouleversé/contrarié, j'essaie de rester ouvert(e) à ses sentiments plutôt que de les éviter | 4. When others are overwhelmed or upset, I try to accept their feelings rather than avoid them. | 4. When someone is distressed/upset, I try to remain open to their feelings rather than avoiding them. | |
| 5. When others are struggling, I try to do things that would be helpful. | 5. Lorsque d'autres personnes rencontrent des difficultés, j'essaie de faire des choses qui pourraient être utiles | 5. When others are struggling, I try to do things that might be helpful to them. | 5. When someone is experiencing difficulties, I try to do things that could be helpful. | |
| 6. I notice when others are feeling distressed. | 6. Je remarque quand d'autres personnes se sentent en détresse | 6. I notice when others are feeling distraught. | 6. I notice when others are in distress. | |
| 7. I understand that feeling upset at times is part of human nature. | 7. Je sais que se sentir bouleversé(e)/ contrarié(e) par moments fait partie de la nature humaine | 7. I know that feeling overwhelmed or upset at times is part of human nature. | 7. I know that feeling distressed/upset at times is part of the human experience. | |
| 8. When I hear about bad things happening to other people, I feel concern for their wellbeing. | 8. Lorsque j'apprends que de mauvaises choses arrivent à d'autres, je suis inquiet(ète) pour leur bien être | 8. When I learn that difficult things have happened to other people, I worry for their wellbeing. | 8. When I learn that bad things happen to others, I am concerned for their wellbeing | |
| 9. I stay with and listen to other people when they're upset even if | 9. Lorsque d'autres personnes sont contrarié(e)s/bouleversé(e)s, je | 9. When other people are overwhelmed or upset, I stay | 9. When others are upset/distressed, I stay with | |

**Commented [A1]:** The phrasing 'J'arrive bien' was replaced by 'Je reconnais' to be closer to the original 'I recognize' rather than the 'ability to recognize' identified in the back translation.

**Commented [A2]:** The term 'Je sais' was replaced with 'Je comprends' to more accurately/appropriately reflect the nuance of *knowing* something versus *understanding* something.

| | | | | |
|---|---|---|---|---|
| it's hard to bear. | reste avec elles et je les écoute, même si c'est difficile | with them and listen to them, even when it's difficult. | and listen to them, even if it's difficult. | |
| 10. When someone is going through a difficult time, I try to look after them. | 10. Quand quelqu'un traverse une période difficile, j'essaie de prendre soin d'elle/lui | 10. When someone is struggling or going through a difficult time, I try to take care of them. | 10. When someone is going through a difficult time, I try to take care of them. | |
| 11. I'm quick to notice early signs of distress in others. | 11. Je repère rapidement les premiers signes de détresse chez les autres | 11. I quickly identify signs of distress in others. | 11. I quickly recognize the first signs of distress in others. | |
| 12. Like me, I know that other people also experience struggles in life. | 12. Je sais que, comme moi, d'autres personnes rencontrent aussi des difficultés dans leur vie | 12. I know that other people experience difficulty in their lives, just like I do. | 12. I know that, just like I do, others experience difficulties in their life. | |
| 13. When someone is upset, I try to tune in to how they're feeling. | 13. Lorsque quelqu'un est bouleversé~~(e)~~/ contrarié~~(e)~~, j'essaie d'être à l'écoute de ce qu'ils ressentent | 13. When someone is overwhelmed or upset, I try to listen attentively to what they are feeling. | 13. When someone is distressed/upset, I try to pay attention to what they're feeling. | |
| 14. I connect with the suffering of others without judging them. | 14. Je me connecte avec la souffrance des autres sans les juger | 14. I am attuned to others' suffering without judging them. | 14. I engage with others' suffering without judging them. | |
| 15. When I see someone in need, I try to do what's best for them. | 15. Quand je vois quelqu'un qui a besoin d'aide, j'essaie de faire ce qu'il y a de mieux pour elle/lui | 15. When I see that someone needs help, I try to do what's best for them. | 15. When I see that someone is distressed/upset, I try to do what's best for them. | |
| 16. I recognise signs of suffering in others. | 16. J'identifie les signes de souffrance chez les autres | 16. I identify signs of suffering in others. | 16. I recognize signs of suffering in others. | |
| 17. I know that we can all feel upset at times when we are wronged. | 17. Je sais que nous pouvons tous nous sentir parfois bouleversés/contrariés lorsque quelqu'un nous fait du tort | 17. I know that we can all feel overwhelmed or upset when someone does something that hurts us. | 17. I know that we can all feel distressed/upset when someone hurts us. | Je sais que nous pouvons tous et toutes nous sentir bouleversé(e)s/contrarié(e)s lorsqu'on nous fait du tort. |
| 18. I'm sensitive to other people's distress. | 18. Je suis sensible à la détresse des autres | 18. I am sensitive to others' feelings of distress. | 18. I am sensitive to others' distress. | |
| 19. When someone else is upset, I can be there for them without feeling | 19. Quand quelqu'un est bouleversé(e)/contrarié(e), je peux être présent(e) pour elle/lui | 19. When someone is overwhelmed or upset, I can be present for them | 19. When others are distressed/upset, I can be there for them without | |

**Commented [A3]:** The phrasing was revised to remove the personal aspect of *someone* wronging us that emerged in the French translation. The revised version is closer to the original as it keeps the 'wrong doing,' but removes the specific person/wrongdoer.

| | | | | |
|---|---|---|---|---|
| overwhelmed by their distress. | sans être submergé(e) par sa détresse | without feeling overcome by their distress. | being overwhelmed by their distress. | |
| 20. When I see that someone is upset, I do my best to take care of them. | 20. Quand je vois quelqu'un qui est bouleversé/contrarié, je fais de mon mieux pour prendre soin d'elle/lui | 20. When I see that someone is overwhelmed or upset, I try my best to take care of them. | 20. When see someone who is distressed/upset, I do my best to take care of them. | |
| **Scoring Information**<br><br>Users may compute both total subscale scores and a total SOCS-O score. To compute total subscale scores, sum item scores within each subscale (see below for items associated with each subscale). To compute a total SOCS-O score, sum all five total subscale scores or sum all 20 item scores.<br><br>Recognising suffering items: 1, 6, 11, 16.<br>Understanding the universality of suffering items: 2, 7, 12, 17.<br>Feeling for the person suffering items: 3, 8, 13, 18.<br>Tolerating uncomfortable feelings items: 4, 9, 14, 19.<br>Acting or being motivated to act to alleviate suffering items: 5, 10, 15, 20. | **Guide de notation**<br><br>Les utilisateurs peuvent calculer à la fois les scores totaux des sous-échelles et le score total SOCS-O. Pour calculer les scores totaux des sous-échelles, additionnez les scores de chaque item, et ce pour chaque sous-échelle (voir ci-dessous les items associés à chaque sous-échelle). Pour calculer le score total SOCS-O, additionnez le score total des cinq sous-échelles ou additionnez le score des 20 items.<br><br>Items liés à la reconnaissance de la souffrance : 1, 6, 11, 16.<br><br>Items liés à la compréhension de l'universalité de la souffrance : 2, 7, 12, 17.<br><br>Items liés à la sensibilité envers la personne qui souffre : 3, 8, 13, 18.<br><br>Items liés à la tolérance face aux sentiments inconfortables : 4, 9, 14, 19.<br><br>Items liés à l'action ou la motivation à agir pour soulager la souffrance : 5, 10, 15, 20. | Items assessing the ability to recognize suffering: 1, 6, 11, 16.<br><br>Items assessing one's understanding of the universality of suffering: 2, 7, 12, 17<br><br>Items assessing sensitivity toward the person who is suffering: 3, 8, 13, 18.<br><br>Items assessing tolerance for uncomfortable feelings: 4, 9, 14, 19<br><br>Items assessing actions or motivation to act to attenuate suffering: 5, 10, 15, 20. | Items related to recognizing suffering: 1, 6, 11, 16.<br><br>Items related to understanding the universality of suffering: 2, 7, 12, 17.<br><br>Items related to feeling for the person suffering: 3, 8, 13, 18.<br><br>Items related to tolerance of uncomfortable feelings: 4, 9, 14, 19.<br><br>Items related to acting or motivation to act to alleviate suffering: 5, 10, 15, 20. | |

**Sussex-Oxford Compassion for the Self Scale (SOCS-S)**

***Questionnaire de compassion pour soi (Sussex-Oxford Compassion for Self Scale - SOCS-S)***

| **English Original** | **French Translation**<br><br>*Independently translated (English to French) by two native French speakers, and synthesized during a consensus meeting.* | **Back Translation 1** | **Back Translation 2** | **French Translation after Back Translation**<br><br>*Where applicable, French items were revised following a committee meeting consisting of translators, back-translators, PI, and co-investigators.* |
|---|---|---|---|---|
| **Instructions**<br><br>Below are statements describing how you might relate to yourself. Please indicate how true the following statements are of you using the 5-point response scale (1 = Not at all true, 2 = Rarely true, 3 = Sometimes true, 4 = Often true, 5 = Always true). For example, if you think that a statement is often true of you, circle '4'.<br><br>Note: In the below items, generic terms (e.g., 'upset', 'distress', 'suffering', struggling') are used to cover a range of unpleasant emotions, such as sadness, fear, anger, frustration, guilt, shame, etc.<br><br>Please provide an answer for each statement. | Vous trouverez ci-dessous des énoncés décrivant comment vous pouvez vous comprendre **vous-même**. Merci d'indiquer à quel point les affirmations suivantes sont vraies pour vous en utilisant l'échelle en 5 points (1=pas du tout vrai ; 2=rarement vrai ; 3=parfois vrai ; 4=souvent vrai ; 5=toujours vrai). Par exemple, si vous pensez qu'une affirmation est souvent vraie pour vous, vous entourez le chiffre « 4 ».<br><br>Note : dans les énoncés ci-dessous, des termes génériques (p.ex : « contrarié(e) », « bouleversé(e) », « en souffrance », « en difficulté », etc.) sont utilisés pour couvrir un large éventail d'émotions désagréables comme la tristesse, la peur, la colère, la frustration, la culpabilité, la honte, etc. | You will find below statements describing how **you understand yourself**. Please indicate the degree to which each statement is true for you using the 5-point scale (1 = not at all true; 2 = rarely true; 3 = sometimes true; 4 = often true; 5 = always true). For example, if you think a statement below is often true for you, circle the number 4.<br><br>Note: the statements below make use of generic terms (e.g. "upset", "overwhelmed", "suffering", "struggling", etc.) that are meant to cover a wide range of unpleasant emotions, such as sadness, fear, anger, frustration, guilt, shame, etc.<br><br>Please provide an answer for each item. | Below you will find statements describing how you can understand **yourself**. Please indicate using the 5-point scale to which degree the following assertions are true for you (1=not at all true ; 2=rarely true; 3=sometimes true ; 4=often true; 5=always true). For example, if you think that an assertion is often true for you, circle the number "4."<br><br>Note : in the following statements, general terms (ex. "upset," "distressed," "suffering," difficulties" :etc.) are used to cover a large variety of unpleasant feelings like sadness, fear, anger, frustration, guilt, shame, etc.<br><br>Please give an answer for each statement. | |

| | | | | |
|---|---|---|---|---|
| | Veuillez fournir une réponse pour chaque énoncé. | | | |
| 1. I'm good at recognising when I'm feeling distressed. | 1. J'arrive bien à reconnaître quand je me sens en détresse | 1. I am able to recognize when I am feeling distraught. | 1. I am good at noticing when I am distressed. | |
| 2. I understand that everyone experiences suffering at some point in their lives. | 2. Je sais que tout le monde souffre à certains moments de sa vie | 2. I know that everyone suffers at certain points in life. | 2. I know that everyone experiences suffering at some point in their life. | Je comprends que tout le monde souffre à certains moments de sa vie. |
| 3. When I'm going through a difficult time, I feel kindly towards myself. | 3. Lorsque je vis une passe difficile, j'agis de façon bienveillante avec moi-même | 3. When I'm going through a difficult time, I treat myself with kindness. | 3. When I am going through a difficult time, I am caring with myself. | |
| 4. When I'm upset, I try to stay open to my feelings rather than avoid them. | 4. Lorsque je suis bouleversé(e)/contrarié(e), j'essaie de rester ouvert(e) à mes sentiments plutôt que de les éviter | 4. When I am overwhelmed or upset, I try to accept my feelings rather than avoid them. | 4. When I am distressed/upset, I try to remain open to my feelings rather than avoiding them. | |
| 5. I try to make myself feel better when I'm distressed, even if I can't do anything about the cause. | 5. J'essaie de faire en sorte de me sentir mieux lorsque je suis en détresse même si je ne peux pas agir sur la cause | 5. I try to do things that make me feel better when I'm distraught, even if I can't directly address the cause of the distress. | 5. I try to do something to feel better when I am in distress, even if I can't change the cause of it. | |
| 6. I notice when I'm feeling distressed. | 6. Je le remarque quand je me sens en détresse | 6. I notice when I am experiencing distress. | 6. I notice when I am in distress. | |
| 7. I understand that feeling upset at times is part of human nature. | 7. Je sais que se sentir bouleversé(e)/contrarié(e) par moments fait partie de la nature humaine | 7. I know that feeling overwhelmed or upset at times is part of human nature. | 7. I know that feeling distressed/upset at times is part of the human experience. | |
| 8. When bad things happen to me, I feel caring towards myself. | 8. Lorsque des choses difficiles m'arrivent, je suis attentionné(e) envers moi-même | 8. When difficult things happen to me, I am considerate toward myself. | 8. When bad things happen to me, I am considerate towards myself. | Lorsque des choses difficiles m'arrivent, je me sens bienveillant(e) envers moi-même. |
| 9. I connect with my own distress without letting it overwhelm me. | 9. J'entre en contact avec ma propre détresse sans me laisser envahir par elle | 9. I am able to experience my own feelings of distress without being entirely overcome by them. | 9. I engage with my distress without letting myself become overwhelmed by it. | |

**Commented [A4]:** [Same as previous scale] The term 'Je sais' was replaced with 'Je comprends' to more accurately/appropriately reflect the nuance of *knowing* something versus *understanding* something.

**Commented [A5]:** The term 'je suis attentioné(e)' was changed to 'je me sens bienveillant' to more closely reflect the term 'I feel caring' in the original. The back-translations could be interpreted as action-oriented, whereas this item should load onto the subscale measuring *feeling* caring (rather than acting/being motivated to act).

| | | | | |
|---|---|---|---|---|
| 10. When I'm going through a difficult time, I try to look after myself. | 10. Quand je traverse une période difficile, j'essaie de prendre soin de moi | 10. When I am struggling or going through a difficult time, I try to take care of myself. | 10. When I am going through a difficult time, I try to take care of myself. | |
| 11. I'm quick to notice early signs of distress in myself. | 11. Je repère rapidement en moi les premiers signes de détresse | 11. I can quickly identify the first signs of distress in myself. | 11. I quickly recognize the first signs of distress in myself. | |
| 12. Like me, I know that other people also experience struggles in life. | 12. Je sais que, comme moi, d'autres personnes font aussi l'expérience de difficultés dans leur vie | 12. I know that other people struggle and go through difficult moments in their life, like I do. | 12. I know that, just like I do, others experience difficulties in their life. | |
| 13. When I'm upset, I try to tune in to how I'm feeling. | 13. Lorsque je suis bouleversé(e)/contrarié(e), j'essaie de me centrer sur ce que je ressens | 13. When I am overwhelmed or upset, I try to focus on what I am feeling. | 13. When I am distressed/upset, I try to concentrate on what I'm feeling. | Lorsque je suis bouleversé(e)/contrarié(e), j'entre en contact avec ce que je ressens. |
| 14. I connect with my own suffering without judging myself. | 14. J'entre en contact avec ma propre souffrance sans me juger | 14. I experience my own suffering without self-judgment. | 14. I engage with my own suffering without judging myself. | |
| 15. When I'm upset, I try to do what's best for myself. | 15. Quand je suis bouleversé(e)/contrarié(e), j'essaie de faire ce qu'il y a de mieux pour moi | 15. When I am overwhelmed or upset, I try to do what is best for me. | 15. When I am distressed/upset, I try to do what's best for myself. | |
| 16. I recognise signs of suffering in myself. | 16. J'identifie les signes de souffrance en moi | 16. I identify signs of suffering in myself. | 16. I recognize signs of suffering in myself. | |
| 17. I know that we can all feel distressed when things don't go well in our lives. | 17. Je sais que nous pouvons tous nous sentir en détresse lorsque les choses ne vont pas bien dans notre vie | 17. I know that we can all feel distraught when things aren't going well in our lives. | 17. I know that we can all feel in distress when things aren't going well in our life. | |
| 18. Even when I'm disappointed with myself, I can feel warmly towards myself when I'm in distress. | 18. Même quand je suis déçu(e) de moi, je peux être bienveillant(e) envers moi-même lorsque je ressens de la détresse | 18. Even when I am disappointed in myself, I can treat myself with care when I am experiencing distress. | 18. Even if I am disappointed in myself, I can be caring towards myself when I am in distress. | Même quand je suis déçu(e) de moi, je peux me sentir bienveillant(e) envers moi-même lorsque je ressens de la détresse |
| 19. When I'm upset, I can let the emotions be there without feeling | 19. Lorsque je suis bouleversé(e)/contrarié(e), je laisse mes émotions exister sans | 19. When I am overwhelmed or upset, I let my emotions exist | 19. When I am distressed/upset, I let my feelings be without | |

**Commented [A6]:** The term 'j'essaie de me centrer' was changed to 'j'entre en contact' to more closely reflect the sense of 'connecting with/tuning in to' suffering in the original.

**Commented [A7]:** The term 'être' was replaced by 'me sentir' to ensure that the feeling aspect of the original version was retained (the distinction between feeling and acting as different subscales).

| | | | | |
|---|---|---|---|---|
| overwhelmed. | me sentir submergé(e) | without feeling like I am drowning in them. | feeling overwhelmed. | |
| 20. When I'm upset, I do my best to take care of myself. | 20. Quand je suis bouleversé(e)/contrarié(e), je fais de mon mieux pour prendre soin de moi | 20. When I am overwhelmed or upset, I try my best to take good care of myself (or engage in self-care). | 20. When I am distressed/upset, I do my best to take care of myself. | |
| **Scoring Information**<br><br>Users may compute both total subscale scores and a total SOCS-S score. To compute total subscale scores, sum item scores within each subscale (see below for items associated with each subscale). To compute a total SOCS-S score, sum all five total subscale scores or sum all 20 item scores.<br><br>Recognising suffering items: 1, 6, 11, 16.<br>Understanding the universality of suffering items: 2, 7, 12, 17.<br>Feeling for the person suffering items: 3, 8, 13, 18.<br>Tolerating uncomfortable feelings items: 4, 9, 14, 19.<br>Acting or being motivated to act to alleviate suffering items: 5, 10, 15, 20. | **Guide de notation**<br><br>Les utilisateurs peuvent calculer à la fois les scores totaux des sous-échelles et le score total SOCS-S. Pour calculer les scores totaux des sous-échelles, additionnez les scores de chaque item, et ce pour chaque sous-échelle (voir ci-dessous les items associés à chaque sous-échelle). Pour calculer le score total SOCS-S, additionnez le score total des cinq sous-échelles ou additionnez le score des 20 items.<br><br>Items liés à la reconnaissance de la souffrance : 1, 6, 11, 16.<br><br>Items liés à la compréhension de l'universalité de la souffrance : 2, 7, 12, 17.<br><br>Items liés à la sensibilité envers la personne qui souffre : 3, 8, 13, 18.<br><br>Items liés à la tolérance face aux sentiments inconfortables : 4, 9, 14, 19.<br><br>Items liés à l'action ou la motivation à agir pour soulager la souffrance : 5, 10, 15, 20. | Items assessing the ability to recognize suffering: 1, 6, 11, 16.<br><br>Items assessing one's understanding of the universality of suffering: 2, 7, 12, 17<br><br>Items assessing sensitivity toward the person who is suffering: 3, 8, 13, 18.<br><br>Items assessing tolerance for uncomfortable feelings: 4, 9, 14, 19<br><br>Items assessing actions or motivation to act to attenuate suffering: 5, 10, 15, 20. | Items related to recognizing suffering : 1, 6, 11, 16.<br><br>Items related to understanding the universality of suffering : 2, 7, 12, 17.<br><br>Items related to feeling for the person suffering : 3, 8, 13, 18.<br><br>Items related to tolerance of uncomfortable feelings : 4, 9, 14, 19.<br><br>Items related to acting or motivation to act to alleviate suffering : 5, 10, 15, 20. | |
