# Supplementary material for: Validation of the compassionate engagement and action scales, compassion scale, and Sussex-Oxford compassion scales in a French-Canadian sample
Source: PLoS One. 2024 Jun 24;19(6):e0305776. doi: 10.1371/journal.pone.0305776 (PMC11195958; doi:10.1371/journal.pone.0305776)
Supplement: S3 Appendix — French translations of the Compassionate Engagement and Action Scales, Compassion Scale, and Sussex-Oxford Compassion Scales for Self and Others. (ZIP) [file pone.0305776.s003.zip › S2 Translation Reports/CEAS.Translation Report.pdf]

The Compassionate Engagement and Action Scales

Les échelles de l'engagement et de l'action dans la compassion

Self-Compassion

L'auto-compassion

**English Original**	**French Translation**<br><br>*Independently translated (English to French) by two native French speakers, and synthesized during a consensus meeting.*	**Back Translation 1**	**Back Translation 2**	**French Translation after Back Translation**<br><br>*Where applicable, French items were revised following a committee meeting consisting of translators, back-translators, PI, and co-investigators.*
When things go wrong for us and we become distressed by setbacks, failures, disappointments or losses, we may cope with these in different ways. We are interested in the degree to which people can **be compassionate with themselves**. We define compassion as "a sensitivity to suffering in self and others with a commitment to try to alleviate and prevent it." This means there are two aspects to compassion. The *first* is the ability to be motivated to engage with things/feelings that are difficult as opposed to trying to avoid or suppress them. The *second* aspect of compassion is the ability to focus on what is helpful to us. Just like a	Lorsque les choses vont mal pour nous et que nous ressentons de la détresse vis-à-vis des difficultés, des échecs, des déceptions ou des pertes que nous vivons, nous pouvons faire face à cette détresse de différentes manières. Nous nous intéressons au degré de compassion dont les gens peuvent **faire preuve envers eux-mêmes**. Nous définissons la compassion comme « une sensibilité à la souffrance en soi et chez les autres accompagnée d'un dévouement à essayer de l'atténuer et de la prévenir. » Cela signifie que la compassion présente deux composantes. La *première* est la motivation à se mobiliser envers les choses et les émotions qui sont	When life is not going well and we feel distressed by the difficulties, failures, disappointments or losses we're facing, we can handle our distress in different ways. We're interested in the degree of **compassion** individuals are **capable of showing themselves**. We define compassion as follows: "a sensitivity to suffering in ourselves and in others along with a sense of dedication to attenuating and preventing such suffering." This definition comprises two important components. Firstly, the motivation to move toward difficult situations and emotions, rather than avoiding or suppressing them. Secondly, the ability to focus on what is effective	When things are going badly for us and we're feeling distress when confronted by the difficulties, failures, disappointments, or losses we experience, we can tackle this suffering in different ways. We are interested in the degree to which people can **demonstrate compassion towards themselves.** We define compassion as being "a sensitivity to the suffering in oneself and in others along with a commitment to mitigation and prevention." This means that compassion is made of two parts. The *first* is the motivation to take action on things and emotions that are difficult, instead of avoiding or suppressing them. The *second* part	

doctor with his/her patient. The first is to be motivated and able to pay attention to the pain and (learn how to) make sense of it. The second is to be able to take the action that will be helpful. Below is a series of questions that ask you about these two aspects of compassion. Therefore read each statement carefully and think about how it applies to you if you become distressed. Please rate the items using the following rating scale:<br>Never-Always (1-10)	difficiles, au lieu de les éviter ou de les réprimer. La *deuxième* composante de la compassion est la capacité à se concentrer sur ce qui nous est utile. Tout comme un médecin avec son patient. La première consiste à être capable et motivé(e) à porter attention à la douleur et à la comprendre (ou à apprendre à la comprendre). La deuxième est d'être capable de prendre des mesures qui seront utiles. Vous trouverez ci-dessous une série de questions portant sur ces deux composantes de la compassion. Veuillez donc lire attentivement chaque énoncé en pensant à la manière dont cela s'applique à vous lorsque vous vivez de la détresse. Veuillez évaluer les items au moyen de l'échelle suivante :<br>Jamais-Toujours (1-10)	or helpful. Just like a doctor with his or her patient: the first component consists of being able and willing to pay attention to and understand (or learn to understand) pain. The second component is being able to engage in measures that are helpful and/or effective. You will find below a series of questions regarding these two components of compassion. Please read each item attentively while considering the degree to which they apply to you in situations of distress. Please evaluate the items using the following scale:<br>Never-Always (1-10)	of compassion is the ability to concentrate on what is useful to us. Just like a doctor does with their patient. The first part is being able and motivated of paying attention to pain and to understand it (or to learn to understand it). The second is being able to take useful action. In the following you will find a series of questions that relate to these two parts of compassion. With that in mind, please read each statement carefully, reflecting on how they relate to you when you experience suffering. Please evaluate the items using the following scale:<br>Never – Always (1-10)	
**Section 1 – These are questions that ask you about how motivated you are, and able to engage with distress when you experience it. So:**<br><br>**When I'm distressed or upset by things…**	**Section 1 – Voici des questions concernant votre niveau de motivation et votre capacité à faire face à la détresse que vous ressentez. Donc :**<br><br>**Lorsque je suis angoissé(e), bouleversé(e), ou contrarié(e) par quelque chose …**	**Section 1 – Here are some questions regarding your level of ability and willingness to face the distress you feel.**<br><br>***When I am distraught, overwhelmed or upset by something…***	**Section 1 – Here are some questions regarding your level of motivation and ability to handle the distress that you experience:**<br><br>**When I am anxious, overwhelmed, or upset by something…**	**Section 1 – Voici des questions concernant votre niveau de motivation et votre capacité à faire face à la détresse que vous ressentez. Donc :**<br><br>**Lorsque je suis en détresse, bouleversé(e), ou contrarié(e) par quelque chose …**
1. I am *motivated* to engage and work with my distress when it arises.	1. Je suis *motivé(e)* à m'engager et à gérer ma détresse lorsqu'elle survient.	1. I am *motivated* to engage with and manage my distress as it surfaces.	1. I am motivated to take action and deal with my distress when it occurs.	
2. I *notice*, and am *sensitive* to my distressed feelings when they	2. Je *remarque* et je suis *sensible* à mes sentiments de détresse	2. I *notice* and am *sensitive* to my feelings of distress as they arise.	2. I notice and am sensitive to my feelings of distress when they	

Commented [A1]: In the online validation study of the French translations, this text was included only in the background/directions for the CEAS-SC and was omitted in the CEAS scales that appeared subsequently (CEAS-TO and CEAS-FROM). This decision was based on feedback during the translation process, during which test-takers shared that they found it repetitive to read the full directions for all three scales when the scales appeared consecutively.

Commented [A2]: The word 'angoissé(e)' was replaced with 'en détresse' to more closely reflect the term 'distress'.

arise in me.	lorsqu'ils surviennent.		appear.	
3. I avoid thinking about my distress and try to distract myself and put it out of my mind. (r)	3. J'évite de penser à ma détresse et j'essaie de me distraire et de la chasser de mon esprit. (r)	3. I avoid thinking about my distress and try to distract myself and get rid of it. (r)	3. I avoid acknowledging my distress and try to distract myself and get it out of my mind. (r)	
4. I am *emotionally moved* by my distressed feelings or situations.	4. Je suis *ému(e)* ou émotionnellement touché(e) par les situations ou sentiments de détresse que je vis.	4. I am *moved* (or rendered speechless) or seized with emotion in response to the distressing situation or feelings that I am experiencing.	4. I am affected or emotionally touched by the situations or feelings of distress that I experience.	
5. I *tolerate* the various feelings that are part of my distress.	5. Je *tolère* les divers sentiments qui font partie de ma détresse.	5. I *tolerate* the various emotions that are part of my overall distress.	5. I *tolerate* the various feelings that are part of my distress.	
6. I *reflect on* and *make sense* of my feelings of distress.	6. Je *réfléchis* sur les sentiments de détresse que je ressens et j'essaie de les *comprendre*.	6. I *reflect* on the feelings of distress I am experiencing and try to *understand* them.	6. I *reflect* on the feelings of distress that I feel and try to understand them.	
7. I do not tolerate being distressed. (r)	7. Je ne tolère pas de ressentir de la détresse. (r)	7. I do not tolerate my feelings of distress. (r)	7. I do not tolerate feeling distress. (r)	
8. I am *accepting, non-critical and non-judgemental* of my feelings of distress.	8. J'*accepte*, je suis *non-critique* et je *ne porte pas de jugement* sur mes sentiments de détresse.	8. I am *accepting, non-critical* and *non-judgmental* of my feelings of distress.	8. I *accept*, I am *non-critical* and I *do not place judgement* on my feelings of distress.	
**Section 2 – These questions relate to how you actively cope in compassionate ways with emotions, thoughts and situations that distress you.**<br>**So:**<br><br>**When I'm distressed or upset by things…**	**Section 2 – Ces questions concernent les façons dont vous faites activement face, avec compassion, aux émotions, aux pensées et aux situations qui provoquent de la détresse en vous.**<br>**Donc :**<br><br>**Lorsque je suis angoissé(e), bouleversé(e), ou contrarié(e) par quelque chose …**	**Section 2 – These questions concern the ways in which you actively face distressing emotions, thoughts, and situations with compassion.**<br><br>***When I am distraught, overwhelmed or upset by something…***	**Section 2 – This questions deal with the ways in which you, with compassion, actively deal with emotions, thoughts and situations that cause distress in you:**<br><br>**When I am anxious, overwhelmed, or upset by something…**	**Section 2 – Ces questions concernent les façons dont vous faites activement face, avec compassion, aux émotions, aux pensées et aux situations qui provoquent de la détresse en vous.**<br>**Donc :**<br><br>**Lorsque je suis en détresse, bouleversé(e), ou contrarié(e) par quelque chose …**
1. I direct my *attention* to what is likely to be helpful to me.	1. Je dirige mon *attention* vers ce qui pourrait m'aider.	1. I direct my *attention* toward what might help me.	**1.** I focus my attention on what can help me.	
2. I *think* about and come up with helpful ways to cope with my	2. Je *réfléchis* et je trouve des façons utiles pour faire face à	2. I *think* about useful or helpful ways to face my distress.	2. I reflect and find helpful ways of dealing with my distress.	

Commented [A3]: The word 'angoissé(e)' was replaced with 'en détresse' to more closely reflect the term 'distress'.

distress.	ma détresse.			
3. I don't know how to help myself. (r)	3. Je ne sais pas comment m'aider moi-même. (r)	3. I do not know how to help myself. (r)	3. I don't know how to help myself. (r)	
4. I take the *actions* and do the things that will be helpful to me.	4. Je prends les *mesures* et fais les choses qui pourront m'aider.	4. I take *action* and do things that might help me.	4. I take *action* and do things that will help me.	
5. I create inner feelings of *support, helpfulness and encouragement*.	5. Je génère en moi des sentiments de *soutien*, d'*aide* et d'*encouragement*.	5. I generate feelings of *support, help,* and *encouragement* in myself.	5. I create feelings of *support, help* and *encouragement* in myself.	
**NOTE FOR USERS: REVERSE ITEMS (r) ARE NOT INCLUDED IN THE SCORING**	**À NOTER : LES ITEMS INVERSÉS (r) NE SONT PAS INCLUS DANS LA NOTATION**	**NOTE: Reverse items (r) are not included in the scoring.**	**PLEASE NOTE: THE REVERSE ITEMS (r) ARE NOT INCLUDED IN THE SCORE.**	

Compassion To Others

Compassion envers les autres

English Original	French Translation	Back Translation 1	Back Translation 2	French Translation after Back Translation
When things go wrong for other people and they become distressed by setbacks, failures, disappointments or losses, we may cope with their distress in different ways. We are interested in the degree to which people can be **compassionate to others**. We define compassion as "a sensitivity to suffering in self and others with a commitment to try to alleviate and prevent it." This means there are two aspects to compassion. The *first* is the ability to be motivated to engage with things/feelings that are difficult as opposed to trying to avoid or suppress them. The *second* aspect of compassion is the ability to focus on what is helpful. Just like a doctor with his/her patient. The first is to be motivated and able to pay attention to the pain and (learn how to) make sense of it. The second is to be able to take the action that will be helpful. Below is a series of questions that ask you about these two aspects of compassion. Therefore read each statement carefully and think about how it applies to you when **people in your life** become distressed. Please rate	Lorsque les choses vont mal pour les autres et qu'ils ou elles ressentent de la détresse vis-à-vis des difficultés, des échecs, des déceptions ou des pertes qu'ils ou elles vivent, nous pouvons faire face à leur détresse de différentes manières. Nous nous intéressons au degré de **compassion** dont les gens peuvent faire preuve **envers les autres**. Nous définissons la compassion comme « une sensibilité à la souffrance en soi et chez les autres accompagnée d'un dévouement à essayer de l'atténuer et de la prévenir. » Cela signifie que la compassion présente deux composantes. La *première* est la motivation à se mobiliser envers les choses et les émotions qui sont difficiles, au lieu de les éviter ou de les réprimer. La *deuxième* composante de la compassion est la capacité à se concentrer sur ce qui nous est utile. Tout comme un médecin avec son patient. La première est d'être motivé et capable de prêter attention à la douleur et (apprendre à) la comprendre. La deuxième est d'être capable de prendre des mesures qui seront utiles. Vous trouverez ci-dessous	When life is not going well for others and they are feeling by the difficulties, failures, disappointments or losses they are facing, we can handle their distress in different ways. We're interested in the degree of **compassion** individuals are capable of showing **others**. We define compassion as follows: "a sensitivity to suffering in ourselves and in others along with a sense of dedication to attenuating and preventing such suffering." This definition comprises two important components. Firstly, the motivation to move toward difficult situations and emotions, rather than avoiding or suppressing them. Secondly, the ability to focus on what is effective or helpful. Just like a doctor with his or her patient: the first component consists of being able and willing to pay attention to and understand (or learn to understand) pain. The second component is being able to engage in measures that are helpful and/or effective. You will find below a series of questions regarding these two components of compassion. Please read each item attentively while considering the	When things are going poorly for others or they are feeling distress when confronted by difficulties, failures, disappointments or losses that they experience, we can tackle this suffering in different ways. We are interested in the degree to which people can **demonstrate compassion towards others.** We define compassion as being "a sensitivity to the suffering in oneself and in others along with a commitment to mitigation and prevention." This means that compassion is made of two parts. The *first* is the motivation to take action towards the things and emotions that are difficult, instead of avoiding or suppressing them. The *second* part of compassion is the ability to concentrate on what is useful to us. Just like a doctor does with their patient. The first part is being able and motivated of paying attention to pain and to understand it (or to learn to understand it). The second is being able to take useful action. In the following you will find a series of questions that relate to these two parts of compassion. With that in mind, please read each	

Commented [A4]: In the online validation study of the French translations, this text was included only in the background/directions for the CEAS-SC and was omitted in the CEAS scales that appeared subsequently (CEAS-TO and CEAS-FROM). This decision was based on feedback during the translation process, during which test-takers shared that they found it repetitive to read the full directions for all three scales when the scales appeared consecutively.

the items using the following rating scale:<br>Never-Always (1-10)	une série de questions portant sur ces deux composantes de la compassion. Veuillez donc lire attentivement chaque énoncé en pensant à la manière dont cela s'applique à vous lorsque des **personnes dans votre vie vivent de la détresse**. Veuillez évaluer les items au moyen de l'échelle suivante :<br><br>Jamais-Toujours (1-10)	degree to which they apply to you **when people in your life are experiencing distress**. Please evaluate the items using the following scale:<br>Never-Always (1-10)	statement carefully, reflecting on how they relate to you when **people in your life experience distress**. Please evaluate the items using the following scale:<br>Never-Always (1-10)	
**Section 1 – These are questions that ask you about how motivated you are, and able to engage with other people's distress when they are experiencing it. So:**<br><br>**When others are distressed or upset by things…**	**Section 1 – Voici des questions concernent votre niveau de motivation et votre capacité à faire face à la détresse que d'autres personnes ressentent. Donc :**<br><br>**Lorsque les autres sont angoissé(e)s, bouleversé(e)s, ou contrarié(e)s par quelque chose …**	**Section 1 – Here are some questions regarding your level of ability and willingness to face the distress experienced by other people in your life.**<br><br>***When others are distraught, overwhelmed or upset by something…***	**Section 1 – Here are some questions regarding your level of motivation and ability to handle the distress that other people experience:**<br><br>**When others are anxious, overwhelmed, or upset by something…**	**Section 1 – Voici des questions concernent votre niveau de motivation et votre capacité à faire face à la détresse que d'autres personnes ressentent. Donc :**<br><br>**Lorsque je suis en détresse, bouleversé(e), ou contrarié(e) par quelque chose …**
1. I am *motivated* to engage and work with other peoples' distress when it arises.	Je suis *motivé(e)* à m'engager et à gérer la détresse des autres lorsqu'elle survient.	1. I am *motivated* to engage with and manage others' distress as it surfaces.	1. I am *motivated* to take action and deal with others' distress when it occurs.	
2. I *notice* and *am sensitive* to distress in others when it arises.	Je *remarque* et je suis *sensible* à la détresse des autres lorsqu'elle survient.	2. I *notice* and am *sensitive* to others' feelings of distress as they arise.	2. I *notice* and am sensitive to others' feelings of distress when they appear.	
3. I avoid thinking about other peoples' distress, try to distract myself and put it out of my mind.<br>(r)	J'évite de penser à la détresse des autres et j'essaie de me distraire et de la chasser de mon esprit.<br>(r)	3. I avoid thinking about other people's distress and try to distract myself and push it from my mind.<br>(r)	3. I avoid acknowledging others' distress and try to distract myself and get it out of my mind.<br>(r)	
4. I am *emotionally moved* by expressions of distress in others.	Je suis *ému(e)* ou émotionnellement touché(e) par les manifestations de détresse des autres.	4. I am *moved* (or rendered speechless) or seized with emotion in response to distressing situations or feelings	4. I am affected or emotionally touched by others' situations or feelings of distress.	

Commented [A5]: The word 'angoissé(e)' was replaced with 'en détresse' to more closely reflect the term 'distress'.

		experienced by others.		
5. I *tolerate* the various feelings that are part of other people's distress.	Je *tolère* les divers sentiments qui font partie de la détresse des autres.	5. I *tolerate* the various emotions that are part of others' overall distress.	5. I *tolerate* the various feelings that are part of others' distress.	
6. I *reflect on* and *make sense* of other people's distress.	Je *réfléchis* sur et j'*essaie de comprendre* la détresse des autres.	6. I *reflect* on the feelings of distress others are experiencing and try to *understand* them.	6. I *reflect* on others' feelings of distress and try to understand them.	
7. I do not tolerate other peoples' distress. (r)	Je ne tolère pas la détresse des autres. (r)	7. I do not tolerate feelings of distress in others. (r)	7. I do not tolerate others' distress. (r)	
8. I am *accepting, non-critical and non-judgmental* of others people's distress.	J'*accepte*, je suis *non-critique* et je ne *porte pas de jugement* sur la détresse des autres.	8. I am *accepting, non-critical* and *non-judgmental* of others' feelings of distress.	8. I *accept*, I am *non-critical* and I *do not place judgement* on others' distress.	
**Section 2 – These questions relate to how you actively respond in compassionate ways when other people are distressed. So: When others are distressed or upset by things…**	**Section 2 – Ces questions concernent les façons dont vous répondez activement, avec compassion, lorsque d'autres personnes vivent de la détresse. Donc :**<br><br>**Lorsque les autres sont angoissé(e)s, bouleversé(e)s, ou contrarié(e)s par quelque chose …**	**Section 2 – These questions concern the ways in which you actively respond with compassion to the distressing emotions, thoughts, and situations experienced by others.**<br><br>***When others are distraught, overwhelmed or upset by something…***	**Section 2 – This questions deal with the ways in which you actively respond with compassion when others experience distress:**<br><br>**When others are anxious, overwhelmed, or upset by something…**	**Section 2 – Ces questions concernent les façons dont vous répondez activement, avec compassion, lorsque d'autres personnes vivent de la détresse. Donc :**<br><br>**Lorsque je suis en détresse, bouleversé(e), ou contrarié(e) par quelque chose …**
1. I direct *attention* to what is likely to be helpful to others.	Je dirige mon *attention* vers ce qui pourrait aider les autres.	1. I direct my *attention* toward what might help them.	1. I focus my attention on what might help others.	
2. I *think about and come up* with helpful ways for them to cope with their distress.	Je *réfléchis et trouve* des façons utiles pour que les autres puissent faire face à leur détresse.	2. I *think* about useful or helpful ways for others to face their distress.	2. I *reflect and find* helpful ways for others to handle their distress.	
3. I don't know how to help other people when they are distressed. (r)	Je ne sais pas comment aider les autres lorsqu'ils ou elles ressentent de la détresse. (r)	3. I do not know how to help other people when they are feeling distress. (r)	3. I don't know how to help others when they experience distress. (r)	
4. I take the *actions* and *do the things* that will be helpful to others.	Je prends les *mesures* et *fais les choses* qui pourront aider les autres.	4. I take *action* and *do things* that might help them.	4. I take *action* and *do things* that will help others.	
5. I express feelings of *support,*	J'exprime des sentiments de *soutien,*	5. I express sentiments of *support,*	5. I express feelings of *support, help*	

Commented [A6]: The word 'angoissé(e)' was replaced with 'en détresse' to more closely reflect the term 'distress'.

helpfulness and encouragement to others.	d'*aide* et d'*encouragement* envers les autres.	*help,* and *encouragement* for others.	and *encouragement* towards others.	
**NOTE FOR USERS: REVERSE ITEMS (r) ARE NOT INCLUDED IN THE SCORING**	**À NOTER : LES ITEMS INVERSÉS (r) NE SONT PAS INCLUS DANS LA NOTATION**	**NOTE: Reverse items (r) are not included in the scoring.**	**PLEASE NOTE: THE REVERSE ITEMS (r) ARE NOT INCLUDED IN THE SCORE.**	

Compassion from Others

Compassion de la part des autres

**English Original**	**French Translation**	**Back Translation 1**	**Back Translation 2**	**French Translation after Back Translation**
When things go wrong for us and we become distressed by setbacks, failures, disappointments or losses, others may cope with our distress in different ways. We are interested in the degree to which you feel that important people in your life can be compassionate to your distress. We define compassion as "a sensitivity to suffering in self and others with a commitment to try to alleviate and prevent it." This means there are two aspects to compassion. The first is the ability to be motivated to engage with things/feelings that are difficult as opposed to trying to avoid or supress them. The second aspect of compassion is the ability to focus on what is helpful to us or others. Just like a doctor with his/her patient. The first is to be motivated and able to pay attention to the pain and (learn how to) make sense of it. The second is to be able to take the action that will be helpful. Below is a series of questions that ask you about these two aspects of compassion. Therefore read each statement carefully and think about how it applies to the important people in your life when you	Lorsque les choses vont mal pour nous et que nous ressentons de la détresse vis-à-vis des difficultés, des échecs, des déceptions ou des pertes que nous vivons, les autres peuvent faire face à notre détresse de différentes manières. Nous nous intéressons au degré de compassion dont les personnes importantes dans votre vie font preuve lorsque **vous êtes en détresse**. Nous définissons la compassion comme « une sensibilité à la souffrance en soi et chez les autres accompagnée d'un dévouement à essayer de l'atténuer et de la prévenir. » Cela signifie que la compassion présente deux composantes. La *première* est la motivation à se mobiliser envers les choses et les émotions qui sont difficiles, au lieu de les éviter ou de les réprimer. La *deuxième* composante de la compassion est la capacité à se concentrer sur ce qui nous est utile. Tout comme un médecin avec son patient. La première est d'être motivé et capable de prêter attention à la douleur et (apprendre à) la comprendre. La deuxième est d'être capable de prendre des mesures qui seront	When life is not going well and we feel distressed by the difficulties, failures, disappointments or losses we are facing, others can react to our distress in different ways. We're interested in the degree of **compassion** that **important people in your life** show you when **you are distressed**. We define compassion as follows: "a sensitivity to suffering in ourselves and in others along with a sense of dedication to attenuating and preventing such suffering." This definition comprises two important components. Firstly, the motivation to move toward difficult situations and emotions, rather than avoiding or suppressing them. Secondly, the ability to focus on what is effective or helpful. Just like a doctor with his or her patient: the first component consists of being able and willing to pay attention to and understand (or learn to understand) pain. The second component is being able to engage in measures that are helpful and/or effective. You will find below a series of questions regarding these two components of compassion. Please read each item attentively while considering the	When things are going poorly for us or when we are feeling distress when confronted by difficulties, failures, disappointments or losses that we experience, others can deal with our distress in different ways. We are interested in the degree to which important people in our life can demonstrate compassion **when you are in distress.** We define compassion as being "a sensitivity to the suffering in oneself and in others along with a commitment to mitigation and prevention." This means that compassion is made of two parts. The *first* is the motivation to take action towards the things and emotions that are difficult, instead of avoiding or suppressing them. The *second* part of compassion is the ability to concentrate on what is useful to us. Just like a doctor does with their patient. The first part is being able and motivated of paying attention to pain and to understand it (or to learn to understand it). The second is being able to take useful action. In the following you will find a series of questions that relate to these two parts of compassion. With that in mind, please read each	

become distressed. Please rate the items using the following rating scale:<br>Never-Always (1-10)	utiles. Vous trouverez ci-dessous une série de questions portant sur ces deux composantes de la compassion. Veuillez donc lire attentivement chaque énoncé en pensant à la manière dont cela s'applique aux personnes importantes de votre vie lorsque vous vivez de la détresse. Veuillez évaluer les items au moyen de l'échelle suivante :<br>Jamais-Toujours (1-10)	degree to which they apply to **important people in your life** when **you are experiencing distress**. Please evaluate the items using the following scale:<br>Never-Always (1-10)	statement carefully, reflecting on how they relate to important people in your life when you experience distress. Please evaluate the items using the following scale:<br>Never-Always (1-10)	
**Section 1 – These are questions that ask you about how motivated you think others are, and how much they engage with your distress when you experience it. So:**<br><br>**When I'm distressed or upset by things…**	**Section 1 – Voici des questions mesurant à quel point vous pensez que les autres sont motivés et à quel point qu'ils ou elles s'impliquent dans votre détresse lorsque vous en faites l'expérience. Donc :**<br><br>**Lorsque je suis angoissé(e), bouleversé(e), ou contrarié(e) par quelque chose …**	**Section 1 – Here are some questions regarding the degree to which important people in your life are able and willing to face your distress.**<br><br>***When I am distraught, overwhelmed or upset by something…***	**Section 1 – Here are some questions measuring to which degree you believe that others are motivated and engage with your suffering when you experience it.**<br><br>**When I am anxious, overwhelmed, or upset by something…**	**Section 1 – Voici des questions mesurant à quel point vous pensez que les autres sont motivés et à quel point qu'ils ou elles s'impliquent dans votre détresse lorsque vous en faites l'expérience. Donc :**<br><br>**Lorsque je suis en détresse, bouleversé(e), ou contrarié(e) par quelque chose …**
1. Other people are actively motivated to engage and work with my distress when it arises.	1. Les autres personnes sont activement *motivées* à s'engager et à gérer ma détresse lorsqu'elle survient.	1. The people in my life are actively *motivated* to engage with and manage my distress as it surfaces.	1. Others are actively motivated to take action and deal with my distress when it occurs.	
2. Others notice and are sensitive to my distressed feelings when they arise in me.	2. Les autres *remarquent* et sont *sensibles* à mes sentiments de détresse lorsqu'ils surviennent.	2. The people in my life *notice* and are *sensitive* to my feelings of distress as they arise.	2. Others notice and are sensitive to my feelings of distress when they appear.	
3. Others avoid thinking about my distress, try to distract themselves and put it out of their mind.<br>(r)	3. Les autres évitent de penser à ma détresse, essaient de se distraire et de la chasser de leur esprit.<br>(r)	3. The people in my life avoid thinking about my distress and try to distract themselves from it and push it out of their minds.<br>(r)	3. Others avoid acknowledging my distress and try to distract themselves and get it out of their minds.<br>(r)	
4. Others are *emotionally moved* by	4. Les autres sont *ému(e)s* ou	4. The people in my life are *moved*	4. Others are *affected* or *emotionally*	

Commented [A7]: In the online validation study of the French translations, this text was included only in the background/directions for the CEAS-SC and was omitted in the CEAS scales that appeared subsequently (CEAS-TO and CEAS-FROM). This decision was based on feedback during the translation process, during which test-takers shared that they found it repetitive to read the full directions for all three scales when the scales appeared consecutively.

Commented [A8]: The word 'angoissé(e)' was replaced with 'en détresse' to more closely reflect the term 'distress'.

my distressed feelings.	*émotionnellement touché(e)s* par mes sentiments de détresse.	(or rendered speechless) or seized with emotion in response to the distressing situations or feelings that I am experiencing.	*touched* by my feelings of distress.	
5. Others *tolerate* my various feelings that are part of my distress.	5. Les autres *tolèrent* les divers sentiments qui font partie de ma détresse.	5. The people in my life *tolerate* the various emotions that are part of my overall distress.	5. Others *tolerate* the various feelings that are part of my distress.	
6. Others reflect on and make sense of my feelings of distress.	6. Les autres *réfléchissent* sur mes sentiments de détresse et *essaient de les comprendre.*	6. The people in my life *reflect* on my feelings of distress and try to *understand* them.	6. Others reflect on the feelings of distress that I feel and try to understand them.	
7. Others do not tolerate my distress. (r)	7. Les autres ne tolèrent pas ma détresse. (r)	7. The people in my life do not tolerate my feelings of distress. (r)	7. Others do not tolerate my suffering. (r)	
8. Others are accepting, non-critical and non-judgemental of my feelings of distress.	8. Les autres *acceptent*, *ne critiquent pas* et *ne jugent pas* mes sentiments de détresse.	8. The people in my life are *accepting, non-critical* and *non-judgmental* of my feelings of distress.	8. Others *accept*, are *non-critical* and *do not judge* my feelings of distress.	
**Section 2 – These questions relate to how others actively cope in compassionate ways with emotions and situations that distress you. So:**<br>**When I'm distressed or upset by things…**	**Section 2 – Ces questions concernent les façons dont les autres font activement face, avec compassion, aux émotions et aux situations qui provoquent de la détresse en vous. Donc :**<br><br>**Lorsque je suis angoissé(e), bouleversé(e), ou contrarié(e) par quelque chose…**	**Section 2 – These questions concern the ways in which the people in your life actively respond with compassion to the distressing emotions, thoughts, and situations that you experience.**<br><br>***When I am distraught, overwhelmed or upset by something…***	**Section 2 – This questions deal with the ways in which others, with compassion, actively deal with emotions, thoughts and situations that cause distress in you:**<br><br>**When I am anxious, overwhelmed, or upset by something…**	**Section 2 – Ces questions concernent les façons dont les autres font activement face, avec compassion, aux émotions et aux situations qui provoquent de la détresse en vous. Donc :**<br><br>**Lorsque je suis en détresse, bouleversé(e), ou contrarié(e) par quelque chose …**
1. Others direct their attention to what is likely to be helpful to me.	1. Les autres dirigent leur *attention* vers ce qui est susceptible de m'aider.	1. The people in my life direct their *attention* toward what might help me.	1. Others focus their attention on what can help me.	
2. Others think about and come up with helpful ways for me to cope with my distress.	2. Les autres *pensent à* et trouvent des façons utiles afin que je puisse faire face à ma détresse.	2. The people in my life *think* about useful or helpful ways for me to face my distress.	2. Others reflect and find helpful ways of dealing with my distress.	
3. Others don't know how to help me when I am distressed. (r)	3. Les autres ne savent pas comment m'aider lorsque je ressens de la détresse.	3. The people in my life do not know how to help me when I am feeling distraught.	3. Others don't know how to help me when I am experiencing distress.	

Commented [A9]: The word 'angoissé(e)' was replaced with 'en détresse' to more closely reflect the term 'distress'.

	(r)	(r)	(r)	
4. Others take the actions and do the things that will be helpful to me.	4. Les autres prennent des *mesures* et *font des choses* qui m'aideront.	4. The people in my life take *action* and *do things* that might help me.	4. Others take *action* and do things that will help me.	
5. Others treat me with feelings of support, helpfulness and encouragement	5. Les autres me traitent avec des sentiments de *soutien*, d'*aide* et d'*encouragement*.	5. The people in my life express to me sentiments of *support, help,* and *encouragement*.	*5.* Others treat me with feelings of *support, help* and *encouragement*.	
**NOTE FOR USERS: REVERSE ITEMS (r) ARE NOT INCLUDED IN THE SCORING**	**À NOTER : LES ITEMS INVERSÉS (r) NE SONT PAS INCLUS DANS LA NOTATION**	**NOTE: Reverse items (r) are not included in the scoring.**	**PLEASE NOTE: THE REVERSE ITEMS (r) ARE NOT INCLUDED IN THE SCORE.**	
**SCORING**<br><br>The three scales – Compassion for others, compassion from others, compassion for self are scored separately.<br>For each scale two subscales can be calculated: Engagement (items 1, 2, 4, 5, 6, 8) and Actions (1, 2, 4, 5).<br>For the *Compassion for self* scale, two dimensions may be analysed in the Engagement subscale (sum of items 2 and 4, and sum of items 1, 5, 6, and 8).<br>A total score can be calculated (sum of items of the Engagement and Actions subscales) for each scale – *Compassion for others, compassion from others, compassion for self*.<br>Please note that reverse items (r) are not included in the scoring.<br><br>**DESCRIPTION**<br><br>*The Compassionate Engagement and Action Scales*	**NOTATION**<br><br>Les trois échelles – Compassion pour les autres, compassion de la part des autres, et compassion pour soi sont notées séparément.<br>Pour chaque échelle, deux sous-échelles peuvent être calculées : l'échelle portant sur l'engagement (items 1, 2, 4, 5, 6, 8) et l'échelle portant sur les actions (1, 2, 4, 5).<br>Pour l'échelle *Compassion pour soi*, deux dimensions peuvent être analysées dans la sous-échelle Engagement (la somme des items 2 et 4, et la somme des items 1, 5, 6, et 8).<br>Un score total peut être calculé (la somme des items des sous-échelles Engagement et Actions) pour chaque échelle – *Compassion pour les autres, compassion de la part des autres,* et *compassion pour soi*.<br>Veuillez noter que les items inversés (r) ne sont pas inclus dans la notation.	**SCORING**<br><br>The three scales – Self-Compassion, Compassion for Others, and Compassion from Others – are scored separately.<br><br>For each scale, two subscales can be calculated: the Engagement subscale (items 1, 2, 4, 5, 6, 8) and the Action subscale (1, 2, 4, 5). For the Self-Compassion scale, two dimensions can be analysed in the Engagement subscale (the sum of items 2 and 4, and the sum of items 1, 5, 6, and 8).<br><br>A total score can be calculated (the sum of the Engagement and Action subscales) for each scale (Self-Compassion, Compassion for Others, Compassion from Others).<br><br>Please note that reverse items (r) are not included in the scoring.<br><br>**DESCRIPTION**	**SCORING**<br><br>The three scales – Compassion towards others, compassion from others, and compassion for oneself are scored independently.<br>For each scale, two subscales can be calculated: the scale dealing with engagement (items 1, 2, 4, 5, 6, 8) and the scale dealing with actions (1, 2, 4, 5).<br>For the *Self-compassion* scale, two aspects can be analyzed under the Engagement subscale (the sum of items 2 and 4, and the sum of items 1, 5, 6, and 8).<br>A total score can be calculated (the sum of the items of Engagement and Action subscales) for each scale – Compassion towards others, compassion from others, and compassion for oneself.<br>Please note that the revers items (r) are not included in the score.<br><br>**DESCRIPTION**	

The Compassionate Engagement and Action Scales are three scales which measure self-compassion ("I am motivated to engage and work with my distress when it arises"), the ability to be compassionate to distressed others ("I am motivated to engage and work with other peoples' distress when it arises") and the ability to receive compassion from key persons in the respondent's life ("Other people are actively motivated to engage and work with my distress when it arises"). In the first section of each scale, six items are formulated to reflect the six compassion attributes in the CFT model: sensitivity to suffering, sympathy, non-judgemental, empathy, distress tolerance and care for wellbeing. These sections also include two reversed filler items. The second section of the scale has four more items which reflect specific compassionate actions to deal with distress and an extra reversed filler item. Participants are asked to rate each statement according to how frequently it occurs on a scale of 1 to 10 (1 = Never; 10= Always).	**DESCRIPTION**<br><br>*Les échelles de l'engagement et de l'action dans la compassion*<br><br>Les échelles de l'engagement et de l'action dans la compassion sont trois échelles qui mesurent l'auto-compassion (« Je suis *motivé(e)* à m'engager et à gérer ma détresse lorsqu'elle survient »), la capacité à ressentir de la compassion envers les autres en détresse (« Je suis *motivé(e)* à m'engager et à gérer la détresse des autres lorsqu'elle survient ») et la capacité à recevoir de la compassion de la part de personnes importantes dans la vie du répondant (« Les personnes autour de moi sont motivées à s'engager et à confronter ma détresse lorsqu'elle survient »). Dans la première section de chaque échelle, six items sont formulés pour refléter les six attributs retrouvés dans le modèle CFT : la sensibilité à la souffrance, la sympathie, le non-jugement, l'empathie, la tolérance à la détresse, et le soin du bien-être. Ces sections comprennent également deux éléments de remplissage inversés. La deuxième section de l'échelle comprend quatre autres items qui reflètent des actions de compassion spécifiques pour faire face à la détresse et un item de remplissage	*Compassionate Engagement and Action Scales*<br><br>The Compassionate Engagement and Action scales are three scales measuring self-compassion ("I am *motivated* to engage with and manage my distress as it surfaces"), compassion for others ("I am *motivated* to engage with and manage others' distress as it surfaces"), and the ability to accept compassion from other important people in one's life ("The people in my life are actively *motivated* to engage with and manage my distress as it surfaces"). In the first section of each scale, 6 items are formulated to reflect 6 attributes of the CFT model: sensitivity to suffering, sympathy, non-judgment, empathy, tolerance of distress, and self-care. These sections also comprise two reverse items. The second section of the scale comprises four other items that reflect concrete, specific compassionate actions to face distress, as well as one reverse item. Participants are asked to rank each statement in terms of frequency on a scale ranging from 1 to 10 (1 = Never; 10 = Always).	*Scales of engagement and action in compassion*<br><br>The scales of engagement and action in compassion are three scales which measure self-compassion ("I am motivated to take action and deal with my suffering when it occurs"), the ability to feel compassion towards others who are suffering ("I am motivated to take action and deal with the suffering of others when it occurs") and the ability to receive compassion from important people in the respondent's life ("The people around me are motivated to take action and deal with my suffering when it occurs"). In the first section of each scale, six items are articulated to reflect the six attributes found in the CFT model: sensitivity to suffering, sympathy, non-judgement, empathy, tolerating suffering, and caring for wellbeing. These sections also include two parts of inverted filling. The scale's second section includes four other items that reflect the specific acts of compassion to deal with suffering and one item of inverted filling. Participants are invited to evaluate each statement according to the rate at which it occurs on a scale from 1 to 10 (1 = Never ; 10 = Always).	

	inversé. Les participants sont invités à évaluer chaque énoncé en fonction de la fréquence à laquelle cela se produit sur une échelle de 1 à 10 (1 = Jamais ; 10 = Toujours).			
