# Supplementary material for: Validation of the compassionate engagement and action scales, compassion scale, and Sussex-Oxford compassion scales in a French-Canadian sample
Source: PLoS One. 2024 Jun 24;19(6):e0305776. doi: 10.1371/journal.pone.0305776 (PMC11195958; doi:10.1371/journal.pone.0305776)
Supplement: S3 Appendix — French translations of the Compassionate Engagement and Action Scales, Compassion Scale, and Sussex-Oxford Compassion Scales for Self and Others. (ZIP) [file pone.0305776.s003.zip › S2 Translation Reports/CS.Translation Report.pdf]

Compassion Scale

L'échelle de compassion (EC)

**English Original**	**French Translation**<br><br>*Independently translated (English to French) by two native French speakers, and synthesized during a consensus meeting.*	**Back-translation 1**	**Back-translation 2**	**French Translation after Back Translation**<br><br>*Where applicable, French items were revised following a committee meeting consisting of translators, back-translators, PI, and co-investigators.*
Instructions: Please read each statement carefully before answering. Indicate how often you feel or behave in the stated manner on a scale from 1 'Almost Never' to 5 'Almost Always.' Please answer according to what really reflects your experience rather than what you think your experience should be.	Instructions : Veuillez lire attentivement chaque énoncé avant de répondre. Indiquez la fréquence à laquelle vous vous sentez ou vous comportez de la manière indiquée sur une échelle de 1 « presque jamais » à 5 « presque toujours ». Veuillez répondre selon votre expérience réelle et non selon ce que vous croyez que votre expérience devrait être.	Instructions: Please read each statement attentively before answering. Indicate the frequency of which you feel or behave in the manner indicated by each statement by using a scale ranging from 1 "almost never" to 5 "almost always." Please answer based on your actual lived experience, and not based on how you *think* you *should* feel or act.	Instructions : Please read each statement carefully before answering. Indicate to which degree you feel or act in the way described on a scale from 1 "almost never" to 5 "almost always." Please respond according to your real experience rather than how you believe your experience should be.	
1. I pay careful attention when other people talk to me about their troubles.	1. J'accorde une attention toute particulière aux autres personnes lorsqu'elles me parlent de leurs problèmes.	1. I pay careful attention to people when they are telling me about their problems.	1. I pay special attention to other people when they tell me about their problems.	
2. If I see someone going through a difficult time, I try to be caring toward that person.	2. Si je vois que quelqu'un vit une période difficile, j'essaie d'être attentionné(e) envers cette personne.	2. If I see that someone is going through a difficult time, I try to be caring toward that person.	2. If I see that someone is going through a difficult time, I try to be considerate towards them.	
3. I am unconcerned with other people's problems.	3. Je ne suis pas préoccupé(e) par les problèmes des autres.	3. I am not preoccupied by the problems of others.	3. I am not concerned with other people's problems.	Je reste indiffèrent vis à vis les problèmes des autres.
4. I realize everyone feels down	4. Je suis conscient(e) que tout le	4. I am aware that everyone can	4. I am aware that everyone can	

Commented [A1]: The term 'préoccupé(e)' was changed to 'indiffèrent.' The initial French translation seemed to be interpreted as expressing a sentiment of "not being preoccupied by the problems of others," which could be interpreted in a positive light (e.g., healthy boundaries, holding others' concerns in mindful awareness), and did not capture the original meaning of the item as being indifferent/loading onto the indifference subscale.

sometimes, it is part of being human.	monde peut parfois se sentir déprimé(e), cela fait partie de la nature humaine.	sometimes feel depressed, and that it is part of human nature.	sometimes feel depressed; that is part of human nature.	
5. I notice when people are upset, even if they don't say anything.	5. Je remarque quand les gens sont contrarié(e)s, même s'ils/elles ne disent rien.	5. I notice when people are upset, even if they don't say anything.	5. I notice when people are upset, even if they don't say anything.	
6. I like to be there for others in times of difficulty.	6. J'aime être là pour les autres lorsqu'ils/elles traversent des périodes difficiles.	6. I like to be there for others when they are going through a difficult period.	6. I like being there for others when they are going through difficult times.	
7. I think little about the concerns of others.	7. Je me préoccupe peu des soucis des autres.	7. I am not very concerned by other people's worries.	7. I don't concern myself with others' worries.	Je passe peu de temps à penser aux soucis des autres.
8. I feel it's important to recognize that all people have weaknesses and no one's perfect.	8. Je crois qu'il est important de reconnaître que tout le monde a des faiblesses et que personne n'est parfait.	8. I think it's important to recognize that everyone has weaknesses and no one is perfect.	8. I believe it's important to recognize that everyone has weaknesses and that nobody is perfect.	
9. I listen patiently when people tell me their problems.	9. J'écoute patiemment lorsque les gens me parlent de leurs problèmes.	9. I patiently listen to people when they tell me about their problems.	9. I listen patiently when others tell me about their problems.	
10. My heart goes out to people who are unhappy.	10. Je me sens de tout cœur avec les personnes qui sont malheureuses.	10. I really feel others' pain.	10. I empathize with people who are unhappy.	
11. I try to avoid people who are experiencing a lot of pain.	11. J'essaie d'éviter les personnes qui éprouvent beaucoup de douleur.	11. I try to avoid people who are suffering badly.	11. I try to avoid people who suffer a lot of pain.	
12. I feel that suffering is just a part of the common human experience.	12. Je pense que la souffrance fait simplement partie de l'expérience humaine commune.	12. I think that suffering is part of the universal human experience.	12. I think that suffering is simply part of the shared human experience.	
13. When people tell me about their problems, I try to keep a balanced perspective on the situation.	13. Lorsque les gens me parlent de leurs problèmes, j'essaie de garder un point de vue objectif de la situation.	13. When people talk to me about their problems, I try to keep an objective perspective of the situation.	13. When people tell me about their problems, I try to remain objective towards the situation.	Lorsque les gens me parlent de leurs problèmes, j'essaie de garder un point de vue équilibré de la situation.
14. When others feel sadness, I try to comfort them.	14. Lorsque les autres ressentent de la tristesse, j'essaie de les réconforter.	14. When others feel sad, I try to comfort them.	14. When others are feeling sadness, I try to comfort them.	

Commented [A2]: The phrase 'Je me préoccupe peu' was changed to 'Je passe peu de temps' — similar reasoning to item 3. The revised item more accurately reflects the indifference subscale.

Commented [A3]: The term 'objectif' was replaced by 'équilibré' as this more accurately reflects the term 'balanced' from the original item.

15. I can't really connect with other people when they're suffering.	15. Je n'arrive pas vraiment à établir un lien avec les autres lorsqu'ils/elles souffrent.	15. I struggle to connect with people when they are suffering.	15. I cannot quite connect with others when they are suffering.	
16. Despite my differences with others, I know that everyone feels pain just like me.	16. Malgré mes différences avec les autres, je sais que, comme moi, tout le monde ressent de la douleur.	16. Despite our differences, I know that everyone experiences pain, just like I do.	16. Despite my differences with others, I know that, like me, everyone feels pain.	
Coding scheme<br>Kindness items: 2, 6, 10, 14<br>Common Humanity items: 4, 8, 12, 16<br>Mindfulness items: 1, 5, 9, 13<br>Indifference items (reverse-coded): 3, 7, 11, 15<br><br>To compute a total compassion score, take a grand mean of all items.	Système de codage<br>Items liés à la bienveillance : 2, 6, 10, 14<br>Items liés à l'expérience humaine commune : 4, 8, 12, 16<br>Items liés à la pleine conscience : 1, 5, 9, 13<br>Items liés à l'indifférence (cotation inverse) : 3, 7, 11, 15<br><br>Pour calculer un score total de compassion, prenez la moyenne de tous les items.	Scoring System:<br>Items measuring goodwill: 2, 6, 10, 14<br>Items measuring common human experience: 4, 8, 12, 16<br>Items measuring mindfulness: 1, 5, 9, 13<br>Items measuring indifference (reverse score): 3, 7, 11, 15<br><br>To obtain a final score, calculate the average score of all items.	Coding system<br>Items related to caring : 2, 6, 10, 14<br>Items related to the shared human experience: 4, 8, 12, 16<br>Items related to full consciousness: 1, 5, 9, 13<br>Items related to indifference (reverse scoring) : 3, 7, 11, 15<br><br>To calculate a total compassion score, take the average of all items.	
